# Supplementary material for: Observation of Chiral-selective room-temperature phosphorescence enhancement via chirality-dependent energy transfer
Source: Nat Commun. 2023 Mar 18;14:1514. doi: 10.1038/s41467-023-37157-w (PMC10024683; doi:10.1038/s41467-023-37157-w)
Supplement: Supplementary file 1 — Supplementary Information [file 41467_2023_37157_MOESM1_ESM.pdf]

Supplementary Information for

Observation of Chiral-Selective Room-Temperature Phosphorescence Enhancement via Chirality-Dependent Energy Transfer

Biao Chen[1]*, Wenhuan Huang[1,2] and Guoqing Zhang[1,2]*

[1]Hefei National Research Center for Physical Science at the Microscale, University of Science and Technology of China, 96 Jinzhai Rd, Hefei, Anhui 230026 China. [2]Hefei National Laboratory, University of Science and Technology of China, Hefei 230088, China
E-mail: gzhang@ustc.edu.cn; biaochen@ustc.edu.cn.

Contents

1. Synthesis Procedure..2

2. Supplementary Figures and Tables..9

3. Crystal data..27

4. Lifetime spectra...30

5. EPR Spectra...33

6. Solid-state Absorption..34

7. Nuclear Magnetic Resonance (NMR) and High-Resolution Mass (HRM) Spectra......35

1. Synthesis Procedure

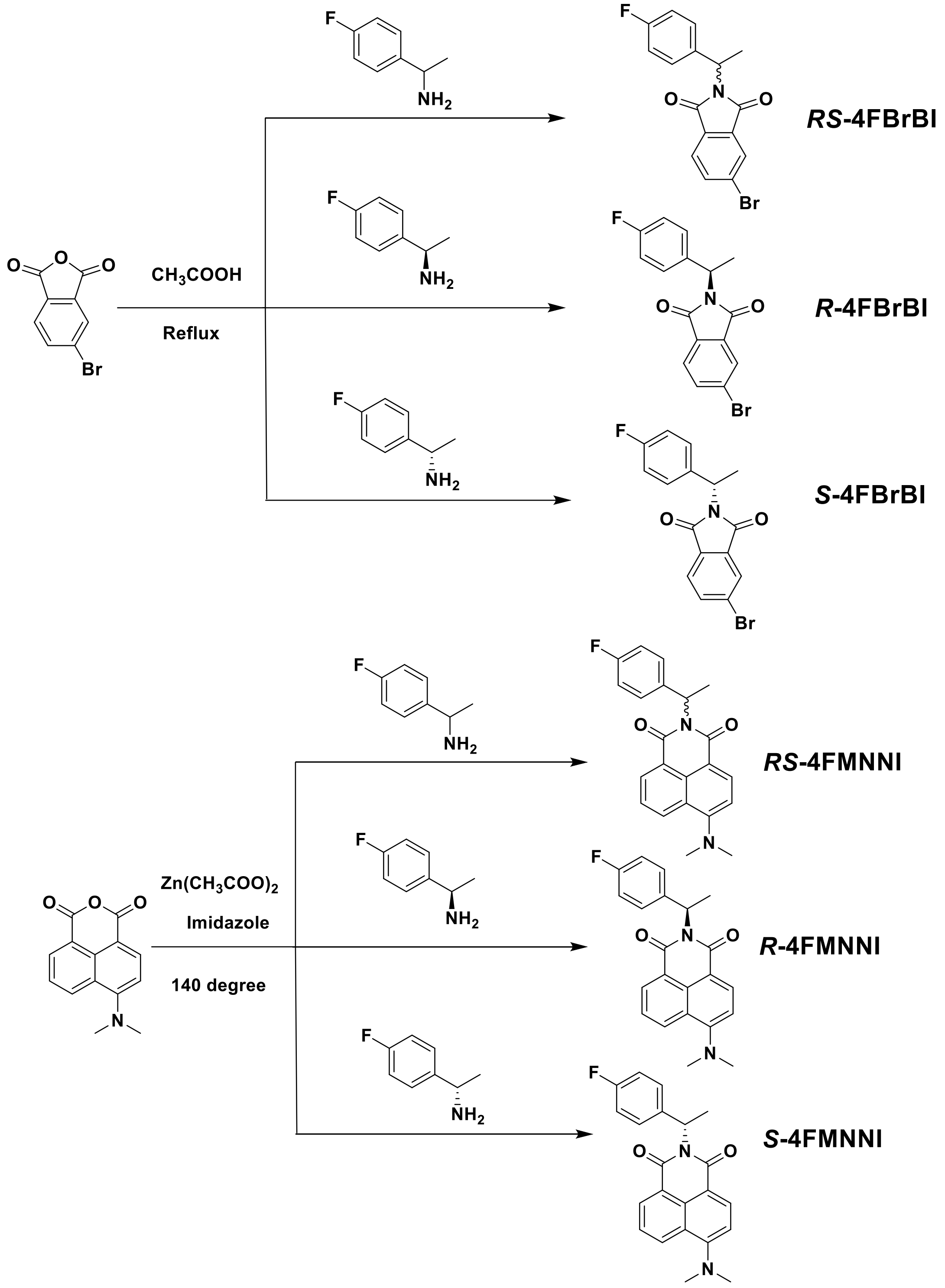

Supplementary Fig 1. Synthetic routes of the target compounds.

Preparation of 5-bromo-2-(1-(4-fluorophenyl)ethyl)isoindoline-1,3-dione (*RS*-4FBrBI) 4-bromophthalic anhydride (2.27 g, 10 mmol) and 1-(4-fluorophenyl)ethanamine (1.53 g, 11

mmol) were added to a round-bottomed flask containing 10 ml CH_3COOH and the solution was stirred at 120 °C overnight. The mixture was cooled to room temperature, filtrated, washed with water, and the crude product was then purified via silica gel chromatography with DCM/petroleum ether (v/v = 1:5) as eluent at least twice giving pure ***RS*-4FBrBI** as a white solid (2.46 g, 71%). ^{1}H NMR (400 MHz, d-DMSO) δ = 8.08-8.00 (m, 2H), 7.79 (d, *J*=8.5, 1H), 7.44 (dd, *J*=8.6, 5.5, 2H), 7.15 (t, *J*=8.8, 2H), 5.44 (q, *J*=7.2, 1H), 1.80 (d, *J*=7.3, 3H). ^{13}C NMR (101 MHz, d-DMSO) δ = 167.42, 166.84, 163.02, 160.60, 137.67, 137.05, 137.02, 133.88, 130.82, 129.34, 129.26, 128.53, 126.48, 125.44, 115.69, 115.48, 48.81, 18.02. HRMS (EI+) m/z 346.99499 [$(M)^+$; calculated mass for $C_{16}H_{11}BrFNO_2^+$: 346.99517 amu]. Elemental analysis (calcd., found for $C_{16}H_{11}BrFNO_2$): C (55.20, 55.17), H (3.18, 3.25).

Preparation of (*R*)-5-bromo-2-(1-(4-fluorophenyl)ethyl)isoindoline-1,3-dione (*R*-4FBrBI)

The synthesis and purification of ***R*-4FBrBI** were similar to ***RS*-4FBrBI**. 4-bromophthalic anhydride (2.27 g, 10 mmol) and (*R*)-1-(4-fluorophenyl)ethanamine (1.53 g, 11 mmol) were added to a round-bottomed flask containing 10 ml CH_3COOH and the solution was stirred at 120 °C overnight. The mixture was cooled to room temperature, filtrated, washed with water, and the crude product was then purified via silica gel chromatography with DCM/petroleum ether (v/v = 1:5) as eluent at least twice giving pure ***R*-4FBrBI** (2.39 g, 69%) as white solid. ^{1}H NMR (400 MHz, d-DMSO) δ = 8.04 (d, *J*=6.6, 2H), 7.79 (d, *J*=8.1, 1H), 7.45 (d, *J*=5.4, 2H), 7.15 (t, *J*=8.5, 2H), 5.45 (d, *J*=7.0, 1H), 1.81 (d, *J*=7.0, 3H). ^{13}C NMR (101 MHz, d-DMSO) δ = 167.42, 166.84, 163.02, 160.60, 137.67, 137.05, 137.02, 133.87, 130.82, 129.34, 129.26, 128.52, 126.47, 125.44, 115.69, 115.47, 48.81, 18.02. HRMS (EI+) m/z 346.99489 [$(M)^+$; calculated mass for $C_{16}H_{11}BrFNO_2^+$: 346.99517 amu]. Elemental analysis (calcd., found for $C_{16}H_{11}BrFNO_2$): C (55.20, 55.13), H (3.18, 3.22).

Preparation of (*S*)-5-bromo-2-(1-(4-fluorophenyl)ethyl)isoindoline-1,3-dione (*S*-4FBrBI)

The same synthesis method as ***RS*-4FBrBI**. 4-bromophthalic anhydride (2.27 g, 10 mmol) and (*S*)-1-(4-fluorophenyl)ethanamine (1.53 g, 11 mmol) were added to a round-bottomed flask containing 10 ml CH_3COOH and the solution was stirred at 120 °C overnight. The mixture was cooled to room temperature, filtrated, washed with water, and the crude product was then purified via silica gel chromatography with DCM/petroleum ether (v/v = 1:5) as eluent at least twice afforded ***S*-4FBrBI** (2.26 g, 65%) as a white solid. ^{1}H NMR (400 MHz, d-DMSO) δ = 8.04 (d, *J*=6.5, 2H), 7.79 (d, *J*=8.3, 1H), 7.44 (dd, *J*=10.0, 3.4, 2H), 7.15 (t, *J*=8.7, 2H), 5.45 (dd, *J*=13.5, 6.5, 1H), 1.81 (d, *J*=7.1, 3H). ^{13}C NMR (101 MHz, d-DMSO) δ = 167.41, 166.83, 163.02, 160.60, 137.66, 137.04, 137.01, 133.87, 130.82, 129.34, 129.26, 128.52, 126.47, 125.44, 115.68, 115.47, 48.82, 18.02. HRMS (EI+) m/z 346.99475 [$(M)^+$; calculated mass for

$C_{16}H_{11}BrFNO_2^+$: 346.99517 amu]. Elemental analysis (calcd., found for $C_{16}H_{11}BrFNO_2$): C (55.20, 55.16), H (3.18, 3.29).

Preparation of 6-(dimethylamino)-2-(1-(4-fluorophenyl)ethyl)-1H-benzo[de]isoquinoline-1,3(2H)-dione (*RS*-4FMNNI) 6-(dimethylamino)benzo[de]isochromene-1,3-dione (0.24 g, 1 mmol), 1-(4-fluorophenyl)ethanamine (0.15 g, 1.1 mmol) and zinc acetate (0.27 g, 1.5 mmol) were added to a round-bottomed flask containing 4.0 g imidazole and the solution was stirred at 140 °C overnight. The mixture was cooled to room temperature, 20 ml water was added and extracted with CH_2Cl_2, and then purified via silica gel chromatography with ethyl acetate/petroleum ether (v/v = 1:10) as eluent at least twice giving pure ***RS*-4FMNNI** as a greenish-yellow solid (0.22 g, 61%). ^{1}H NMR (400 MHz, d-DMSO) δ = 8.51 (d, *J*=8.5, 1H), 8.42 (d, *J*=7.3, 1H), 8.32 (d, *J*=8.3, 1H), 7.75 (t, *J*=7.9, 1H), 7.39 (dd, *J*=8.3, 5.7, 2H), 7.21 (d, *J*=8.3, 1H), 7.10 (t, *J*=8.8, 2H), 6.34 (q, *J*=7.0, 1H), 3.09 (s, 6H), 1.87 (d, *J*=7.0, 3H). ^{13}C NMR (101 MHz, d-DMSO) δ = 164.13, 163.54, 162.51, 160.10, 157.07, 137.92, 137.89, 132.99, 132.06, 131.21, 130.25, 128.91, 128.83, 125.50, 124.59, 123.07, 115.24, 115.03, 113.90, 113.46, 48.86, 44.84, 16.98. HRMS (ESI+) m/z 363.14993 [(M+H)$^+$; calculated mass for $C_{22}H_{20}FN_2O_2^+$: 363.15033 amu]. Elemental analysis (calcd., found for $C_{22}H_{19}FN_2O_2$): C (72.91, 72.82), H (5.28, 5.25).

Preparation of (*R*)-6-(dimethylamino)-2-(1-(4-fluorophenyl)ethyl)-1H-benzo[de]isoquinoline-1,3(2H)-dione (*R*-4FMNNI) The synthesis and purification of ***R*-4FMNNI** were similar to ***RS*-4FMNNI**. 6-(dimethylamino)benzo[de]isochromene-1,3-dione (0.24 g, 1 mmol), (*R*)-1-(4-fluorophenyl)ethanamine (0.15 g, 1.1 mmol) and zinc acetate (0.27 g, 1.5 mmol) were added to a round-bottomed flask containing 4.0 g imidazole and the solution was stirred at 140 °C overnight. The mixture was cooled to room temperature, 20 ml water was added and extracted with CH_2Cl_2, and then purified via silica gel chromatography with ethyl acetate/petroleum ether (v/v = 1:10) as eluent at least twice affording ***R*-4FMNNI** (0.25 g, 70%) as a greenish-yellow solid. ^{1}H NMR (400 MHz, d-DMSO) δ = 8.50 (d, *J*=8.5, 1H), 8.42 (d, *J*=7.3, 1H), 8.32 (d, *J*=8.3, 1H), 7.74 (t, *J*=7.9, 1H), 7.40 (dd, *J*=8.2, 5.8, 2H), 7.20 (d, *J*=8.3, 1H), 7.11 (t, *J*=8.8, 2H), 6.35 (q, *J*=6.9, 1H), 3.09 (s, 6H), 1.87 (d, *J*=7.1, 3H). ^{13}C NMR (101 MHz, d-DMSO) δ = 164.12, 163.53, 162.51, 160.10, 157.05, 137.91, 137.88, 132.97, 132.03, 131.19, 130.23, 128.92, 128.83, 125.47, 124.57, 123.06, 115.23, 115.02, 113.90, 113.44, 48.86, 44.82, 16.97. HRMS (ESI+) m/z 363.15015 [(M+H)$^+$; calculated mass for $C_{22}H_{20}FN_2O_2^+$: 363.15033 amu]. Elemental analysis (calcd., found for $C_{22}H_{19}FN_2O_2$): C (72.91, 72.79), H (5.28, 5.40).

Preparation of (*S*)-6-(dimethylamino)-2-(1-(4-fluorophenyl)ethyl)-1H-benzo[de]isoquinoline-1,3(2H)-dione (*S*-4FMNNI) 6-(dimethylamino)benzo[de]isochromene-1,3-dione (0.24 g, 1 mmol), (*S*)-1-(4-fluorophenyl)ethanamine (0.15 g, 1.1 mmol) and zinc acetate (0.27 g, 1.5 mmol) were added to a round-bottomed flask containing 4.0 g imidazole and the solution was stirred at 140 °C overnight. The mixture was cooled to room temperature, 20 ml water was added and extracted with CH_2Cl_2, and then purified via silica gel chromatography with ethyl acetate/petroleum ether (v/v = 1:10) as eluent at least twice affording ***S*-4FMNNI** (0.24 g, 66%) as a greenish-yellow solid. ^{1}H NMR (400 MHz, d-DMSO) δ = 8.50 (d, *J*=8.5, 1H), 8.42 (d, *J*=7.2, 1H), 8.32 (d, *J*=8.3, 1H), 7.74 (t, *J*=7.9, 1H), 7.40 (dd, *J*=8.4, 5.6, 2H), 7.20 (d, *J*=8.3, 1H), 7.11 (t, *J*=8.8, 2H), 6.35 (q, *J*=7.0, 1H), 3.09 (s, 6H), 1.87 (d, *J*=7.1, 3H). ^{13}C NMR (101 MHz, d-DMSO) δ = 164.12, 163.53, 162.51, 160.10, 157.05, 137.91, 137.88, 132.97, 132.03, 131.19, 130.24, 128.91, 128.83, 125.48, 124.57, 123.07, 115.23, 115.02, 113.90, 113.44, 48.86, 44.82, 16.98. HRMS (ESI+) m/z 363.15009 [$(M+H)^+$; calculated mass for $C_{22}H_{20}FN_2O_2^+$: 363.15033 amu]. Elemental analysis (calcd., found for $C_{22}H_{19}FN_2O_2$): C (72.91, 72.80), H (5.28, 5.35).

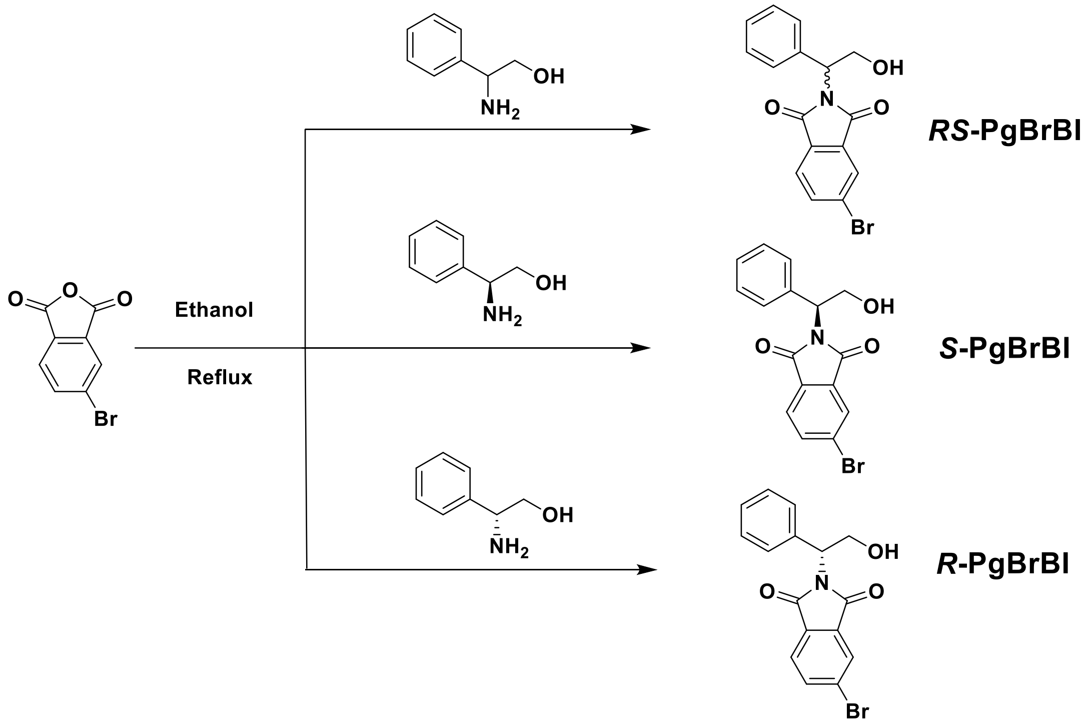

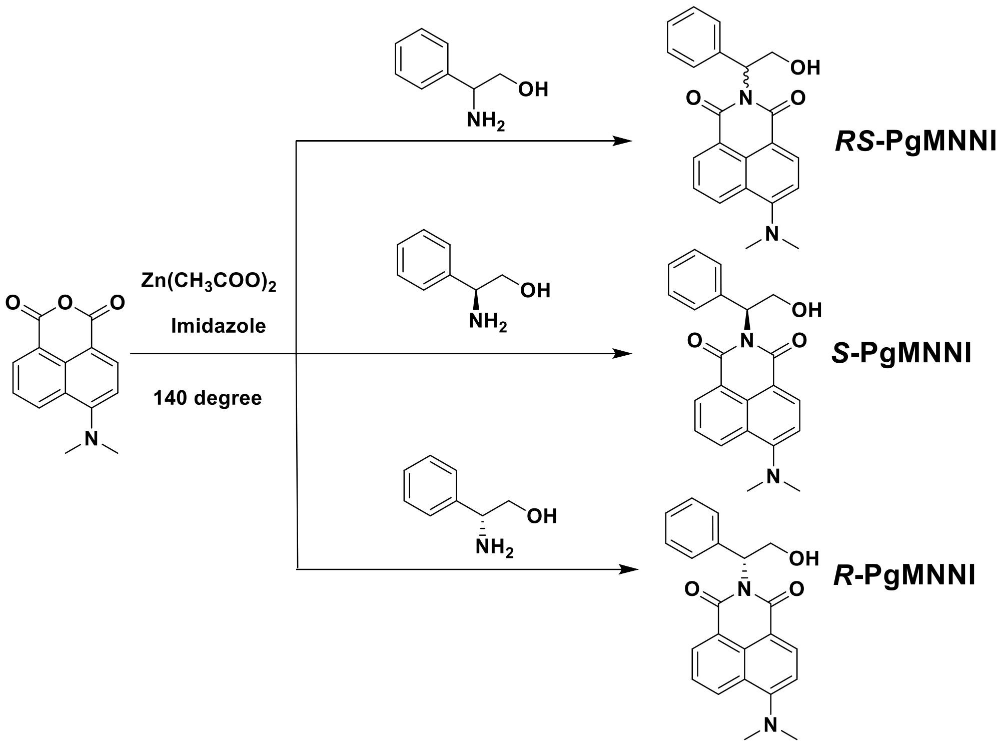

Supplementary Fig 2. Synthetic routes of the target compounds

Preparation of 5-bromo-2-(2-hydroxy-1-phenylethyl)isoindoline-1,3-dione (*RS*-PgBrBI) 4-bromophthalic anhydride (2.27 g, 10 mmol) and 2-amino-2-phenylethanol (0.94 g, 10.5 mmol) were added to a round-bottomed flask containing 15 ml ethanol and the solution was stirred at 100 °C overnight. The mixture was cooled to room temperature, filtrated, washed with water and ethanol, and then purified via silica gel chromatography with ethyl acetate/petroleum ether (v/v = 1:5) as eluent at least twice giving pure ***RS*-PgBrBI** as a white solid (2.69 g, 78%). ^{1}H NMR (400 MHz, d-DMSO) δ = 8.20-7.96 (m, 2H), 7.82 (d, *J*=7.9, 1H), 7.45-7.38 (m, 2H), 7.38-7.31 (m, 2H), 7.31-7.24 (m, 1H), 5.33 (dd, *J*=9.9, 5.6, 1H), 5.17 (s, 1H), 4.39 (t, *J*=10.4, 1H), 4.07-3.93 (m, 1H). ^{13}C NMR (101 MHz, d-DMSO) δ = 167.92, 167.34, 137.76, 137.65, 133.81, 130.77, 128.97, 128.62, 128.15, 127.88, 126.52, 125.48, 60.46, 57.28. HRMS (ESI+) m/z 346.0074 [$(M+H)^+$; calculated mass for $C_{16}H_{13}BrNO_3^+$: 346.0073 amu]. Elemental analysis (calcd., found for $C_{16}H_{12}BrNO_3$): C (55.51, 55.37), H (3.49, 3.56).

Preparation of (*R*)-5-bromo-2-(2-hydroxy-1-phenylethyl)isoindoline-1,3-dione (*R*-PgBrBI) The same synthesis method as ***RS*-PgBrBI**. 4-bromophthalic anhydride (2.27 g, 10 mmol) and (*R*)-2-amino-2-phenylethanol (0.94 g, 10.5 mmol) were added to a round-bottomed flask containing 15 ml ethanol and the solution was stirred at 100 °C overnight. The mixture was cooled to room temperature, filtrated, washed with water and ethanol, and then purified via silica gel chromatography with ethyl acetate/petroleum ether (v/v = 1:5) as eluent at least twice affording pure ***R*-PgBrBI** (2.62 g, 76%) as a white solid. ^{1}H NMR (400 MHz, d-DMSO) δ

= 8.12-8.01 (m, 2H), 7.82 (d, *J*=7.9, 1H), 7.44-7.37 (m, 2H), 7.34 (dd, *J*=9.9, 4.8, 2H), 7.31-7.25 (m, 1H), 5.32 (dd, *J*=9.9, 5.6, 1H), 5.16 (t, *J*=5.9, 1H), 4.38 (td, *J*=10.5, 5.4, 1H), 4.06-3.93 (m, 1H). ^{13}C NMR (101 MHz, d-DMSO) δ = 167.94, 167.36, 137.77, 137.62, 133.80, 130.75, 128.99, 128.64, 128.16, 127.88, 126.53, 125.49, 60.44, 57.25. HRMS (ESI+) m/z 346.0076 [$(M+H)^+$; calculated mass for $C_{16}H_{13}BrNO_3^+$: 346.0073 amu]. Elemental analysis (calcd., found for $C_{16}H_{12}BrNO_3$): C (55.51, 55.42), H (3.49, 3.50).

Preparation of (*S*)-5-bromo-2-(2-hydroxy-1-phenylethyl)isoindoline-1,3-dione (*S*-PgBrBI)

The same synthesis method as ***RS*-PgBrBI**. 4-bromophthalic anhydride (2.27 g, 10 mmol) and (*S*)-1-(4-fluorophenyl)ethanamine (0.94 g, 10.5 mmol) were added to a round-bottomed flask containing 15 ml ethanol and the solution was stirred at 100 °C overnight. The mixture was cooled to room temperature, filtrated, washed with water and ethanol, and then purified via silica gel chromatography with ethyl acetate/petroleum ether (v/v = 1:5) as eluent at least twice affording ***S*-PgBrBI** (2.52 g, 73%) as white solid. ^{1}H NMR (400 MHz, d-DMSO) δ = 8.11-8.02 (m, 2H), 7.82 (d, *J*=7.9, 1H), 7.44-7.38 (m, 2H), 7.38-7.31 (m, 2H), 7.29 (ddd, *J*=7.1, 3.5, 1.2, 1H), 5.33 (dd, *J*=9.9, 5.6, 1H), 5.17 (t, *J*=5.8, 1H), 4.39 (td, *J*=10.7, 5.2, 1H), 4.00 (dt, *J*=11.3, 5.8, 1H). ^{13}C NMR (101 MHz, d-DMSO) δ = 167.92, 167.34, 137.76, 137.65, 133.81, 130.77, 128.98, 128.62, 128.15, 127.88, 126.52, 125.48, 60.46, 57.27. HRMS (ESI+) m/z 367.9895 [$(M+Na)^+$; calculated mass for $C_{16}H_{12}BrNNaO_3^+$: 367.9893 amu]. Elemental analysis (calcd., found for $C_{16}H_{12}BrNO_3$): C (55.51, 55.53), H (3.49, 3.68).

Preparation of 6-(dimethylamino)-2-(2-hydroxy-1-phenylethyl)-1H-benzo[de]isoquinoline-1,3(2H)-dione (*RS*-PgMNNI)

6-(dimethylamino)benzo[de]isochromene-1,3-dione (0.24 g, 1 mmol), 1-(4-fluorophenyl)ethanamine (0.15 g, 1.1 mmol) and zinc acetate (0.27 g, 1.5 mmol) were added to a round-bottomed flask containing 4.0 g imidazole and the solution was stirred at 140 °C overnight. The mixture was cooled to room temperature, and then 20 ml water was added, extracted with CH_2Cl_2, and purified via silica gel chromatography with ethyl acetate/petroleum ether (v/v = 1:3) as eluent at least twice giving pure ***RS*-PgMNNI** as a yellow solid (0.24 g, 66%). ^{1}H NMR (400 MHz, d-DMSO) δ = 8.50 (d, *J*=8.5, 1H), 8.44 (d, *J*=6.8, 1H), 8.34 (d, *J*=8.0, 1H), 7.75 (t, *J*=7.9, 1H), 7.40 (d, *J*=7.6, 2H), 7.30 (t, *J*=7.5, 2H), 7.26-7.14 (m, 2H), 6.28 (t, *J*=7.0, 1H), 5.04 (t, *J*=5.6, 1H), 4.40 (dd, *J*=11.8, 5.4, 2H), 3.08 (s, 6H). ^{13}C NMR (101 MHz, d-DMSO) δ = 164.60, 164.01, 156.99, 139.36, 132.95, 131.96, 131.18, 130.31, 128.54, 127.57, 127.18, 125.48, 124.58, 123.14, 114.02, 113.46, 61.01, 56.66, 44.82. HRMS (ESI+) m/z 361.15472 [$(M+H)^+$; calculated mass for $C_{22}H_{21}N_2O_3^+$: 361.15467 amu]. Elemental analysis (calcd., found for $C_{22}H_{20}N_2O_3$): C (73.32, 73.19), H (5.59, 5.70).

Preparation of (*R*)-6-(dimethylamino)-2-(2-hydroxy-1-phenylethyl)-1H-benzo[de]isoquinoline-1,3(2H)-dione (*R*-PgMNNI) A similar synthesis method as ***RS*-PgMNNI**. 6-(dimethylamino)benzo[de]isochromene-1,3-dione (0.24 g, 1 mmol), (*R*)-1-(4-fluorophenyl)ethanamine (0.15 g, 1.1 mmol) and zinc acetate (0.27 g, 1.5 mmol) were added to a round-bottomed flask containing 4.0 g imidazole and the solution was stirred at 140 °C overnight. The mixture was cooled to room temperature, and then 20 ml water was added, extracted with CH_2Cl_2, and purified via silica gel chromatography with ethyl acetate/petroleum ether (v/v = 1:3) as eluent at least twice giving pure ***R*-PgMNNI** (0.26 g, 71%) as a yellow solid. ^{1}H NMR (400 MHz, d-DMSO) δ = 8.52 (d, *J*=8.5, 1H), 8.44 (d, *J*=6.8, 1H), 8.33 (d, *J*=7.8, 1H), 7.76 (t, *J*=7.9, 1H), 7.38 (d, *J*=7.7, 2H), 7.29 (t, *J*=7.5, 2H), 7.21 (dd, *J*=7.7, 5.6, 2H), 6.26 (t, *J*=7.0, 1H), 5.05 (s, 1H), 4.38 (d, *J*=7.2, 2H), 3.09 (s, 6H). ^{13}C NMR (101 MHz, d-DMSO) δ = 164.61, 164.01, 157.02, 139.34, 132.97, 131.99, 131.20, 130.31, 128.55, 127.54, 127.19, 125.50, 124.58, 123.12, 113.98, 113.47, 60.99, 56.64, 44.83. HRMS (ESI+) m/z 361.15454 [$(M+H)^+$; calculated mass for $C_{22}H_{21}N_2O_3^+$: 361.15467 amu]. Elemental analysis (calcd., found for $C_{22}H_{20}N_2O_3$): C (73.32, 73.26), H (5.59, 5.74).

Preparation of (*S*)-6-(dimethylamino)-2-(2-hydroxy-1-phenylethyl)-1H-benzo[de]isoquinoline-1,3(2H)-dione (*S*-PgMNNI) A similar synthesis method as ***RS*-PgMNNI**. 6-(dimethylamino)benzo[de]isochromene-1,3-dione (0.24 g, 1 mmol), (*S*)-1-(4-fluorophenyl)ethanamine (0.15 g, 1.1 mmol) and zinc acetate (0.27 g, 1.5 mmol) were added to a round-bottomed flask containing 4.0 g imidazole and the solution was stirred at 140 °C overnight. The mixture was cooled to room temperature, and then 20 ml water was added, extracted with CH_2Cl_2, and purified via silica gel chromatography with ethyl acetate/petroleum ether (v/v = 1:3) as eluent at least twice giving pure ***S*-PgMNNI** (0.23 g, 63%) as a yellow solid. ^{1}H NMR (400 MHz, d-DMSO) δ = 8.50 (d, *J*=8.4, 1H), 8.44 (d, *J*=6.8, 1H), 8.33 (d, *J*=8.0, 1H), 7.75 (t, *J*=7.9, 1H), 7.40 (d, *J*=7.5, 2H), 7.30 (t, *J*=7.5, 2H), 7.22 (dd, *J*=10.2, 8.0, 2H), 6.28 (t, *J*=7.0, 1H), 5.05 (t, *J*=5.2, 1H), 4.40 (dd, *J*=11.2, 5.0, 2H), 3.09 (s, 6H). ^{13}C NMR (101 MHz, d-DMSO) δ = 164.59, 164.01, 156.99, 139.35, 132.95, 131.96, 131.18, 130.31, 128.55, 127.56, 127.19, 125.48, 124.58, 123.13, 114.00, 113.45, 61.00, 56.65, 44.82. HRMS (ESI+) m/z 361.1546 [$(M+H)^+$; calculated mass for $C_{22}H_{21}N_2O_3^+$: 361.15467 amu]. Elemental analysis (calcd., found for $C_{22}H_{20}N_2O_3$): C (73.32, 73.19), H (5.59, 5.66).

2. Supplementary Figures and Tables

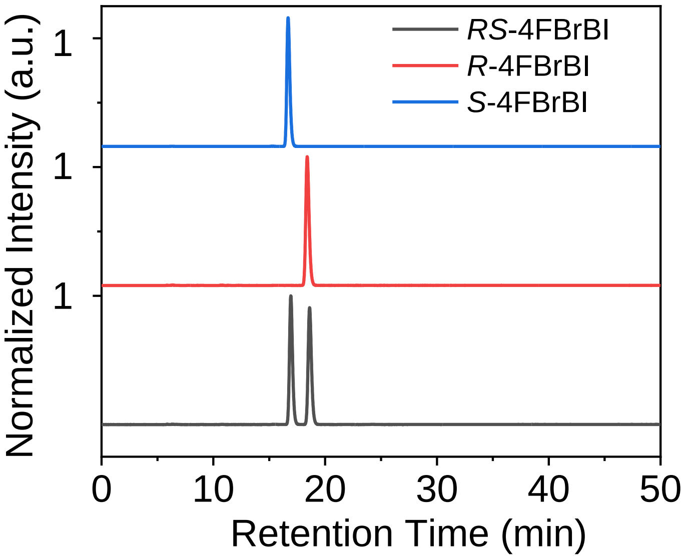

Supplementary Fig 3. Chiral high performance liquid chromatogram (Chiral HPLC) spectrum of the model compounds in ethanol (30 µM) with ethanol and n-hexane (30: 70) monitored at the onset absorption of 300 nm.

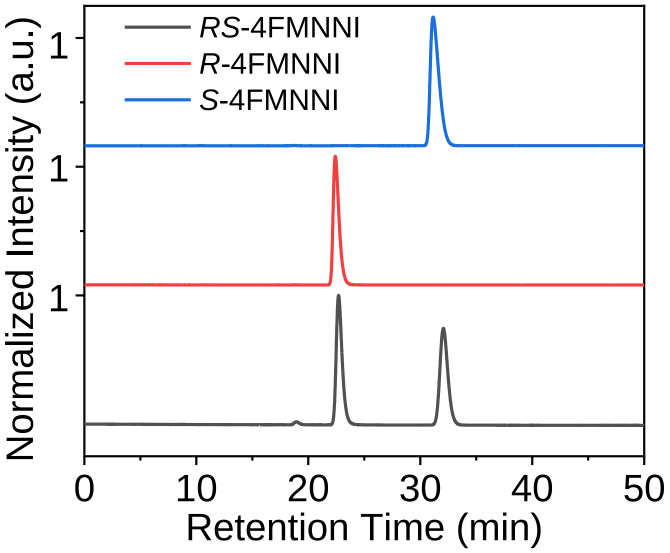

Supplementary Fig 4. Chiral high performance liquid chromatogram (Chiral HPLC) spectrum of the model compounds in ethanol (30 µM) with ethanol and n-hexane (100: 0) monitored at the onset absorption of 370 nm.

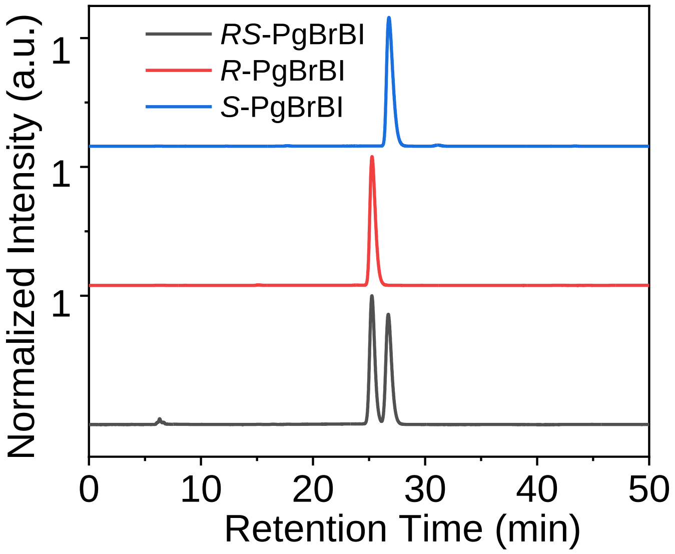

Supplementary Fig 5. Chiral high performance liquid chromatogram (Chiral HPLC) spectrum of the model compounds in ethanol (30 μM) with ethanol and n-hexane (40: 60) monitored at the onset absorption of 300 nm.

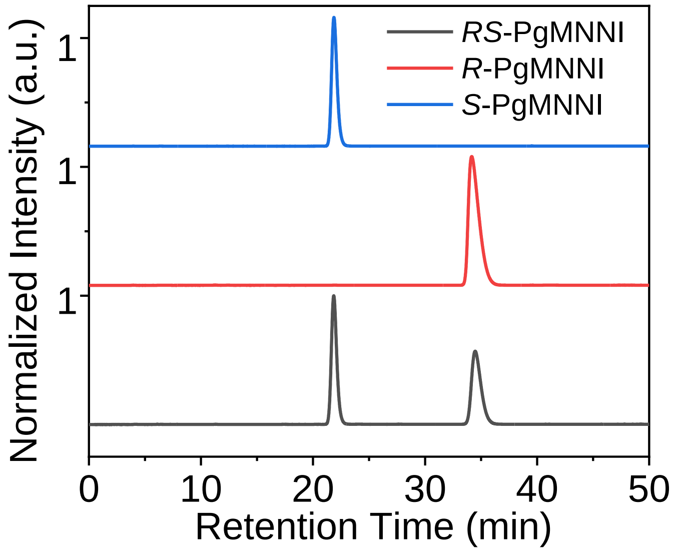

Supplementary Fig 6. Chiral high performance liquid chromatogram (Chiral HPLC) spectrum of the model compounds in ethanol (30 μM) with ethanol and n-hexane (70: 30) monitored at the onset absorption of 370 nm.

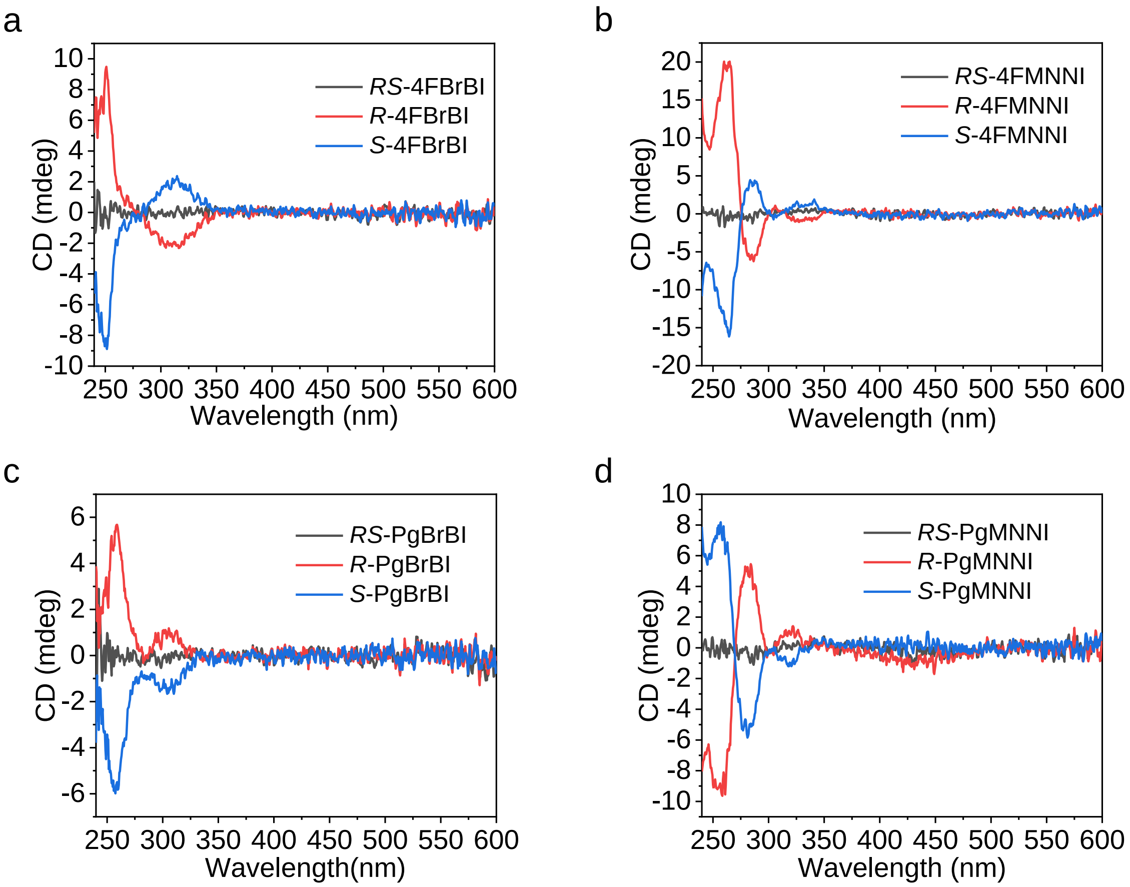

Supplementary Fig 7. Circular dichroism (CD) spectra of dilute solutions of model compounds in (Host molecules for **a** and **c**, and guest molecules for **b** and **d**) dichloromethane (0.1 mM).

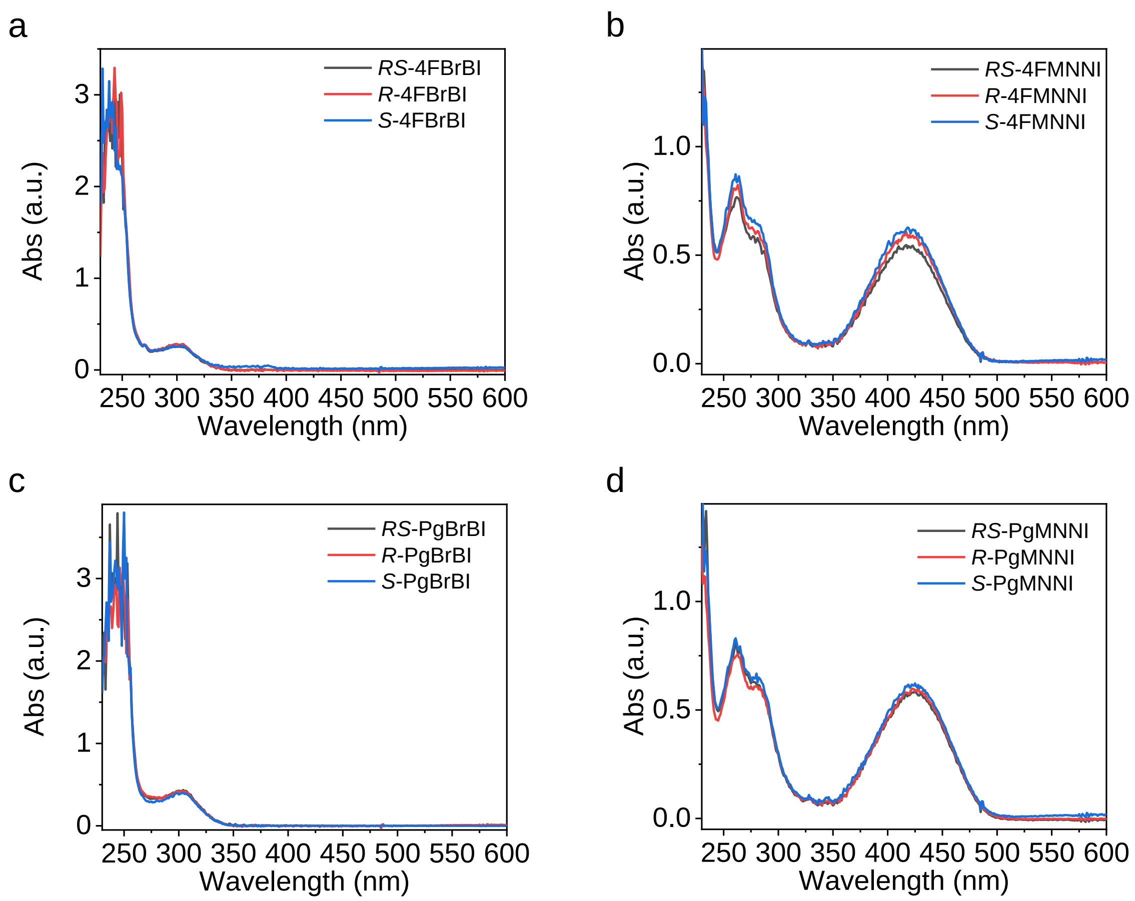

Supplementary Fig 8. UV absorption spectra in dichloromethane (DCM, 0.25 mM for **a** and **c**, 0.05 mM for **b** and **d**) at 298 K.

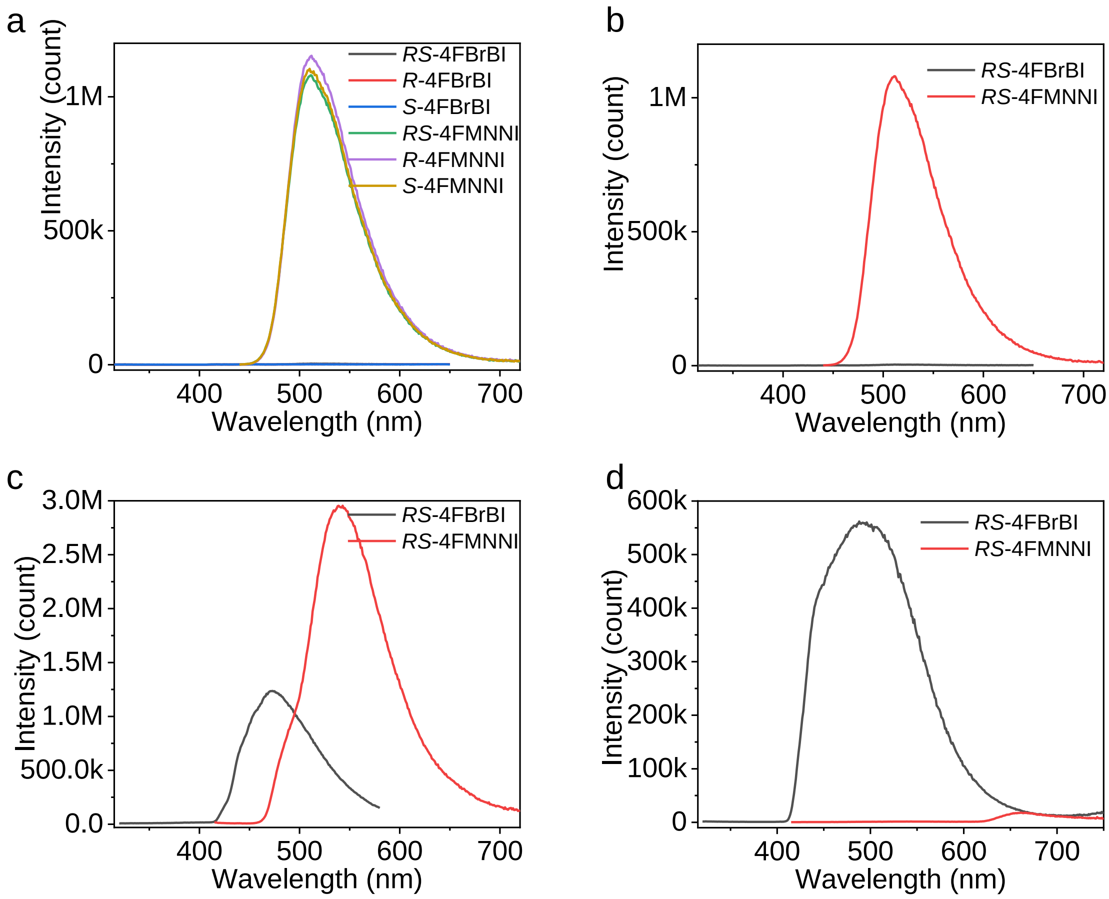

Supplementary Fig 9. a, Photoluminescence (PL) spectra in dichloromethane (DCM) (20 μM) at 298 K. **b,** PL spectra in DCM (20 μM) at 298 K (excited at absorption maxima). **c,** PL spectra and **d** delayed (Δt = 0.1 ms) emission in DCM at 77 K.

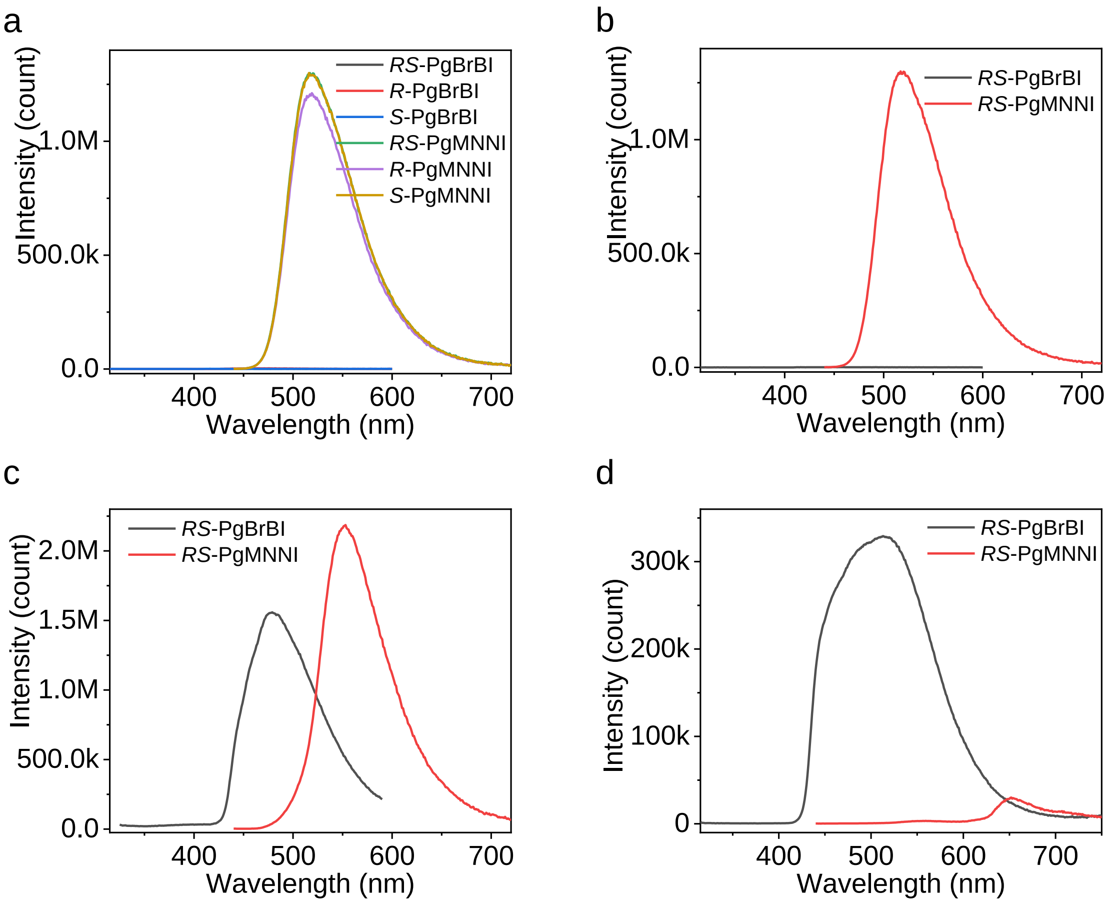

Supplementary Fig 10. a, Photoluminescence (PL) spectra in dichloromethane (DCM) (20 μM) at 298 K. **b,** PL spectra in DCM (20 μM) at 298 K (excited at absorption maxima). **c,** PL spectra and **d** delayed (Δt = 0.1 ms) emission in DCM at 77 K.

Supplementary Tab 1. Photoluminescence properties of representative molecules in dichloromethane (DCM).

Molecule	Absorption max[a] (nm)	ε (L*mol^{-1}*cm^{-1})	Emission max (nm)[b]	Lifetime[c]	Quantum yield (%)[d]
***RS*-4FBrBI**	300	1000	488[e]	6.50 ms	<0.1
***RS*-PgBrBI**	304	1700	493[e]	7.24 ms	<0.1
***RS*-4FMNNI**	420	10900	510	9.68 ns	75.7
***RS*-PgMNNI**	425	11700	518	9.69 ns	68.1

[a] UV absorption spectra in DCM ($5x10^{-5}$ M for **MNNI**, $2.5x10^{-4}$ M for **BrBI**). [b] Emission maxima of photoluminescence spectra excited at absorption maxima in DCM (RT for **MNNI**, 77 K for **BrBI**). [c] Apparent lifetime in DCM (naonoLED-370 for **MNNI**, spectraLED-370 for **BrBI**). [d] Absolute quantum yield from 400-650 nm in CH_2Cl_2 at room temperature. [e] 77 K.

Supplementary Tab 2. Photoluminescence properties of dopant samples at (10 ppm w/w) and pure host solids at room temperature.

Samples[a]	λ_F (nm)[b]	τ_F (ns)[c]	λ_{RTP} (nm)[d]	τ_{RTP} (ms)[e]	ep[f]
R@R	485	3.62	615	73.31	140
S@R	489	6.22	618	44.79	
S@S	484	5.41	617	70.55	155
R@S	489	5.50	618	41.94	
***R*-4FBrBI**	/[g]	/	482	0.62	/
***S*-4FBrBI**	/	/	482	0.85	/

[a] Guest-host molecular solids (w/w 10 ppm). [b] Fluorescence emission maxima obtained from the steady-state photoluminescence spectrum excited at 365 nm. [c] Apparent fluorescence lifetime (naonoLED-370). [d] Phosphorescence emission maxima of delayed emission spectra excited at 424 nm. [e] Apparent phosphorescence lifetime (spectraLED-370). [f] Enantiomeric RTP enhancement ratios (ep, $I_{S@S}/I_{R@S}$ or $I_{R@R}/I_{S@R}$). [g] Signal too weak to measure or does not exist.

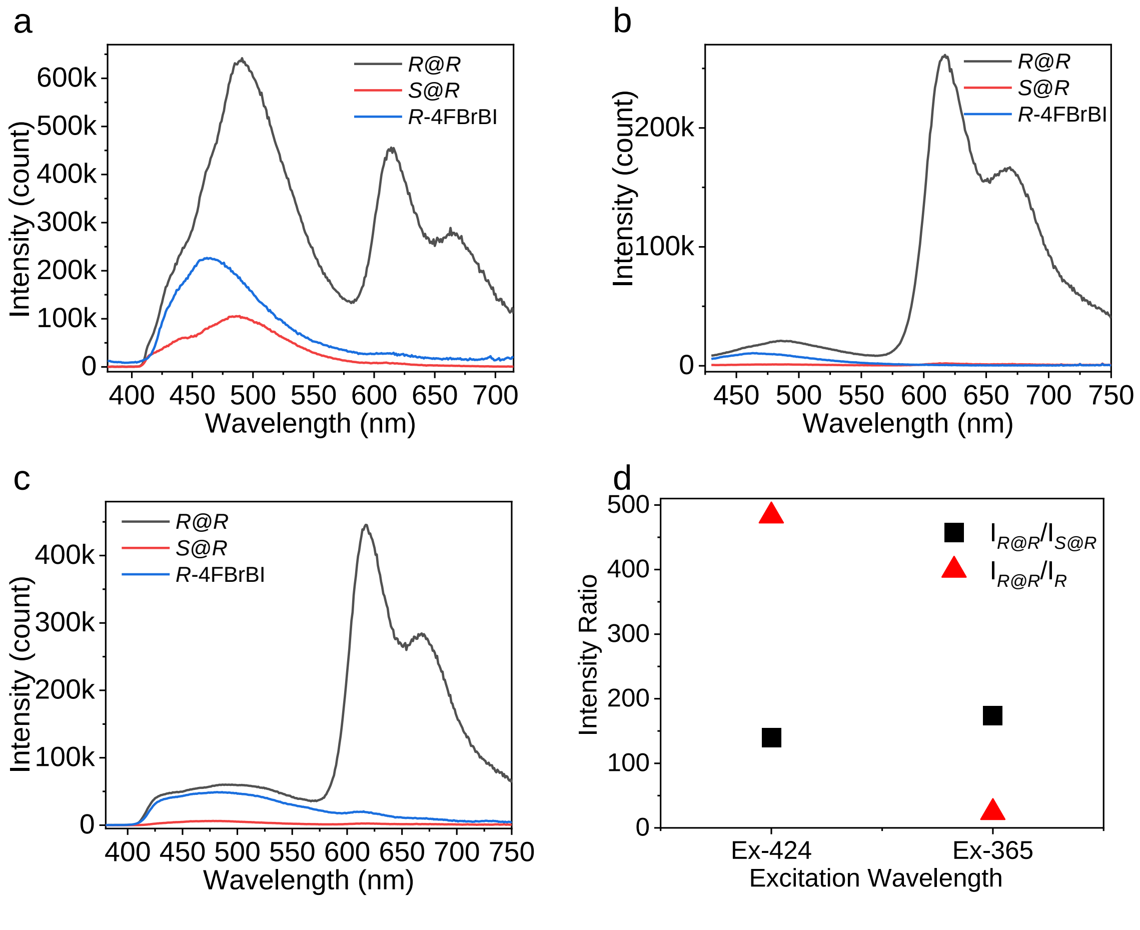

Supplementary Fig 11. a, Steady-state PL spectra of two chiral guests (w/w 10 ppm) in the ***R*-4FBrBI** host solid compared with pure ***R*-4FBrBI** solid at 298 K (λ_{ex} = 365 nm). Delayed emission (DE, Δt = 0.1 ms) spectra of two guests (w/w 10 ppm) in the ***R*-4FBrBI** solid compared with pure ***R*-4FBrBI** solid at 298 K (λ_{ex} = 424 nm for **b,** λ_{ex} = 365 nm for **c**). **d,** Ratio of intensity of DE emission at 620 nm.

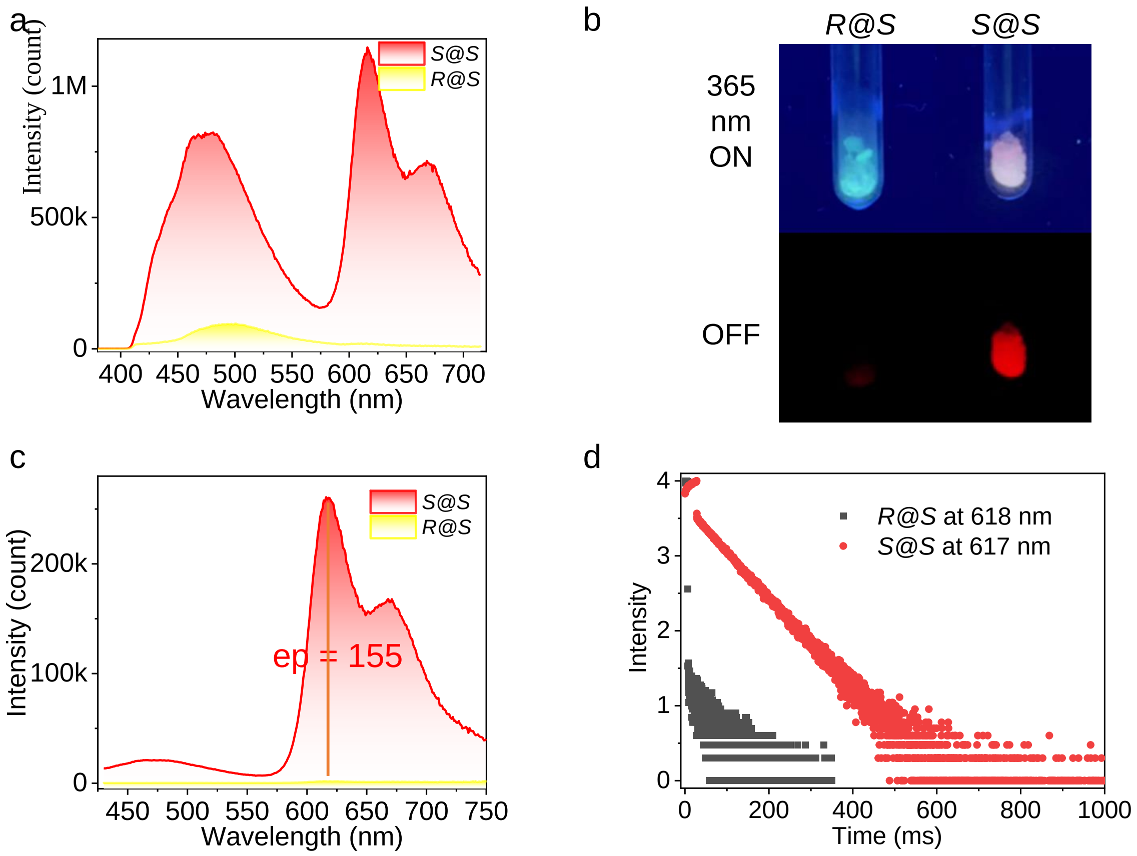

Supplementary Fig 12. a, Steady-state PL spectra of two chiral guests (w/w 10 ppm, red line for ***S*-4FMNNI** and yellow line for ***R*-4FMNNI**) in the ***S*-4FBrBI** host solid at 298 K (λ_{ex} = 365 nm). **b,** Photographs of combinations of two guests in ***S*-4FBrBI** during and immediately after 365-nm light irradiation. **c,** Delayed emission (DE, Δt = 0.1 ms) spectra of two guests (w/w 10 ppm) in the ***S*-4FBrBI** solid at 298 K (λ_{ex} = 424 nm). **d,** Time-resolved emission decay curves of ***S*-4FMNNI** and ***R*-4FMNNI** in solid ***S*-4FBrBI**.

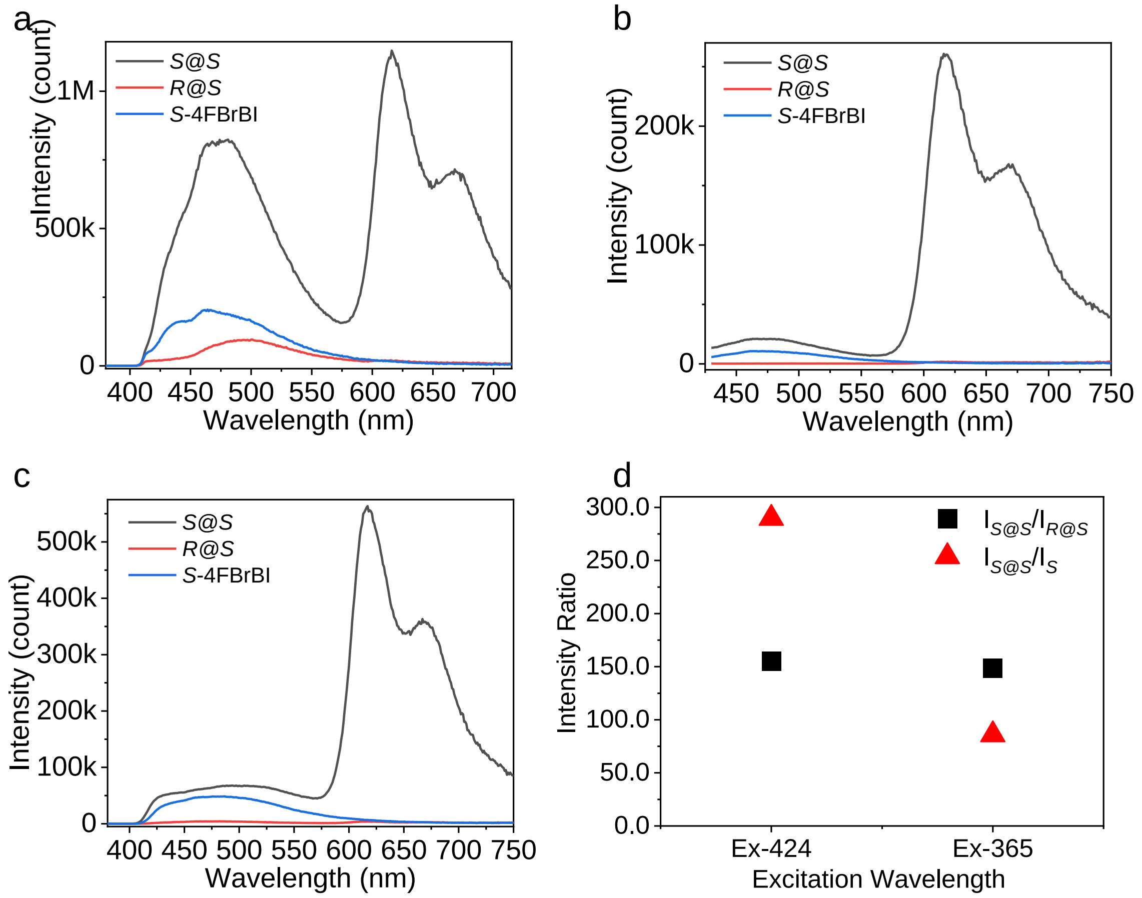

Supplementary Fig 13. a, Steady-state PL spectra of two chiral guests (w/w 10 ppm) in the ***S*-4FBrBI** host solid compared with pure ***S*-4FBrBI** solid at 298 K (λ_{ex} = 365 nm). Delayed emission (DE, Δt = 0.1 ms) spectra of two guests (w/w 10 ppm) in the ***S*-4FBrBI** solid compared with pure ***S*-4FBrBI** solid at 298 K (λ_{ex} = 424 nm for **b,** λ_{ex} = 365 nm for **c**). **d,** Ratio of intensity of DE emission at 620 nm.

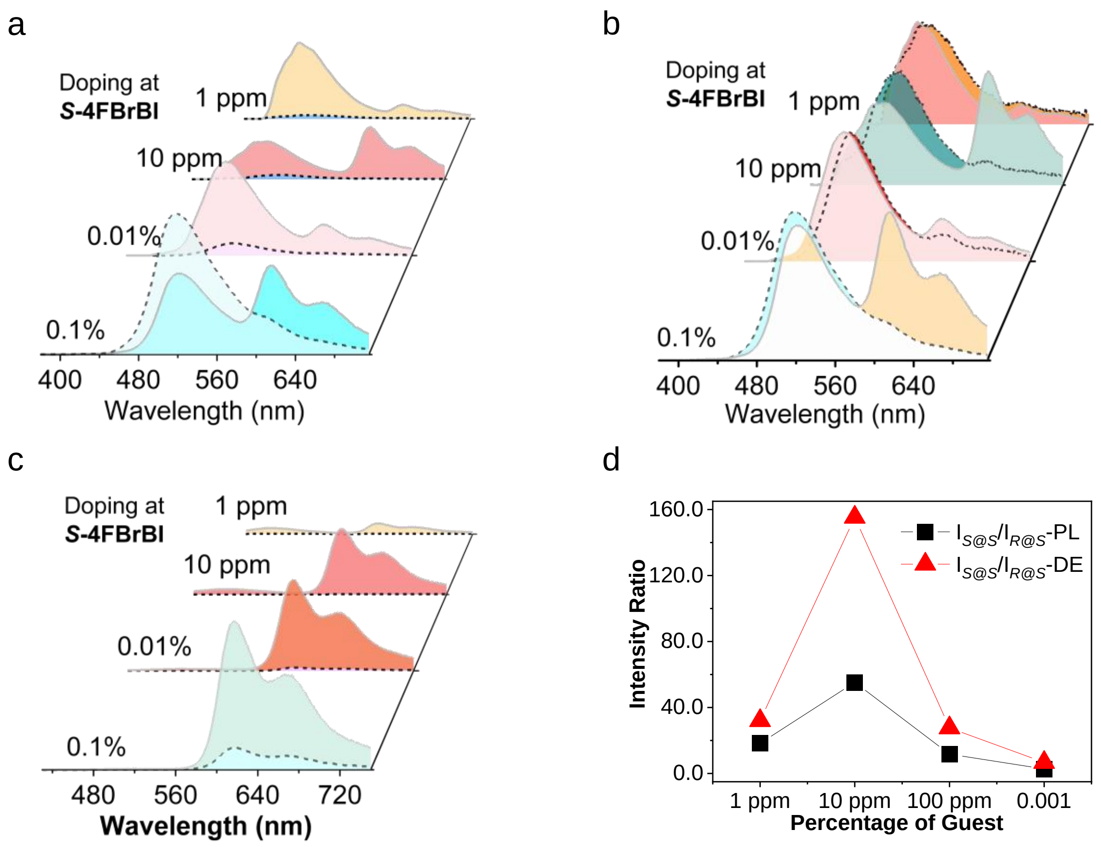

Supplementary Fig 14. a, Steady-state PL spectra of ***S*-4FMNNI** (solid borderline) or ***R*-4FMNNI** (dash borderline) dopants (w/w 1 ppm-0.1%) in ***S*-4FBrBI** solid in air at 298 K (λ_{ex} = 365 nm). **b,** Normalized PL spectra of **a**. **c,** Delayed emission (DE, Δt = 0.1 ms) spectra of ***S*-4FMNNI** (solid borderline) or ***R*-4FMNNI** (dash borderline) dopants (w/w 1 ppm-0.1%) in ***S*-4FBrBI** solid in air at 298 K (λ_{ex} = 424 nm). **d,** The ep value (intensity of PL emission at 620 nm or DE emission at 615 nm) vs. percentage of guest dopants (1 ppm-0.1%).

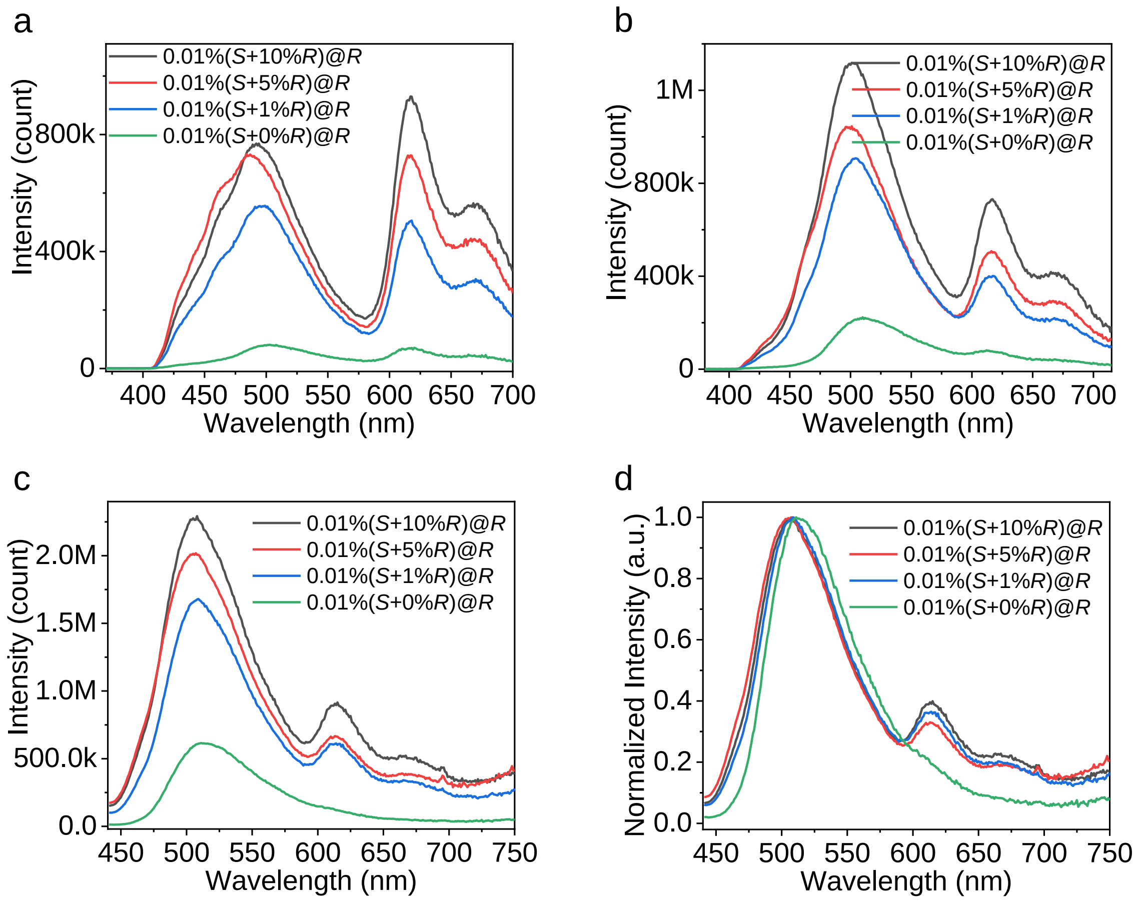

Supplementary Fig 15. PL spectra of ***R*-4FMNNI** dopants (0%-10%) in 0.01% ***S*-4FMNNI@*R*-4FBrBI** solid at air at 298 K (λ_{ex} = 355 nm for **a**, 365 nm for **b**, 424 nm for **c**). **d,** Normalized PL of **c**.

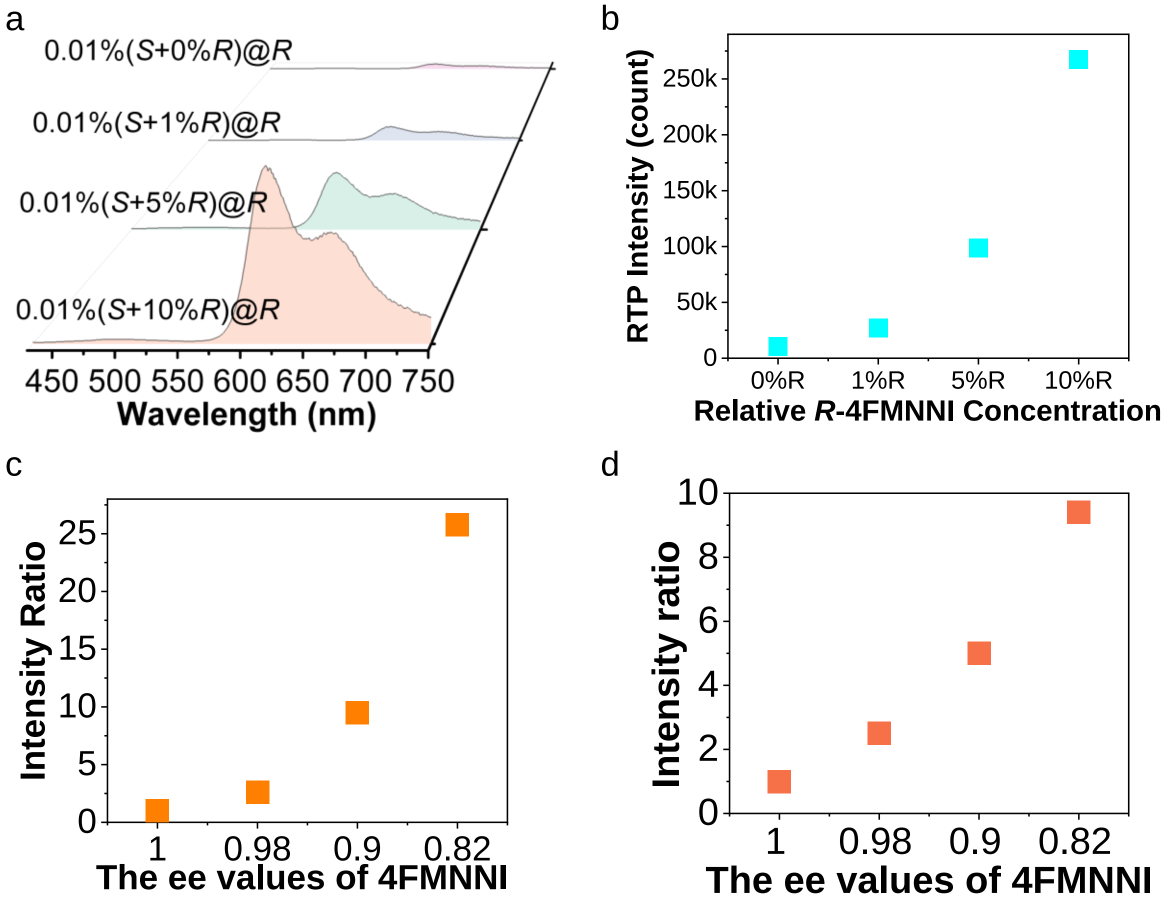

Supplementary Fig 16. a, Delayed emission (DE, Δt = 0.1 ms) spectra of ***R*-4FMNNI** dopants (0%-10%) in 0.01% ***S*-4FMNNI@*R*-4FBrBI** solid at air at 298 K (λ_{ex} = 424 nm). **b,** Intensity of DE emission at 618 nm in **a** vs. Percentage of ***R*-4FMNNI** dopants (0%-10%) (λ_{ex} = 424 nm). **c,** Intensity ratio of DE emission at 618 nm when take 0%**R** sample as 1 vs. **4FMNNI** enantiomeric excess (ee) values (λ_{ex} = 424 nm). **d,** Intensity ratio of DE emission at 618 nm (see also in Figure 2g) when take 0%**R** sample as 1 vs. **4FMNNI** ee values (λ_{ex} = 365 nm).

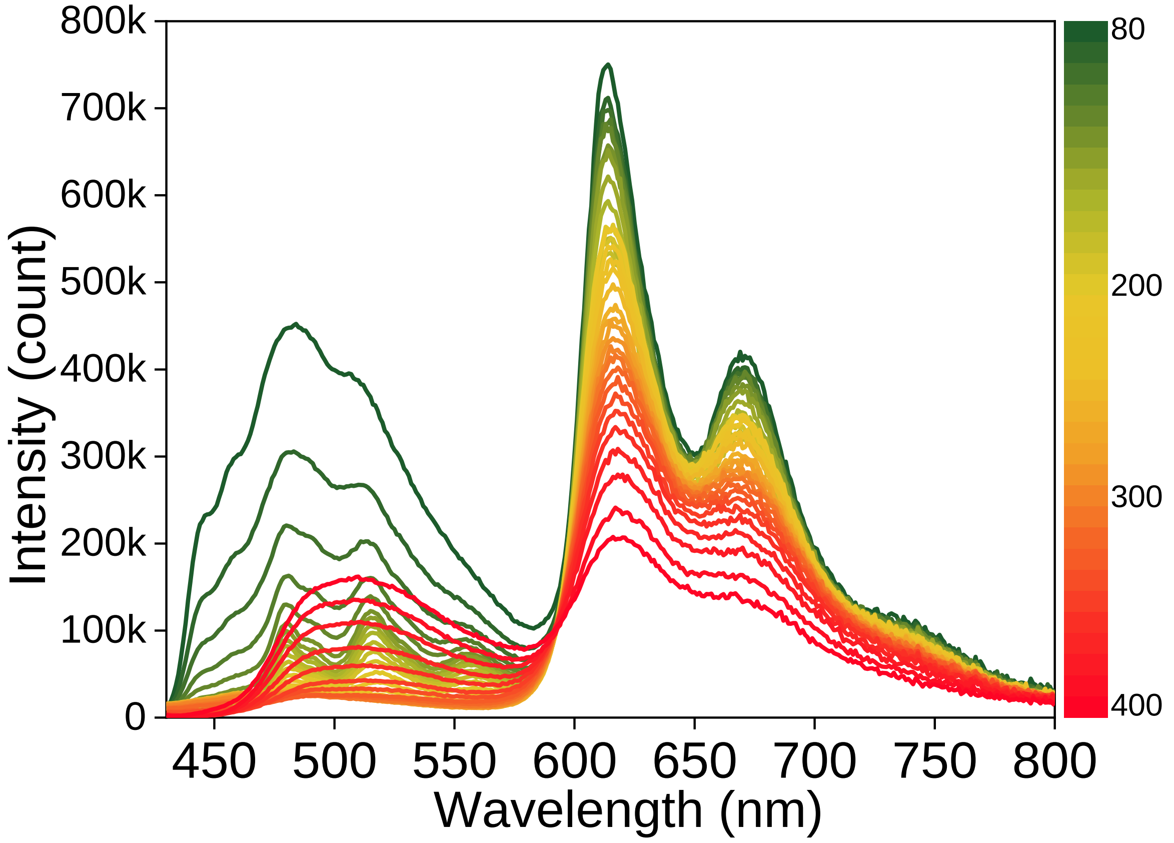

Supplementary Fig 17. Temperature-dependent delayed emission (Δt = 0.1 ms**,** TDDE) spectra of ***R*-4FMNNI@*R*-4FBrBI** (10 ppm, λ_{ex} = 424 nm), from 80 K to 400 K with **10 K interval**.

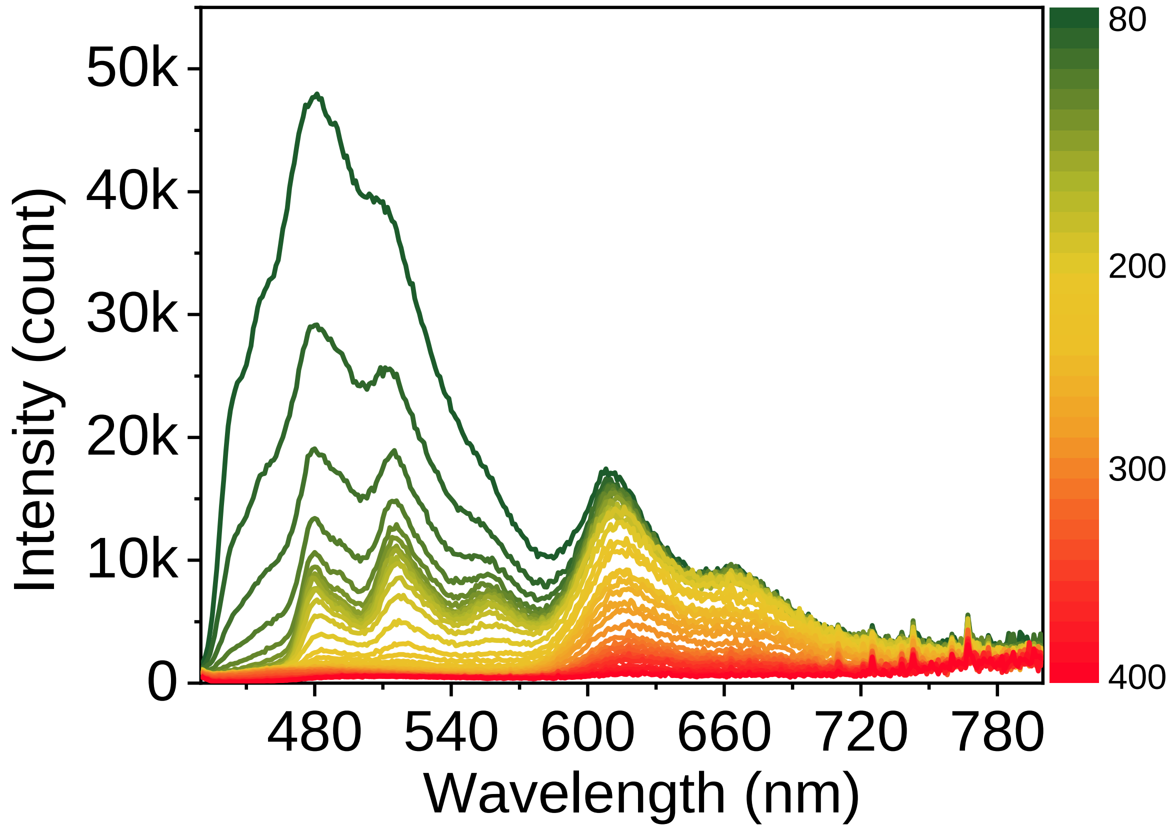

Supplementary Fig 18. Temperature-dependent delayed emission (Δt = 0.1 ms**,** TDDE) spectra of ***S*-4FMNNI@*R*-4FBrBI** (10 ppm, λ_{ex} = 424 nm), from 80 K to 400 K with **10 K interval**.

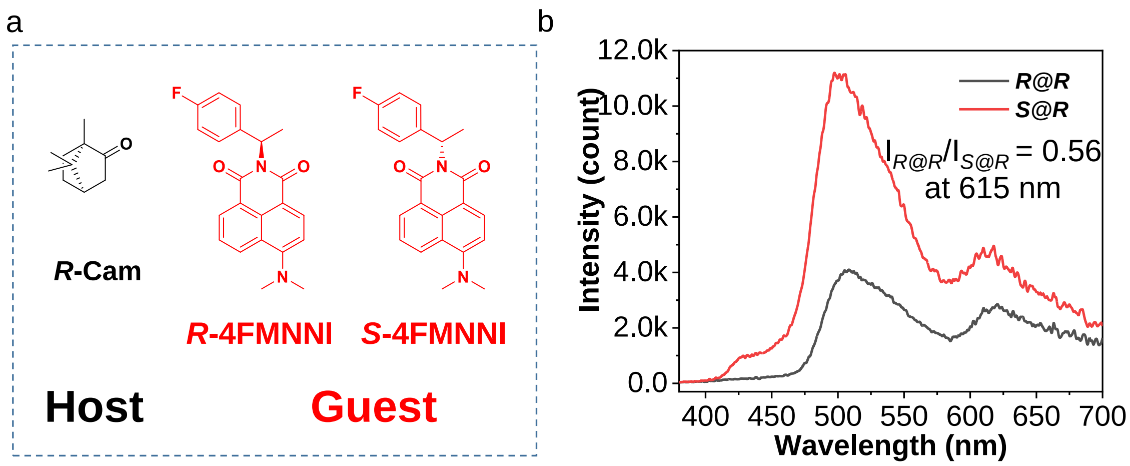

Supplementary Fig 19. a, Structures of host (***R*-Cam**: D(+)-camphor) and two chiral guests (***R*-4FMNNI, *S*-4FMNNI**) **b,** Delayed emission (DE, Δt =0.1 ms) spectra of two chiral guests (w/w 0.01%) in the ***R*-Cam** solid at 77 K (λ_{ex} = 365 nm).

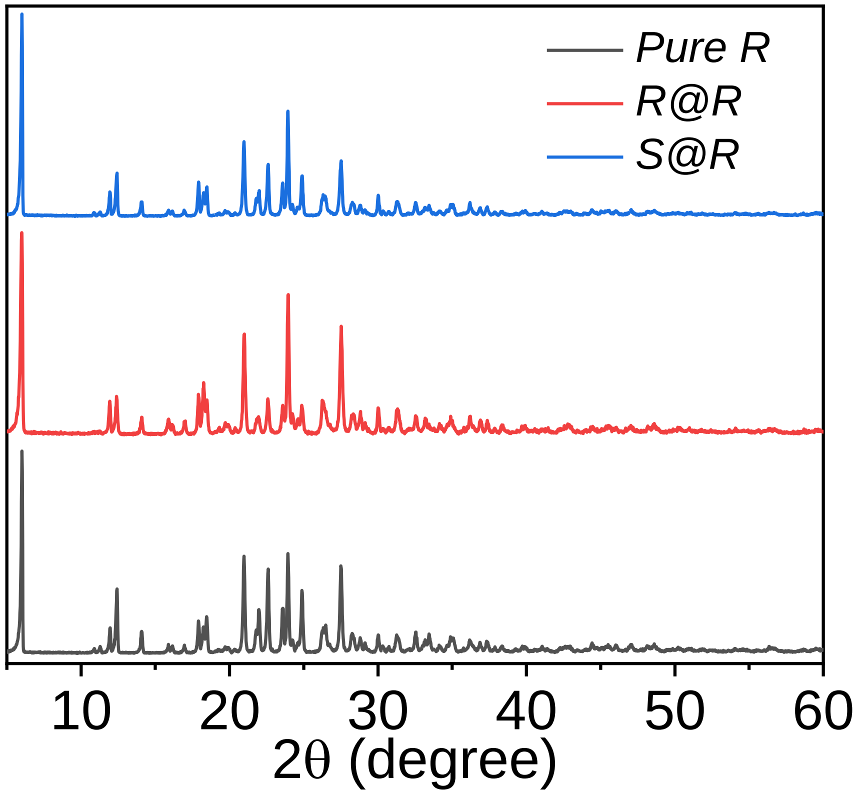

Supplementary Fig 20. X-ray diffraction patterns of the pristine solid powders of ***R*-4FBrBI**, ***R*-4FMNNI@*R*-4FBrBI** (***R*@*R***) and ***S*-4FMNNI@*R*-4FBrBI** (***S*@*R***) dopant solids (10 ppm).

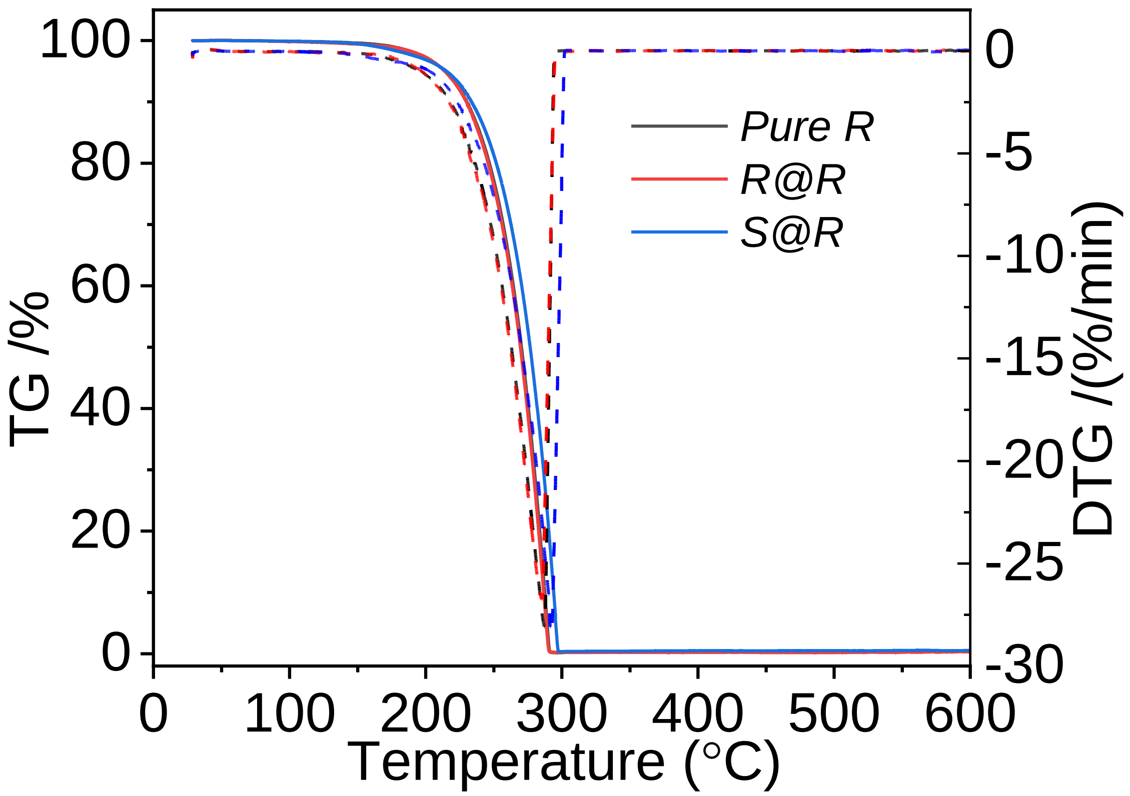

Supplementary Fig 21. TG (solid lines) and DTG (dash lines) of Pure ***R*-4FBrBI**, ***R*-4FMNNI@*R*-4FBrBI** (***R*@*R***) and ***S*-4FMNNI@*R*-4FBrBI** (***S*@*R***) dopant solids (10 ppm), from 30-600 °C (10 K/min) in N_2. Samples do not decompose before 110 °C, with a residual mass less than 0.4%.

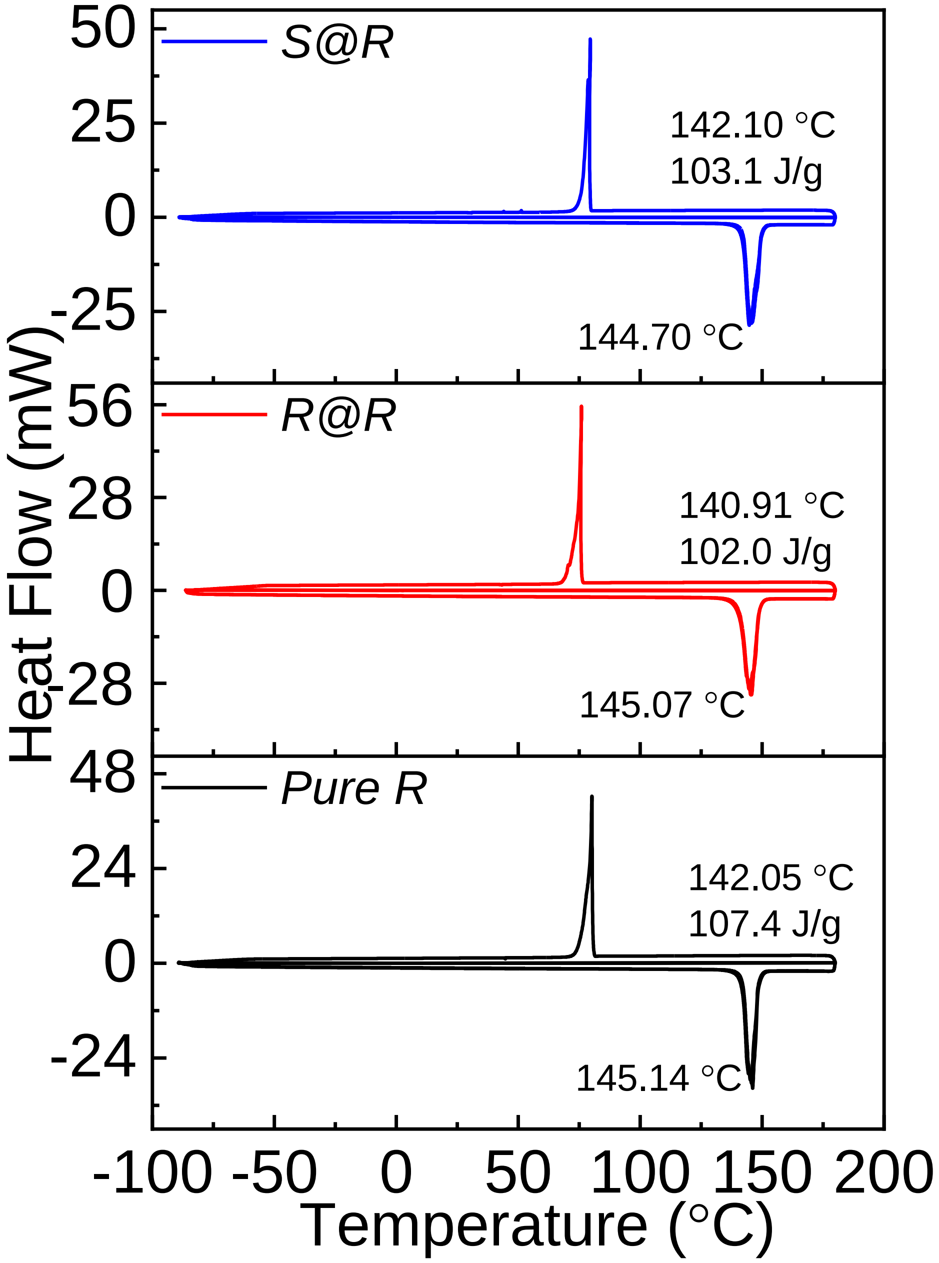

Supplementary Fig 22. DSC (differential scanning calorimetry) graphs of Pure ***R*-4FBrBI**, ***R*-4FMNNI@*R*-4FBrBI** (***R*@*R***) and ***S*-4FMNNI@*R*-4FBrBI** (***S*@*R***) dopant solids (10 ppm), from -90 to 180 °C at 10.0 °C/min.

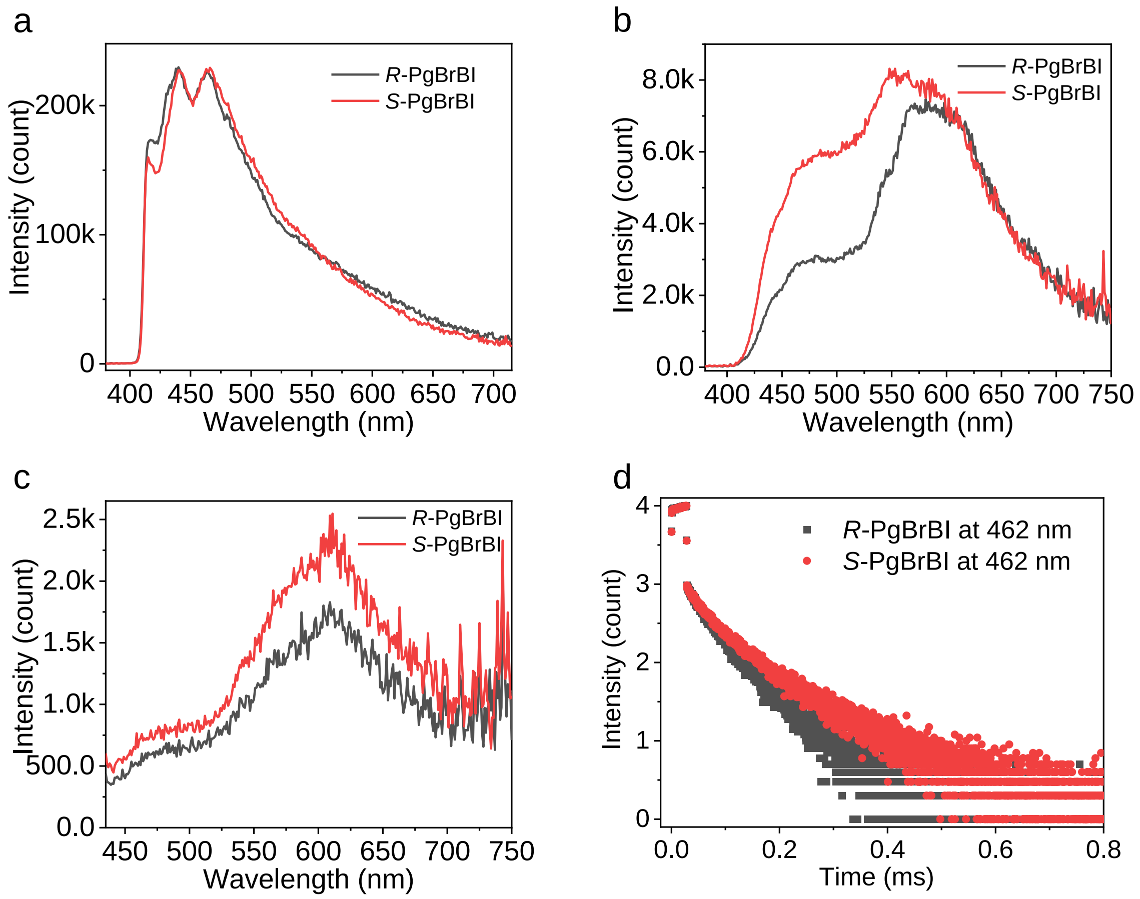

Supplementary Fig 23. a, PL spectra of pure ***R*-PgBrBI** and ***S*-PgBrBI** solid at 298 K (λ_{ex} = 365 nm). Delayed emission (DE, Δt = 0.1 ms) spectra of two host solids at 298 K with λ_{ex} = 365 nm for **b** and λ_{ex} = 424 nm for **c**. **d,** Time-resolved emission decay curves of pure ***R*-PgBrBI** and ***S*-PgBrBI** solid at 298 K (excited with spectraLED-370).

Supplementary Tab 3. Photoluminescence properties of dopant samples at (100 ppm w/w) and pure host solids at room temperature.

Samples[a]	λ_F (nm)[b]	τ_F (ns)[c]	λ_{RTP} (nm)[d]	τ_{RTP} (ms)[e]	ep[f]
R@R	518	3.00	621	63.36	11.5
S@R	521	2.80	623	40.34	
S@S	518	3.10	621	63.25	12.7
R@S	523	4.21	624	41.52	
***R*-PgBrBI**	/[g]	/	462	0.054	/
***S*-PgBrBI**	/	/	462	0.078	/

[a] Guest-host molecular solids (w/w 100 ppm). [b] Fluorescence emission maxima obtained from the steady-state photoluminescence spectrum excited at 365 nm. [c] Apparent fluorescence lifetime (naonoLED-370). [d] Phosphorescence emission maxima of delayed emission spectra excited at 424 nm. [e] Apparent phosphorescence lifetime (spectraLED-370). [f] Enantiomeric RTP enhancement ratios (ep, $I_{S@S}/I_{R@S}$ or $I_{R@R}/I_{S@R}$). [g] Signal too weak to measure or does not exist.

3. Crystal data

CCDC 2150413-2150416 contains the supplementary crystallographic data for this paper. These data are provided free of charge by The Cambridge Crystallographic Data Centre.

Supplementary Tab 4. Crystal data and structure refinement for ***R*-4FBrBI** and ***S*-4FBrBI**.

Empirical formula	$C_{16}H_{11}BrFNO_2$	$C_{16}H_{11}BrFNO_2$
	***R*-4FBrBI**	***S*-4FBrBI**
Formula weight	348.17	348.17
Temperature/K	299.82(11)	299.48(10)
Crystal system	monoclinic	monoclinic
Space group	$P2_1$	$P2_1$
a/Å	5.59960(10)	5.60057(5)
b/Å	29.8187(2)	29.8284(2)
c/Å	8.19040(10)	8.19092(6)
α/°	90	90
β/°	91.3480(10)	91.3294(7)
γ/°	90	90
Volume/Å^3	1367.20(3)	1367.977(19)
Z	4	4
ρ_{calc}g/cm^3	1.691	1.691
μ/mm^{-1}	4.249	4.247
F(000)	696.0	696.0
Crystal size/mm^3	0.04 × 0.04 × 0.02	0.2 × 0.2 × 0.2
Radiation	CuKα (λ = 1.54184)	CuKα (λ = 1.54184)
2Θ range for data collection/°	10.804 to 158.878	10.804 to 158.69
Index ranges	-7 ≤ h ≤ 7, -37 ≤ k ≤ 37, -9 ≤ l ≤ 7	-3 ≤ h ≤ 6, -37 ≤ k ≤ 37, -10 ≤ l ≤ 10
Reflections collected	15320	8858
Independent reflections	5708 [R_{int} = 0.0268, R_{sigma} = 0.0260]	5025 [R_{int} = 0.0200, R_{sigma} = 0.0273]
Data/restraints/parameters	5708/1/381	5025/1/381
Goodness-of-fit on F^2	1.024	1.059
Final R indexes [I>=2σ (I)]	R_1 = 0.0265, wR_2 = 0.676	R_1 = 0.0259, wR_2 = 0.0676
Final R indexes [all data]	R_1 = 0.0282, wR_2 = 0.0684	R_1 = 0.0262, wR_2 = 0.0678
Largest diff. peak/hole/e Å^{-3}	0.24/-0.33	0.31/-0.40
Flack parameter	-0.035(8)	-0.033(8)

Supplementary Tab 5. Crystal data and structure refinement for ***R*-4FMNNI** and ***S*-4FMNNI**.

Empirical formula	$C_{22}H_{19}FN_2O_2$	$C_{22}H_{19}FN_2O_2$
	***R*-4FMNNI**	***S*-4FMNNI**
Formula weight	362.39	362.39
Temperature/K	300.41(10)	299.39(10)
Crystal system	monoclinic	monoclinic
Space group	$P2_1$	$P2_1$
a/Å	11.2514(3)	11.2533(3)
b/Å	5.62150(10)	5.62290(10)
c/Å	14.8899(3)	14.8948(4)
α/°	90	90
β/°	108.130(2)	108.129(3)
γ/°	90	90
Volume/Å^3	895.03(4)	895.70(4)
Z	2	2
ρ_{calc}g/cm^3	1.345	1.344
μ/mm^{-1}	0.769	0.769
F(000)	380.0	380.0
Crystal size/mm^3	0.4 × 0.4 × 0.4	0.2 × 0.2 × 0.2
Radiation	CuKα (λ = 1.54184)	CuKα (λ = 1.54184)
2Θ range for data collection/°	8.268 to 158.376	8.268 to 158.194
Index ranges	-13 ≤ h ≤ 11, -7 ≤ k ≤ 6, -18 ≤ l ≤ 18	-13 ≤ h ≤ 14, -7 ≤ k ≤ 7, -18 ≤ l ≤ 18
Reflections collected	9857	9610
Independent reflections	3477 [R_{int} = 0.0287, R_{sigma} = 0.0243]	3516 [R_{int} = 0.0459, R_{sigma} = 0.0396]
Data/restraints/parameters	3477/1/247	3516/1/247
Goodness-of-fit on F^2	1.069	1.078
Final R indexes [I>=2σ (I)]	R_1 = 0.0512, wR_2 = 0.1392	R_1 = 0.0590, wR_2 = 0.1679
Final R indexes [all data]	R_1 = 0.0549, wR_2 = 0.1413	R_1 = 0.0643, wR_2 = 0.1753
Largest diff. peak/hole/e Å^{-3}	0.36/-0.25	0.39/-0.27
Flack parameter	-0.01(9)	-0.1(2)

Supplementary Tab 6. Crystal data and *sp*^{3} angle of model compound

	crystal system	space group	α/°	β/°	γ/°	*sp*^{3} angle/°
***R*-4FBrBI**	monoclinic	$P2_1$	90	91.35	90	109.28
***S*-4FBrBI**	monoclinic	$P2_1$	90	91.33	90	109.52
***R*-4FMNNI**	monoclinic	$P2_1$	90	108.13	90	113.32
***S*-4FMNNI**	monoclinic	$P2_1$	90	108.13	90	113.08

4. Lifetime measurements

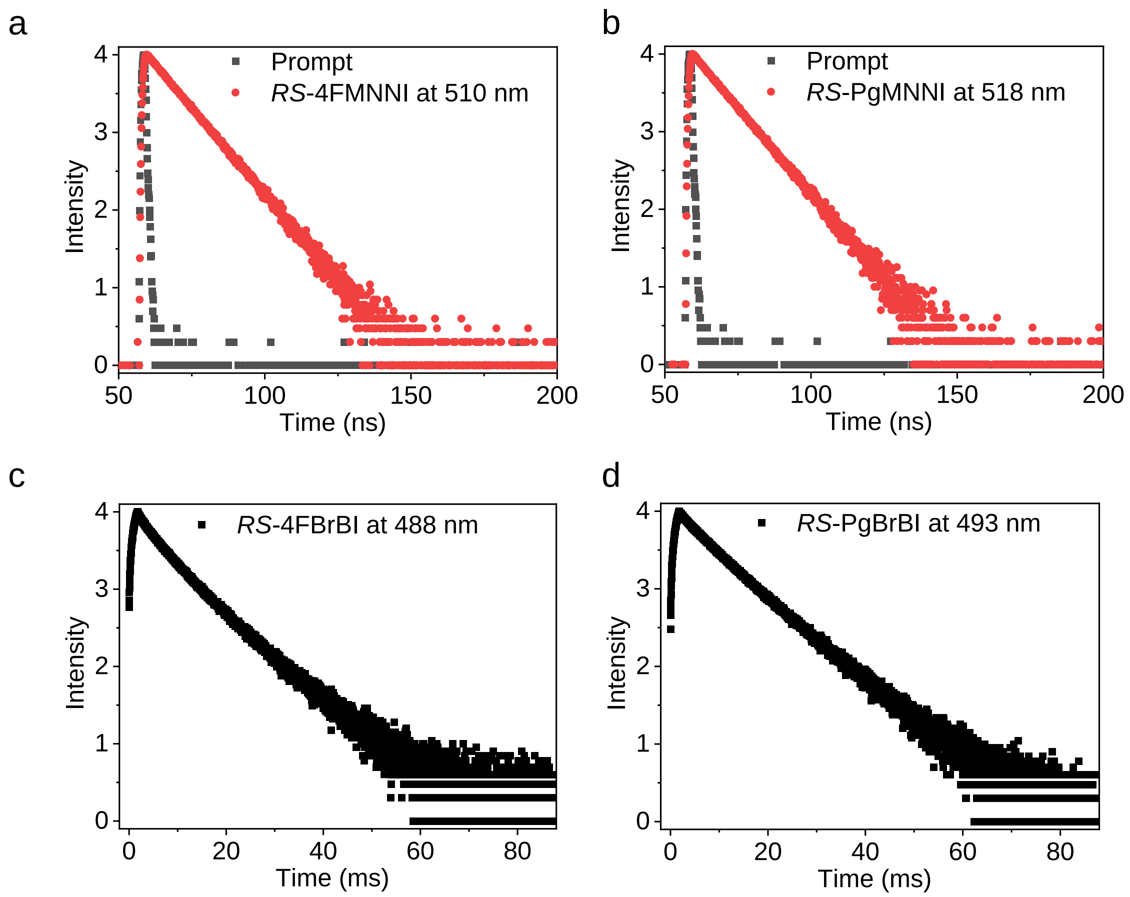

Supplementary Fig 24. Emission decay curves of ***RS*-4FMNNI a** and ***RS*-PgMNNI b** in DCM ($2x10^{-5}$ M) at 298 K (excited with nanoLED-370). Emission decay curves of ***RS*-4FBrBI c** and ***RS*-PgBrBI d** in DCM at 77 K (excited with spectraLED-370).

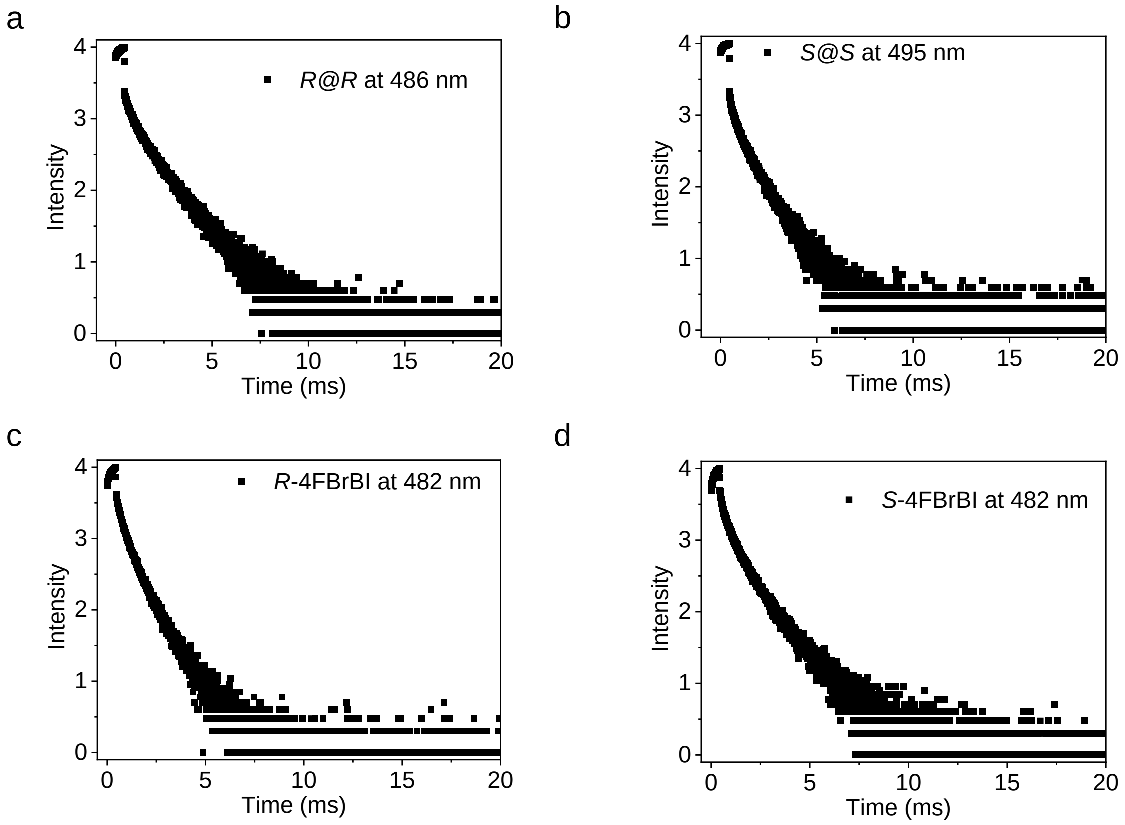

Supplementary Fig 25. Time-resolved emission decay curves of doping samples (**a** for ***R*@*R*** and **b** for ***S*@*S***) and pure host solid (**c** for ***R*-4FBrBI** and **d** for ***S*-4FBrBI**) at 298 K (excited with spectraLED-370).

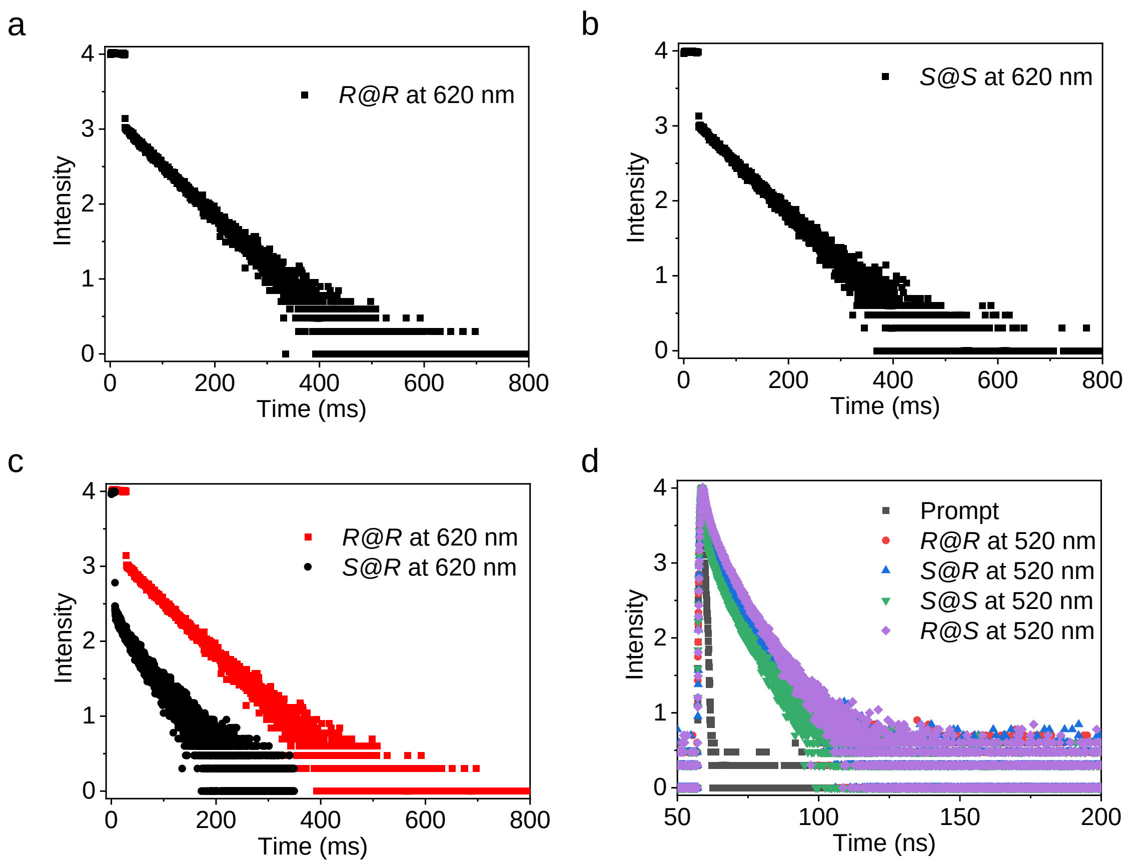

Supplementary Fig 26. a, b, c, Time-resolved emission decay curves of 100 ppm ***R@R***, ***S@S***, ***S@R*** solid (**Pg series**) at 298 K (excited with spectraLED-370). **d,** Emission decay curves at 298 K (excited with nanoLED-370).

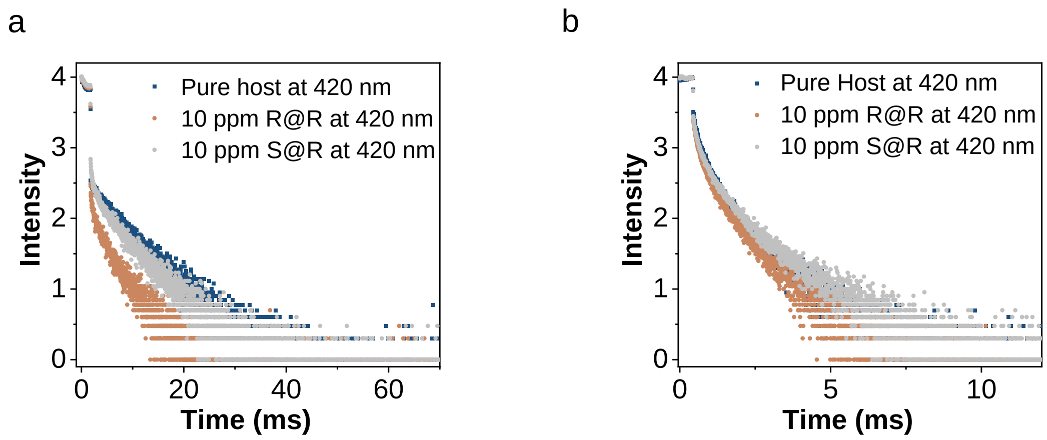

Supplementary Fig 27. Time-resolved emission decay curves of Pure Host (***R*-4FBrBI**), 10 ppm ***R@R*** and 10 ppm ***S@R*** at 77 K (**a**) and at room temperature (**b**) when monitored at 420 nm and excited with spectraLED-340.

Supplementary Tab 7. Photoluminescence properties of dopant samples at (10 ppm w/w) and pure host solids at 77 K and room temperature when monitored at 420 nm.

Temperature	77 K			Room Temperature		
Samples[a]	τ (ms)[b]		Weighted average lifetime (ms)	τ (ms)[c]		Weighted average lifetime (ms)
***R*-4FBrBI**	5.407	100%	5.407	0.088<br>0.427<br>1.367	13.67%<br>47.16%<br>39.16%	0.749
S@R	0.548<br>5.103	14.46%<br>85.54%	4.444	0.081<br>0.363<br>1.073	11.65%<br>49.43%<br>38.92%	0.606
R@R	0.408<br>3.463	13.91%<br>86.09%	3.038	0.077<br>0.304<br>0.961	13.40%<br>41.44%<br>45.16%	0.570

[a] Guest-host molecular solids (w/w 10 ppm). [b] Phosphorescence lifetime at 77 K when monitored at 420 nm and excited with spectraLED-340. [c] Phosphorescence lifetime at room temperature when monitored at 420 nm and excited with spectraLED-340.

5. EPR Spectra

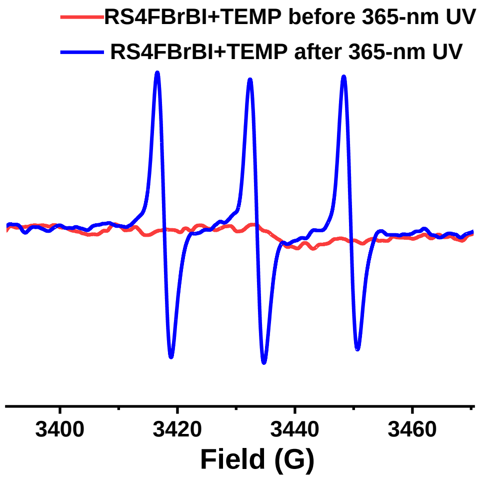

Supplementary Fig 28. EPR spectra of the host molecule **RS4FBrBI** in the presence of a singlet-oxygen capture agent 2,2,6,6-Tetramethylpiperidine (TEMP) before and after exposure to a broad-band 365-nm LED lamp (340-390 nm).

6. Solid-state Absorption

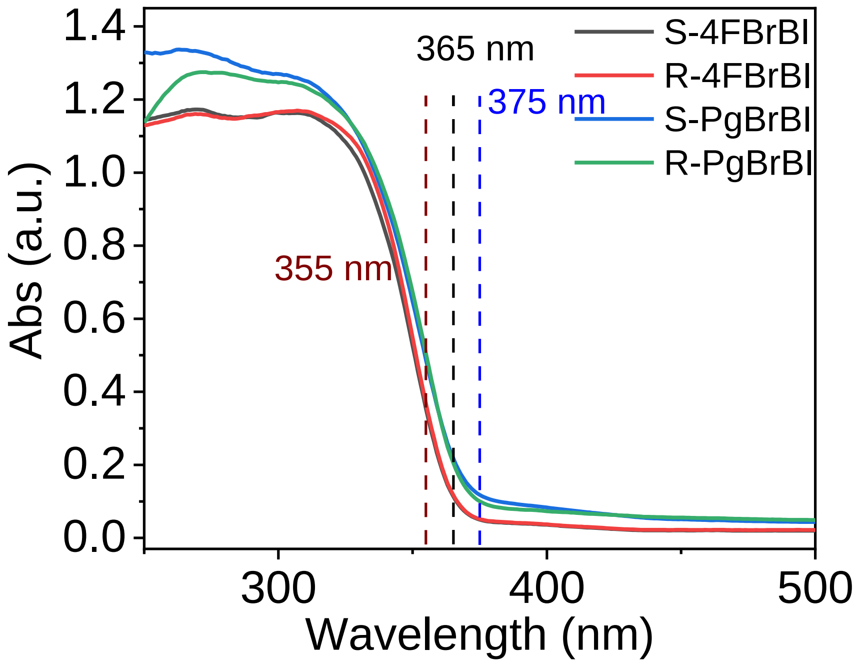

Supplementary Fig 29. Solid-state absorption spectra of the host molecules showing that they can be excited by beams near 365 nm.

7. Nuclear magnetic resonance (NMR) and high-resolution mass spectra (HRMS)

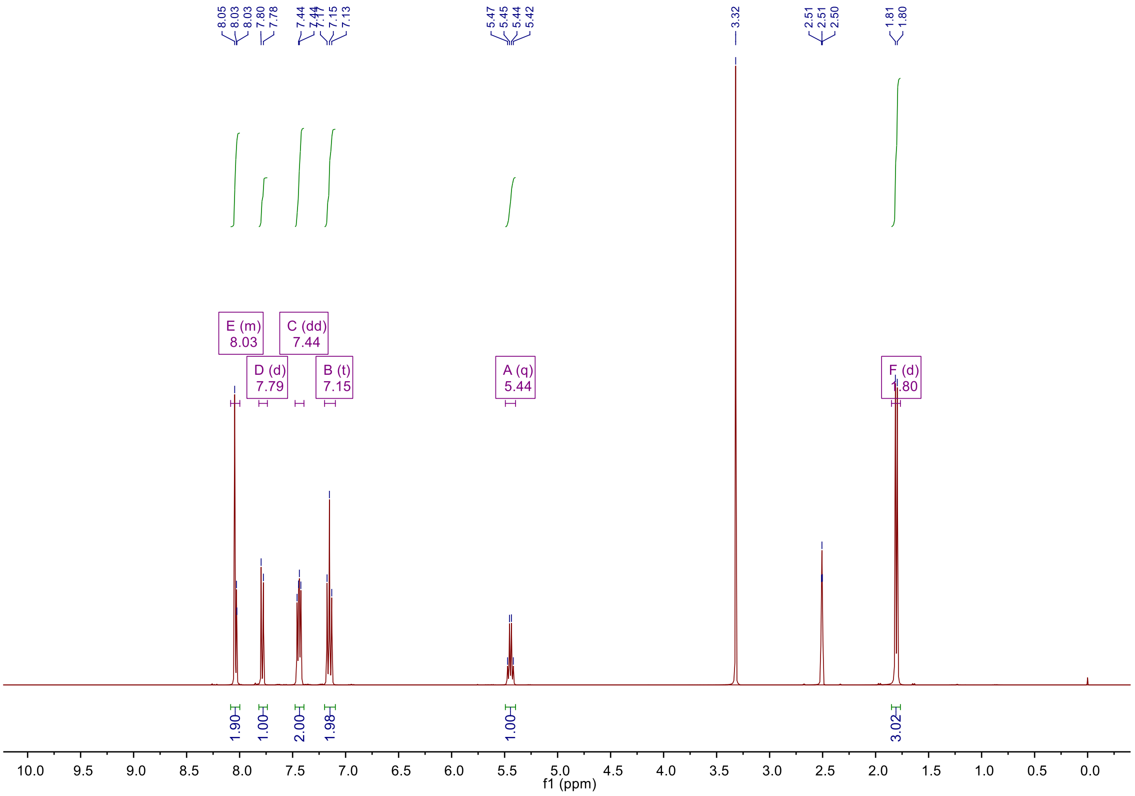

Supplementary Fig 30. ^{1}H NMR spectrum of ***RS*-4FBrBI** in d-DMSO.

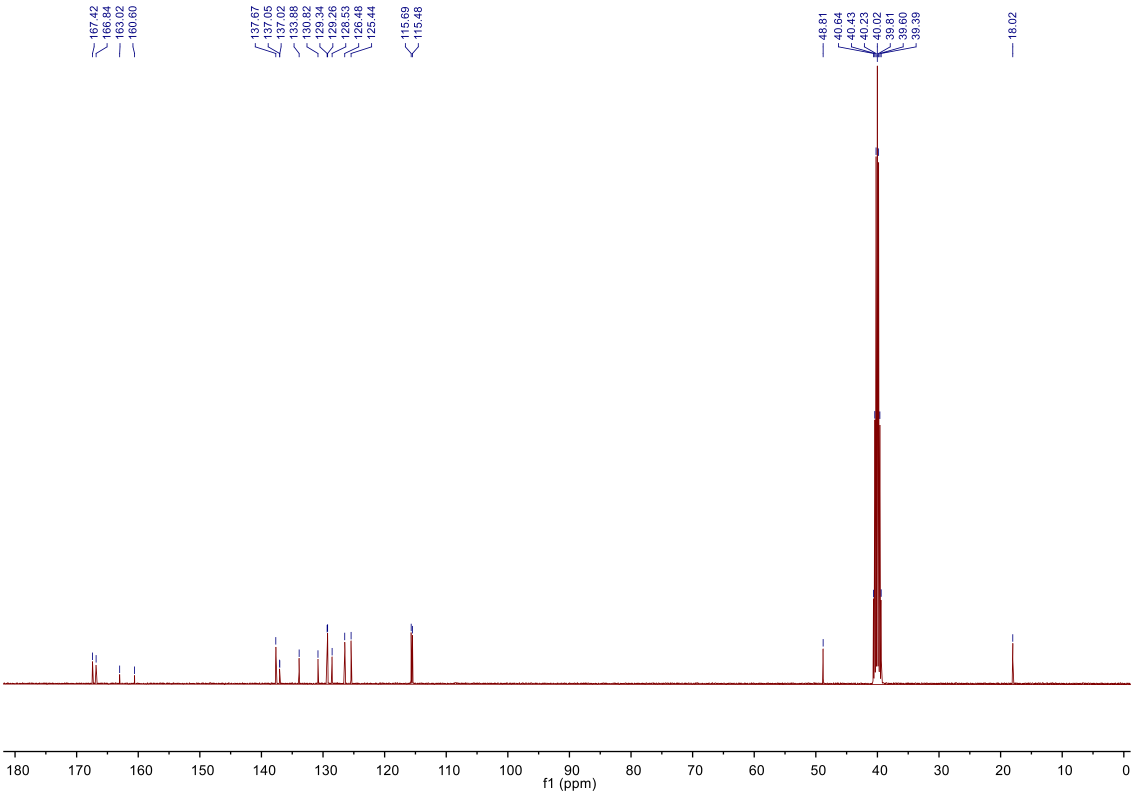

Supplementary Fig 31. ^{13}C NMR spectrum of ***RS*-4FBrBI** in d-DMSO.

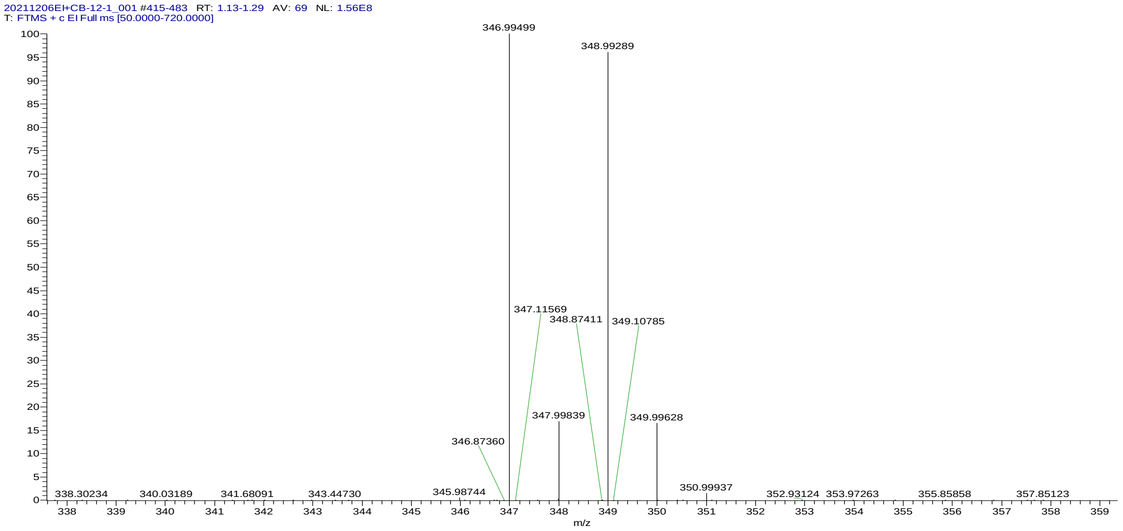

Supplementary Fig 32. EI mass spectrum of ***RS*-4FBrBI**.

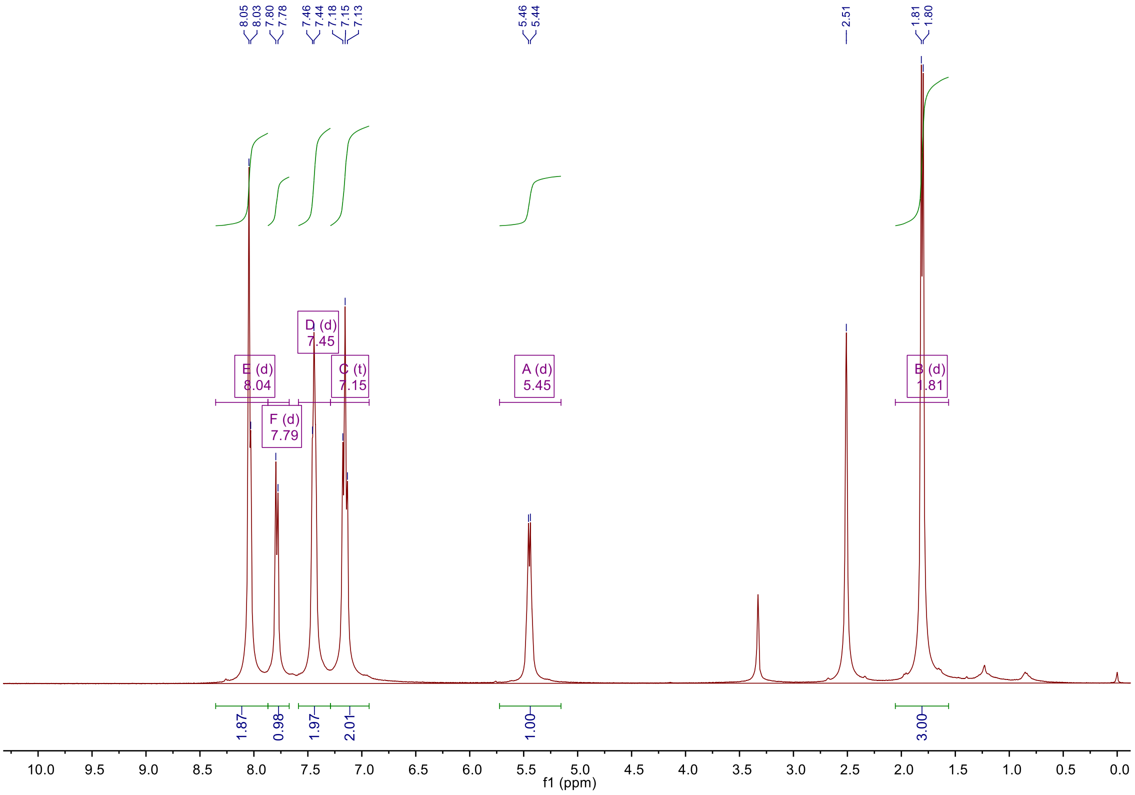

Supplementary Fig 33. ^{1}H NMR spectrum of ***R*-4FBrBI** in d-DMSO.

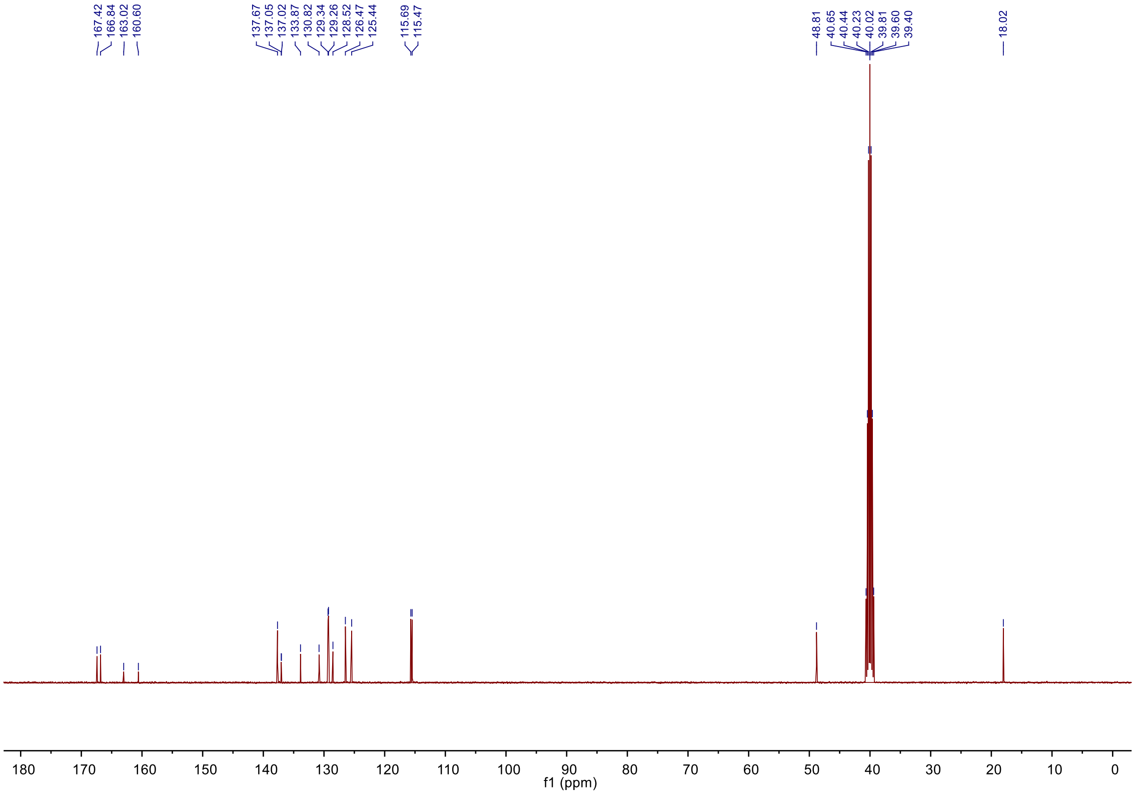

Supplementary Fig 34. ^{13}C NMR spectrum of ***R*-4FBrBI** in d-DMSO.

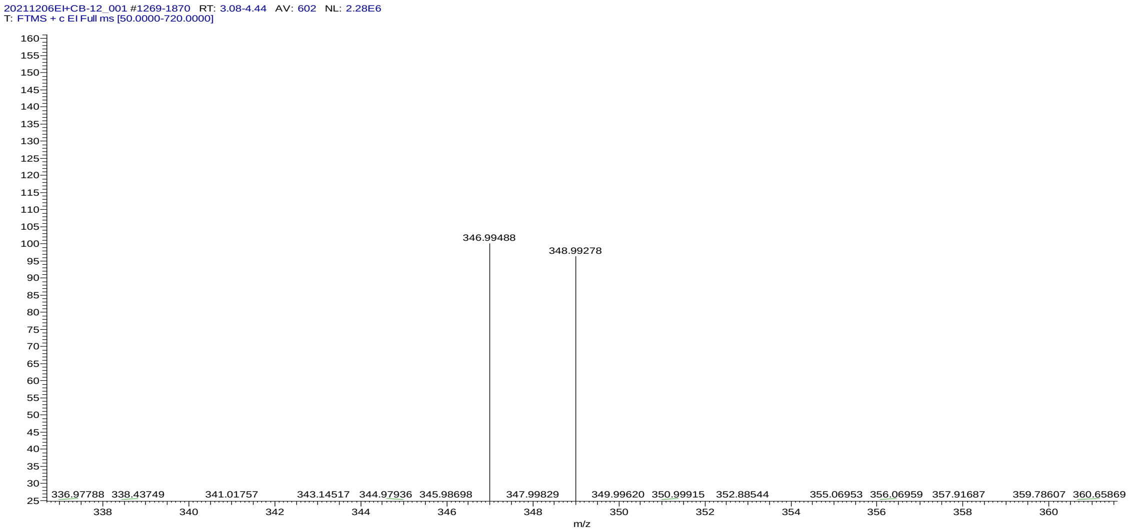

Supplementary Fig 35. EI mass spectrum of ***R*-4FBrBI**.

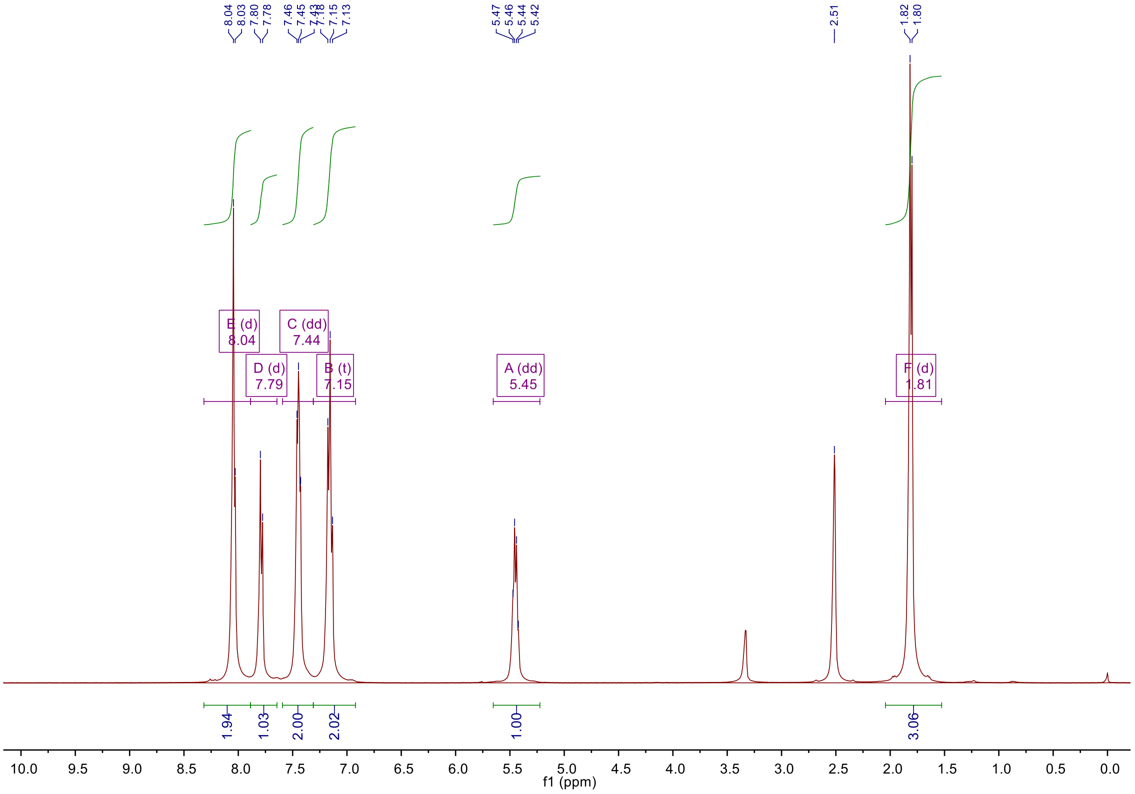

Supplementary Fig 36. ^{1}H NMR spectrum of ***S*-4FBrBI** in d-DMSO.

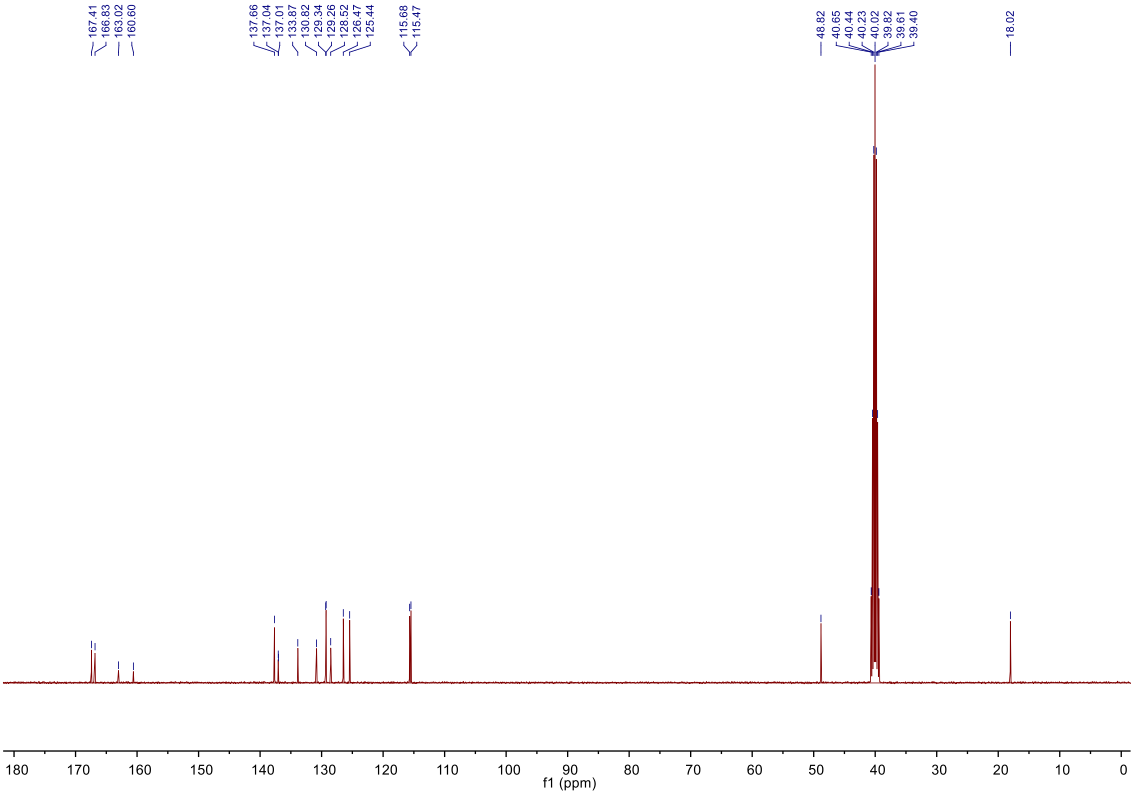

Supplementary Fig 37. ^{13}C NMR spectrum of ***S*-4FBrBI** in d-DMSO.

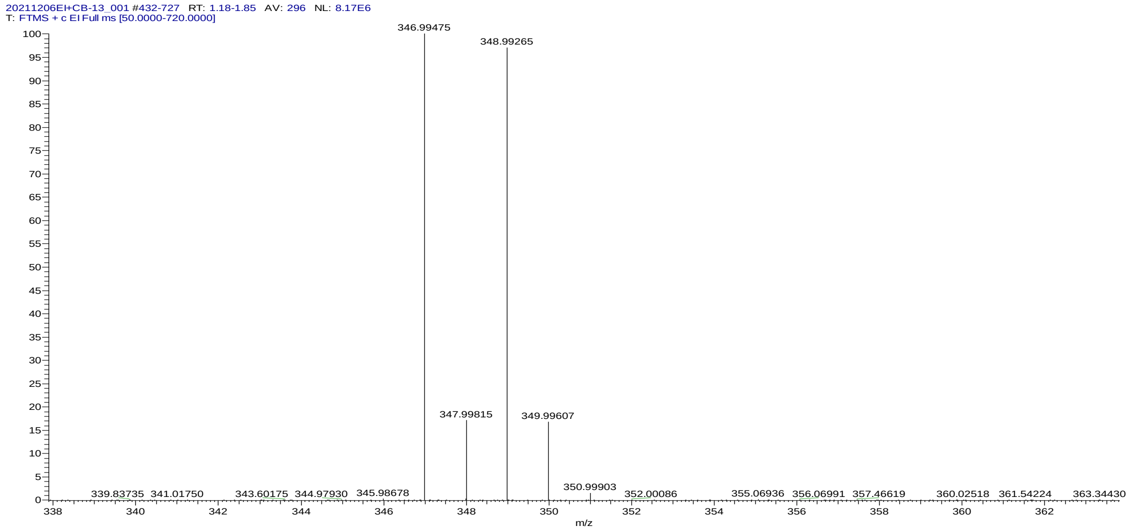

Supplementary Fig 38. EI mass spectrum of ***S*-4FBrBI**.

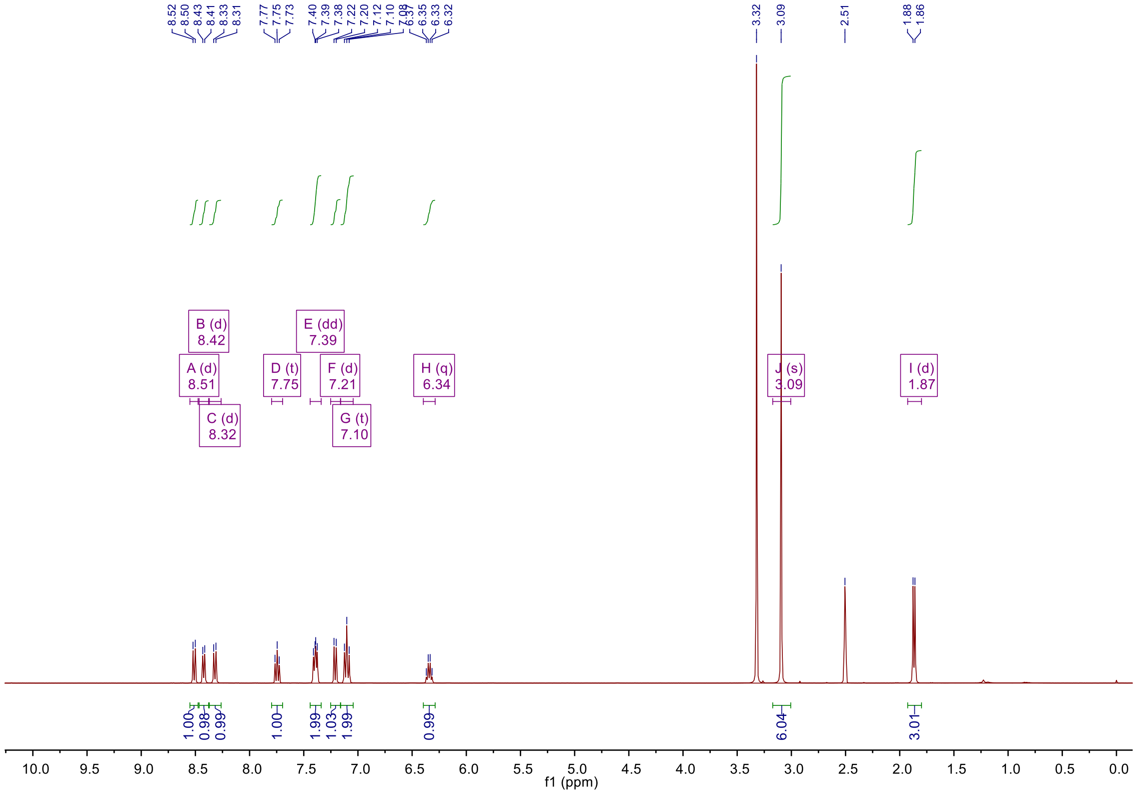

Supplementary Fig 39. ^{1}H NMR spectrum of ***RS*-4FMNNI** in d-DMSO.

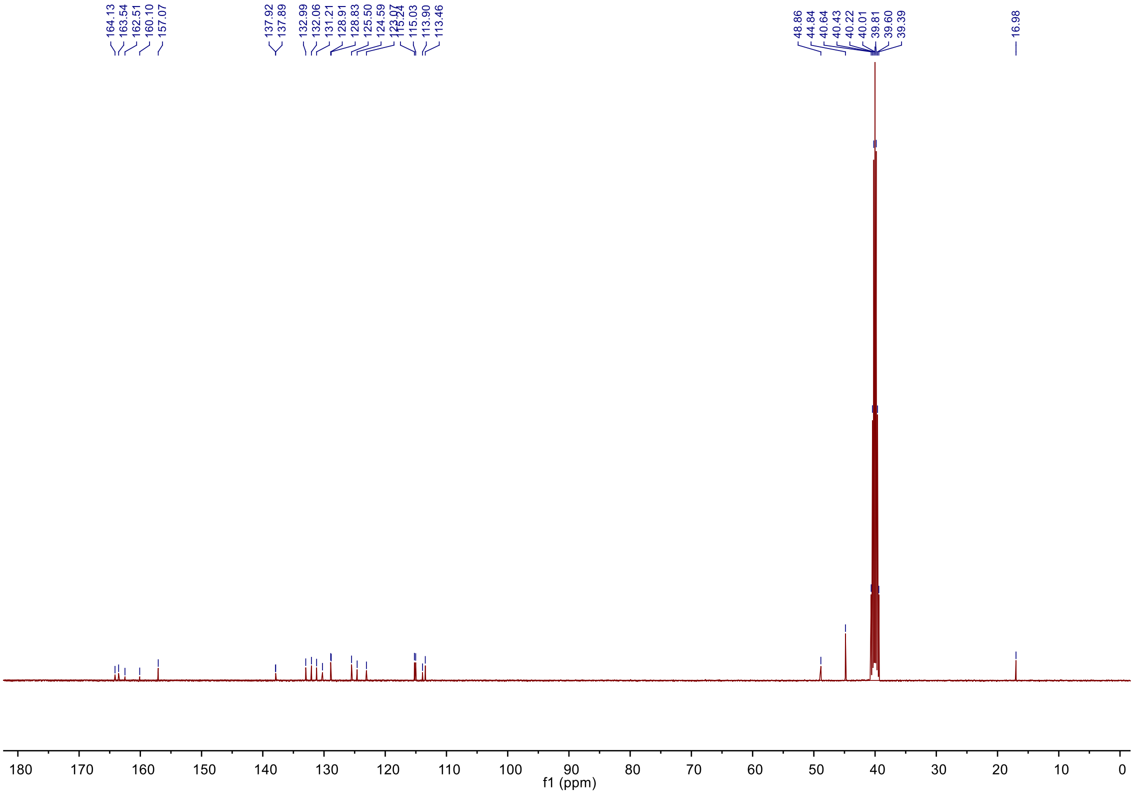

Supplementary Fig 40. ^{13}C NMR spectrum of ***RS*-4FMNNI** in d-DMSO.

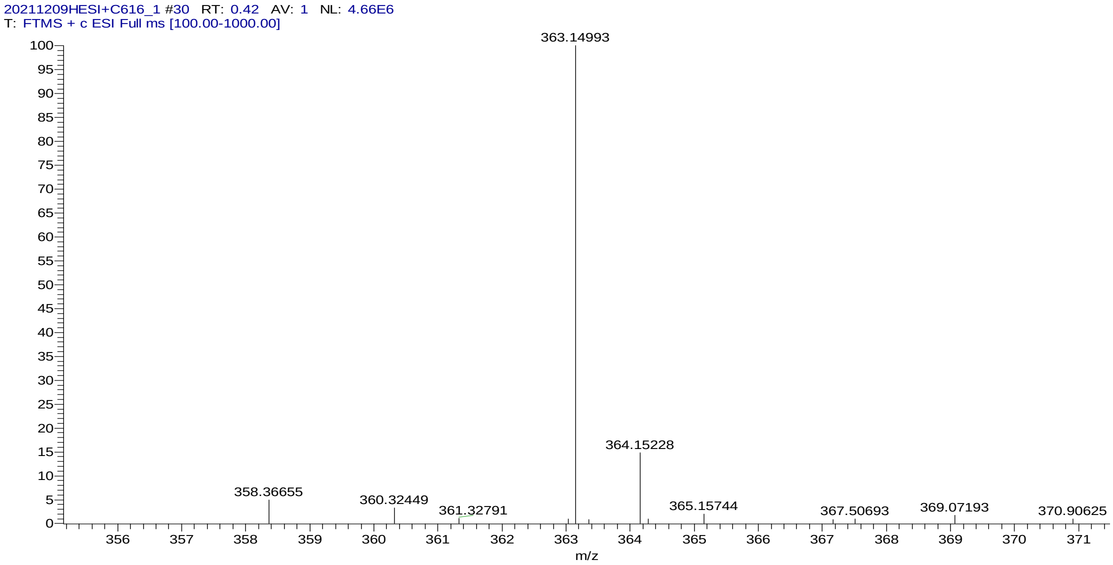

Supplementary Fig 41. ESI mass spectrum of ***RS*-4FMNNI**.

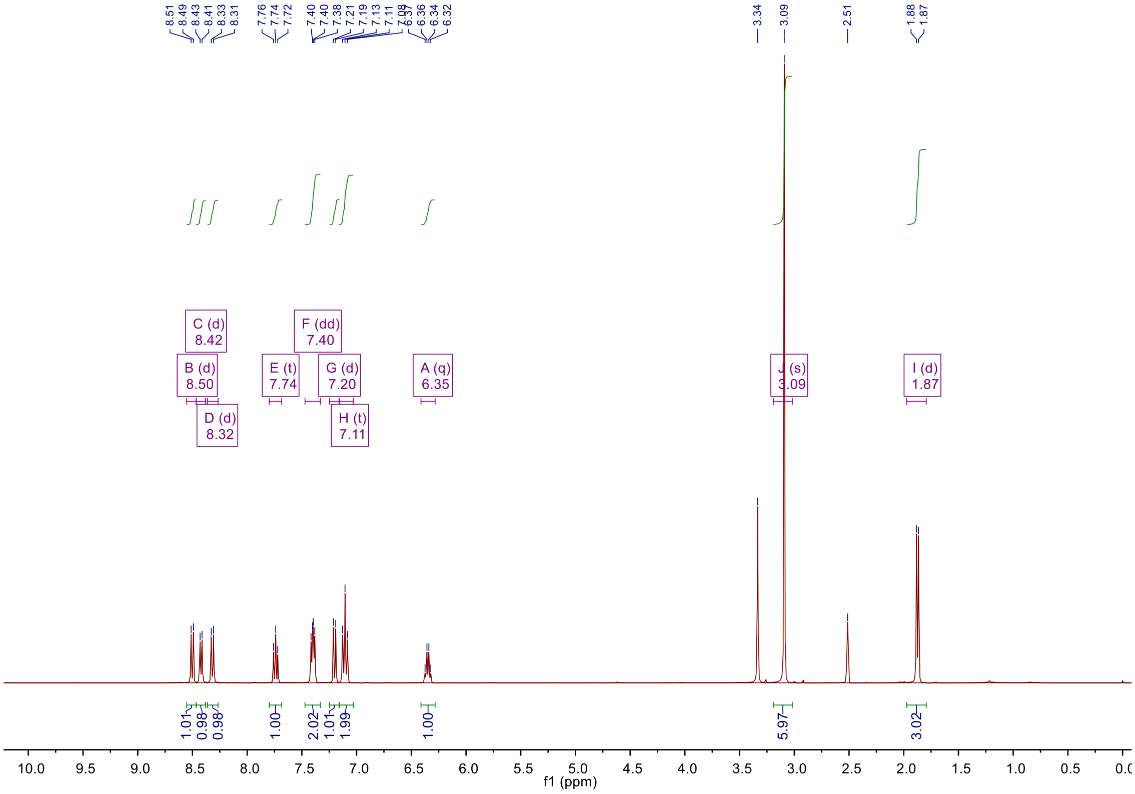

Supplementary Fig 42. ^{1}H NMR spectrum of ***R*-4FMNNI** in d-DMSO.

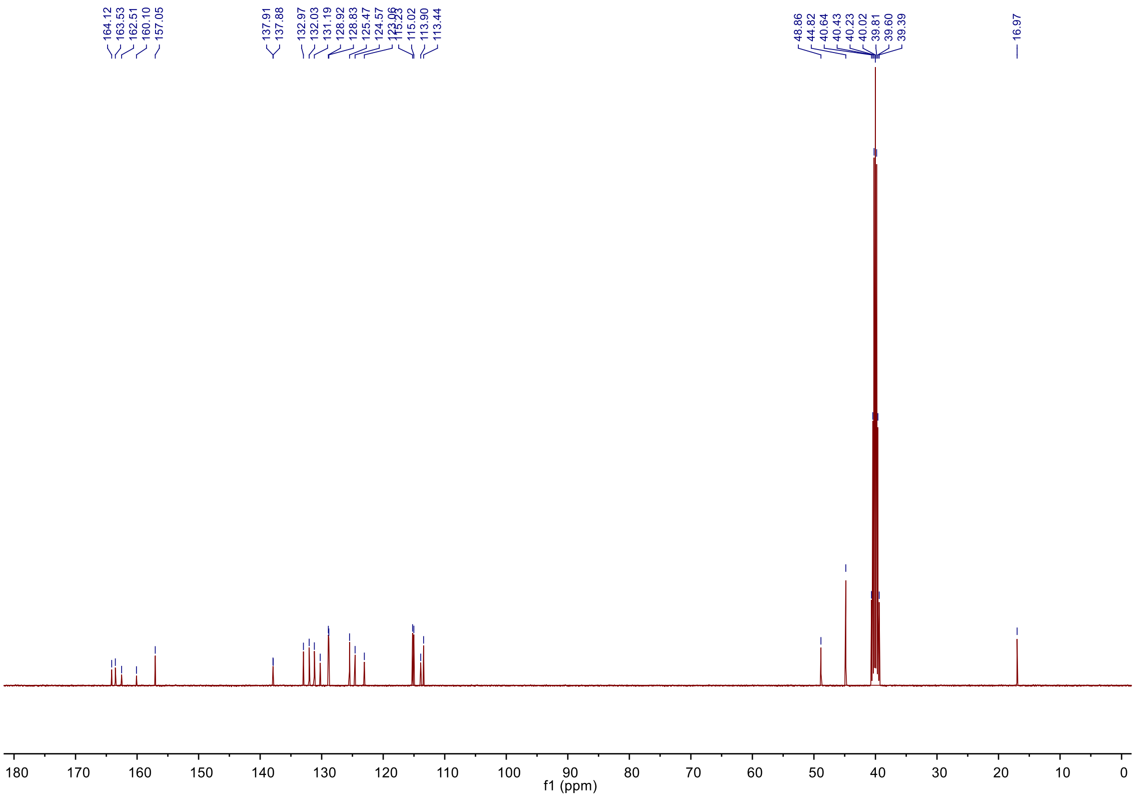

Supplementary Fig 43. ^{13}C NMR spectrum of ***R*-4FMNNI** in d-DMSO.

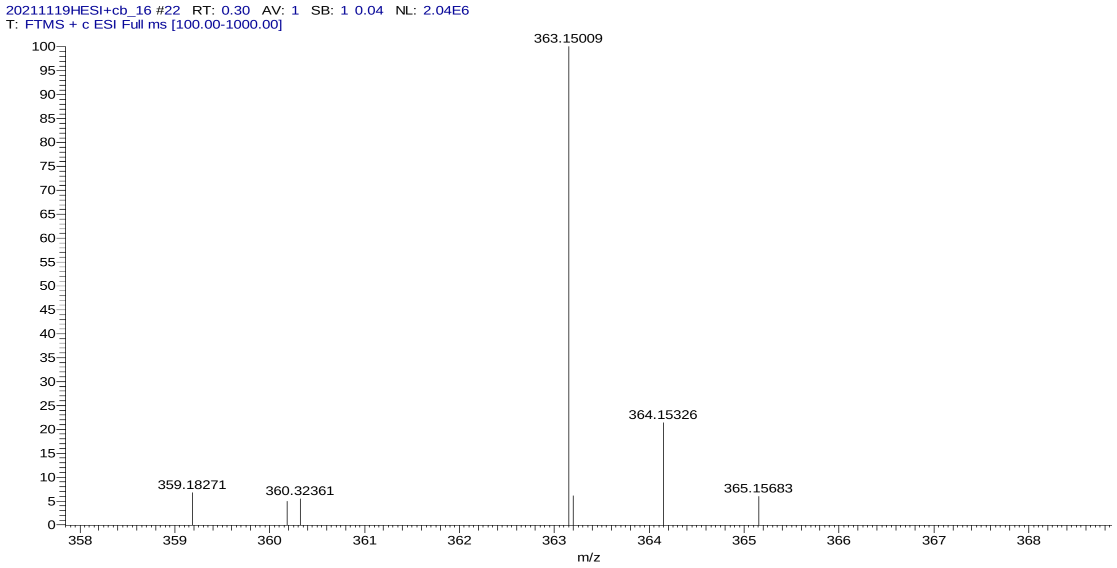

Supplementary Fig 44. ESI mass spectrum of ***R*-4FMNNI**.

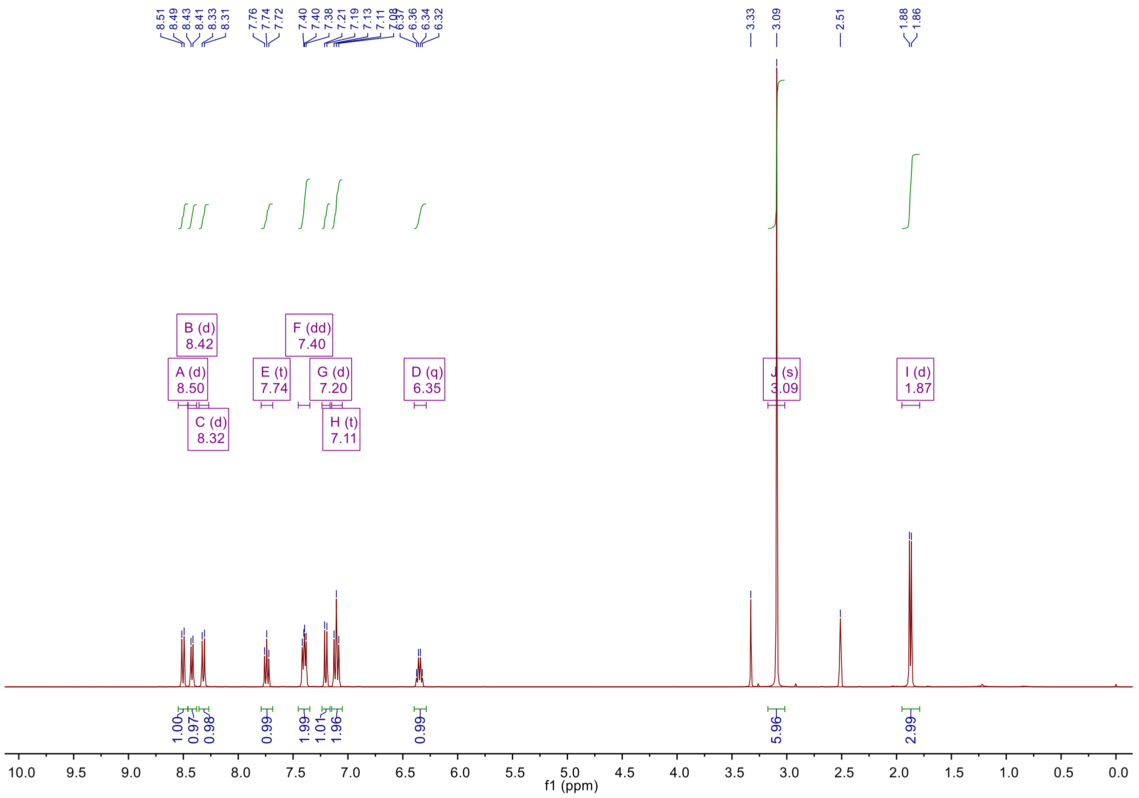

Supplementary Fig 45. ^{1}H NMR spectrum of ***S*-4FMNNI** in d-DMSO.

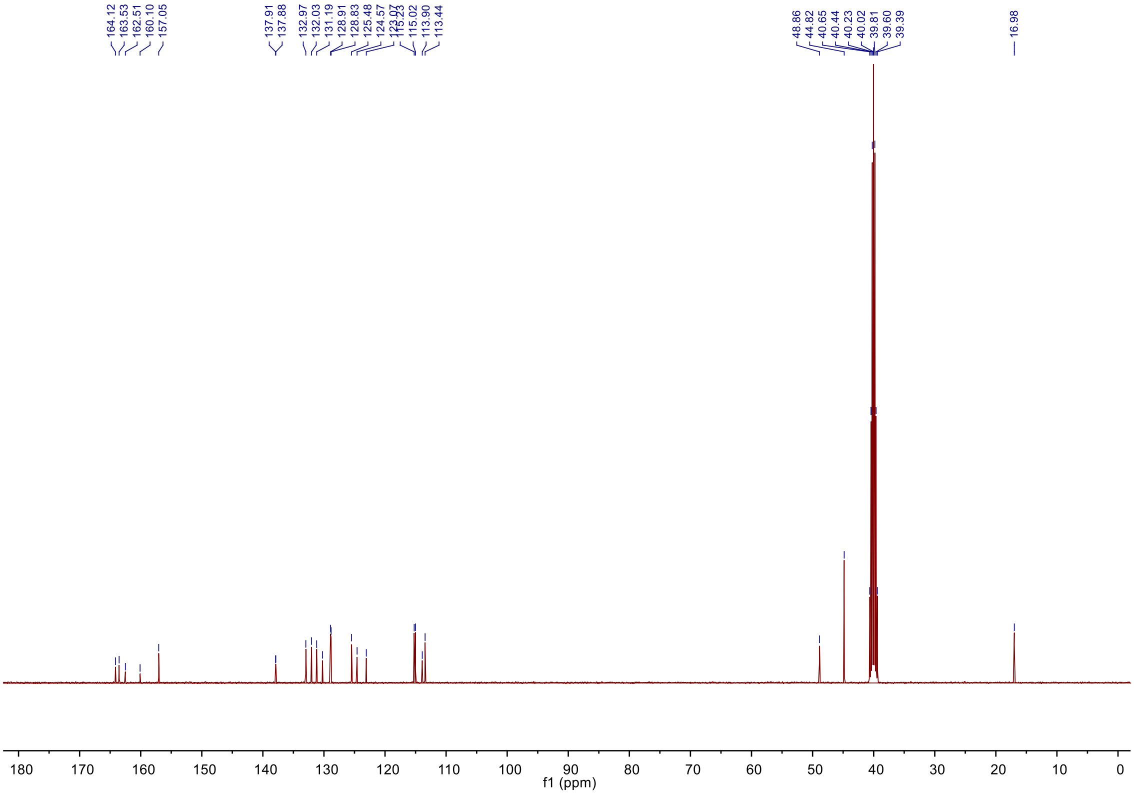

Supplementary Fig46. ^{13}C NMR spectrum of ***S*-4FMNNI** in d-DMSO.

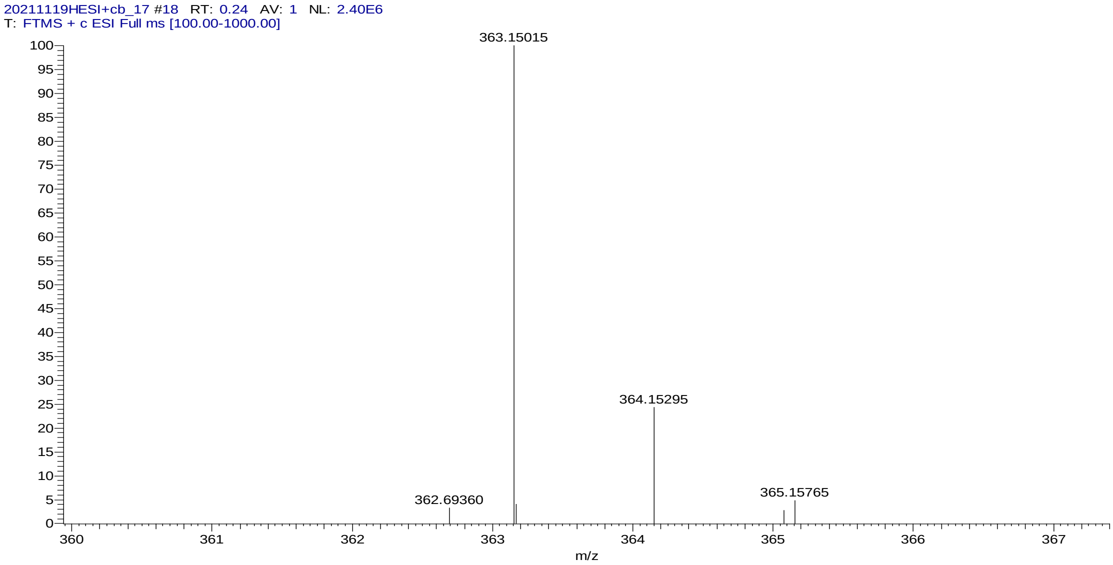

Supplementary Fig47. ESI mass spectrum of ***S*-4FMNNI**.

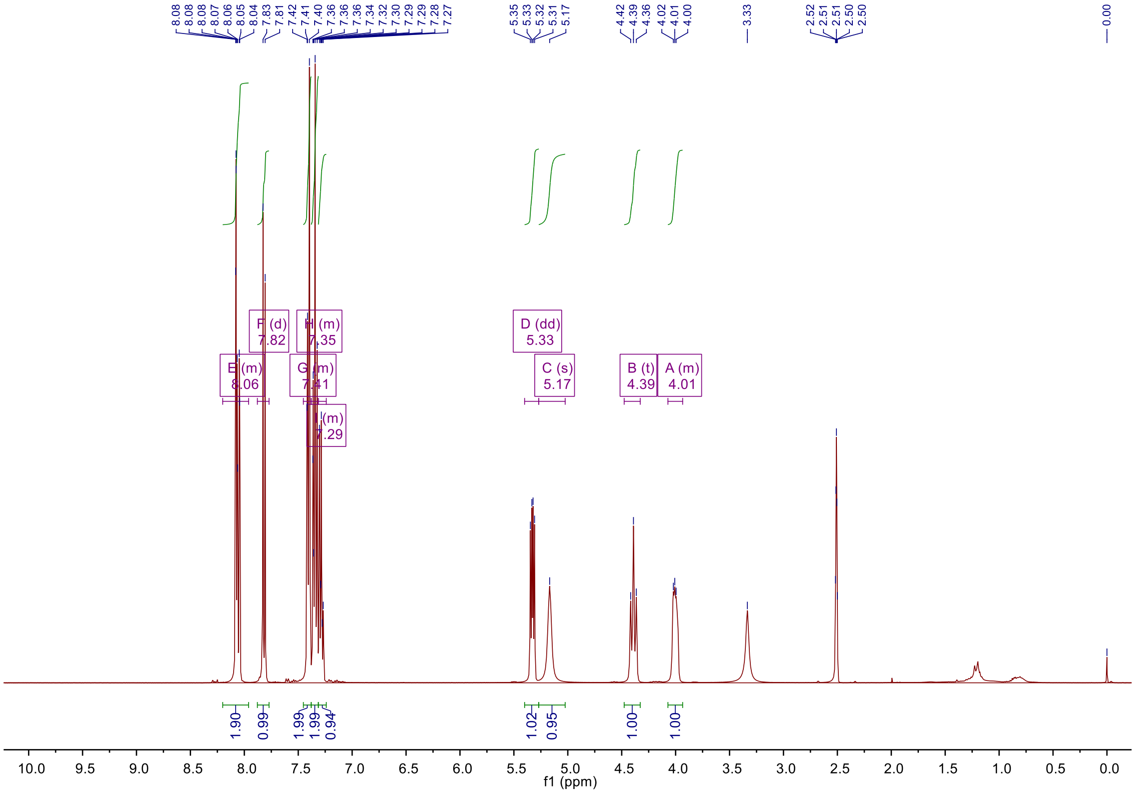

Supplementary Fig48. ^{1}H NMR spectrum of ***RS*-PgBrBI** in d-DMSO.

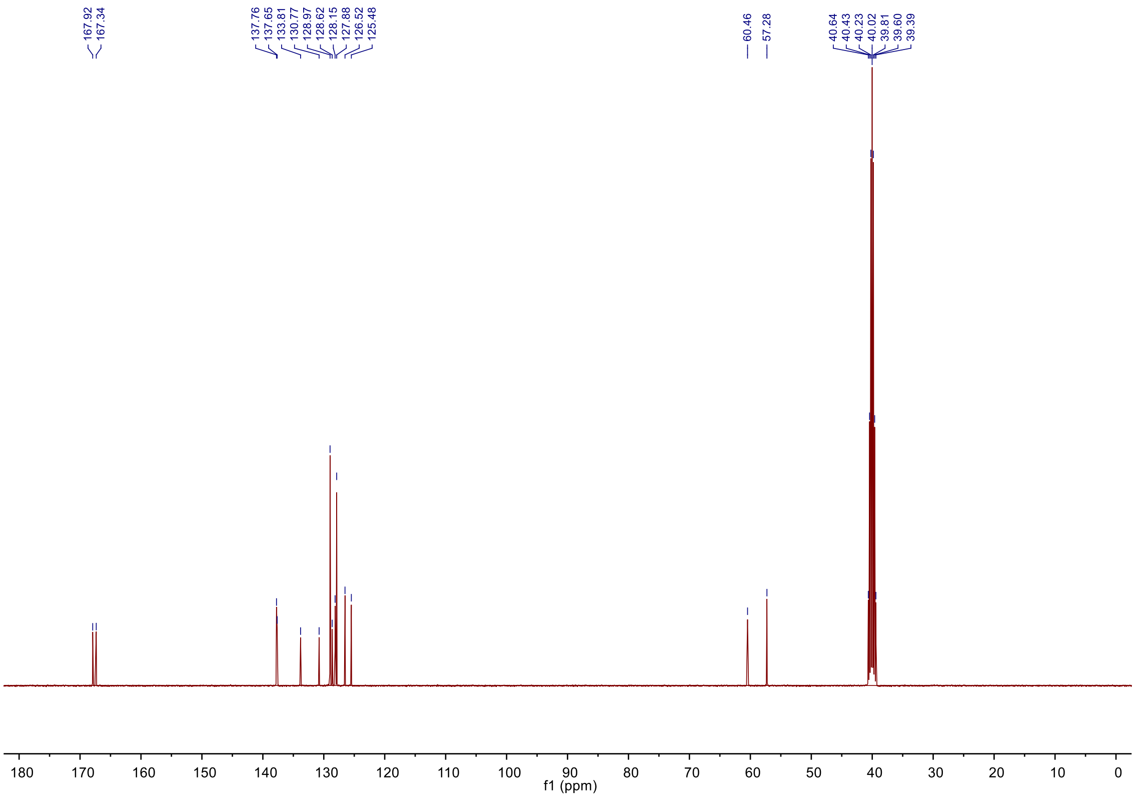

Supplementary Fig49. ^{13}C NMR spectrum of ***RS*-PgBrBI** in d-DMSO.

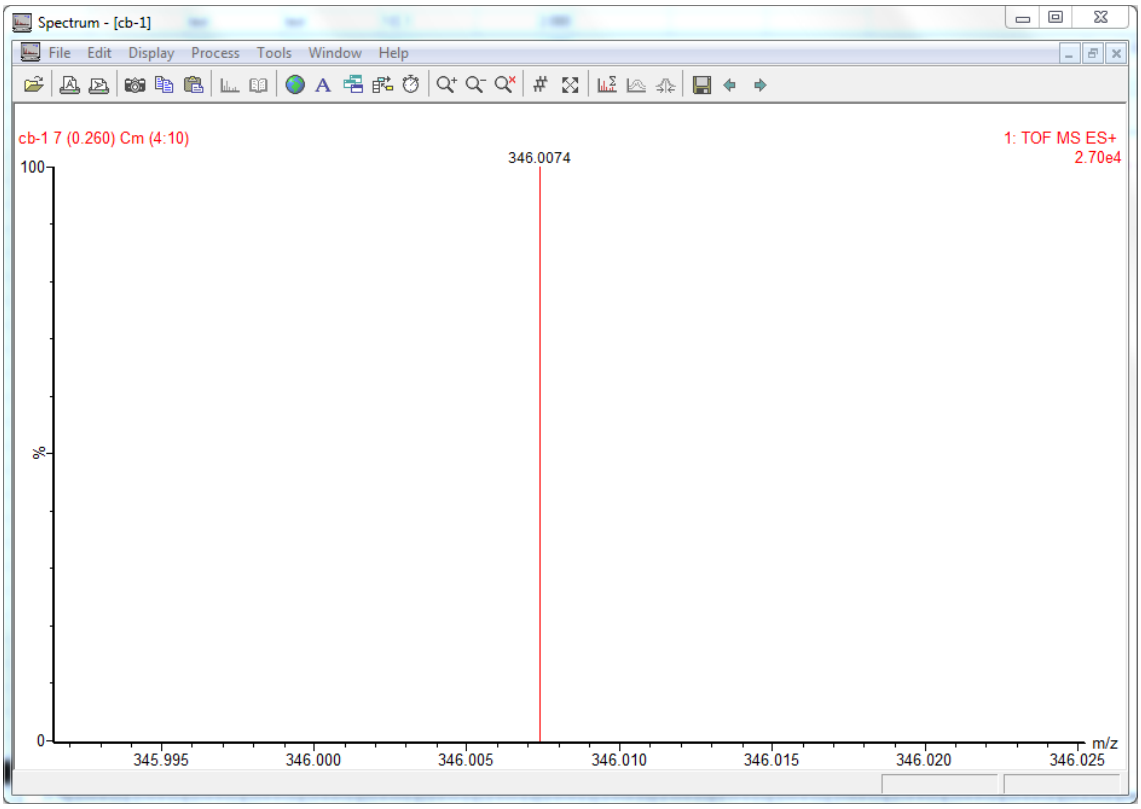

Supplementary Fig50. ESI mass spectrum of ***RS*-PgBrBI**.

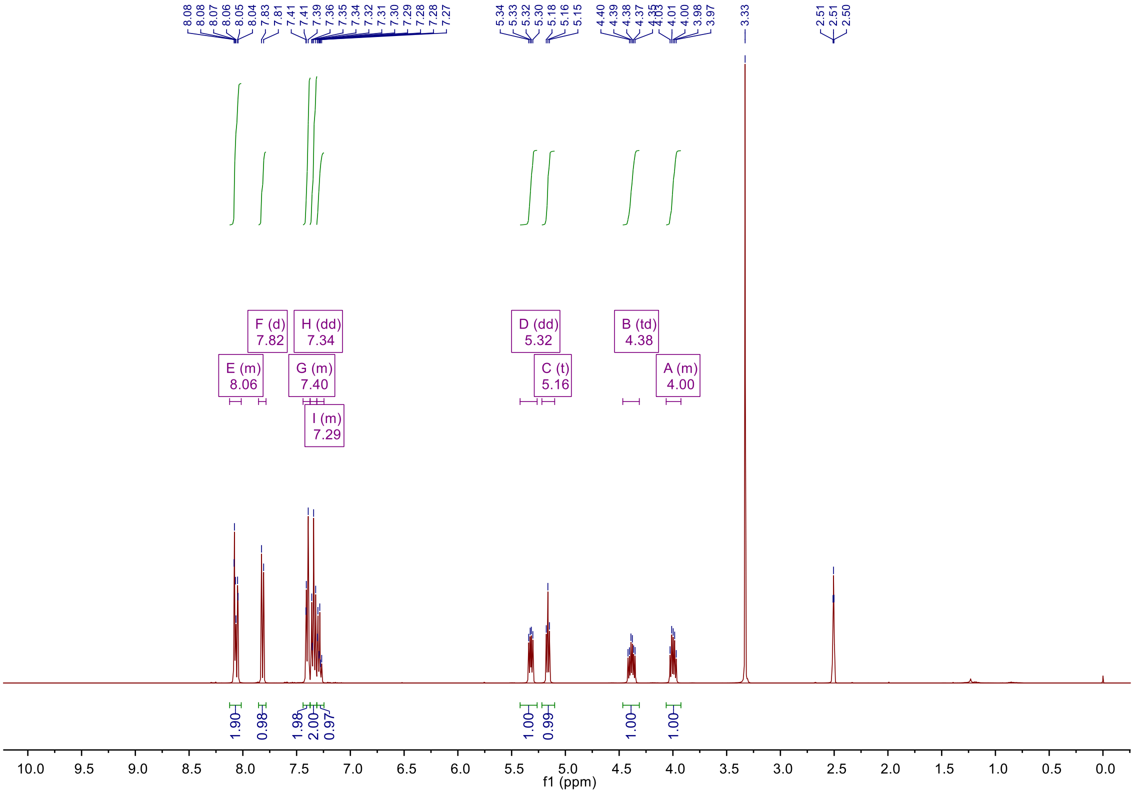

Supplementary Fig51. ^{1}H NMR spectrum of ***R*-PgBrBI** in d-DMSO.

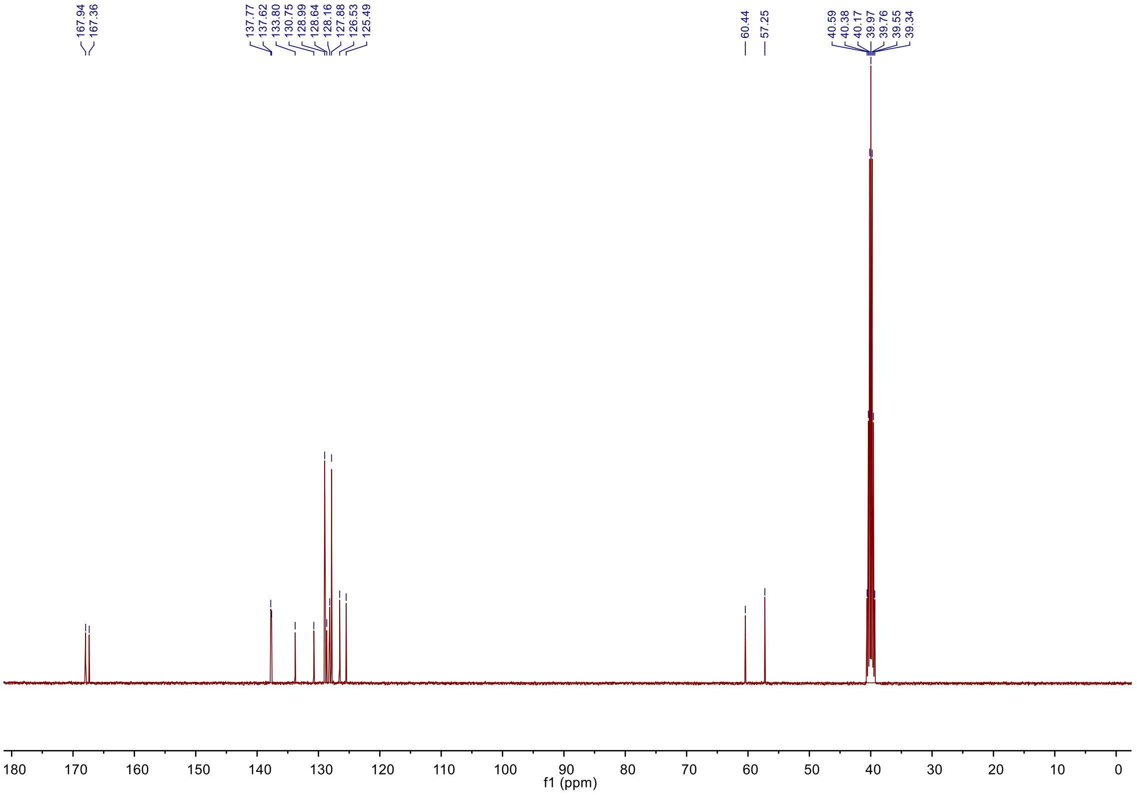

Supplementary Fig52. ^{13}C NMR spectrum of ***R*-PgBrBI** in d-DMSO.

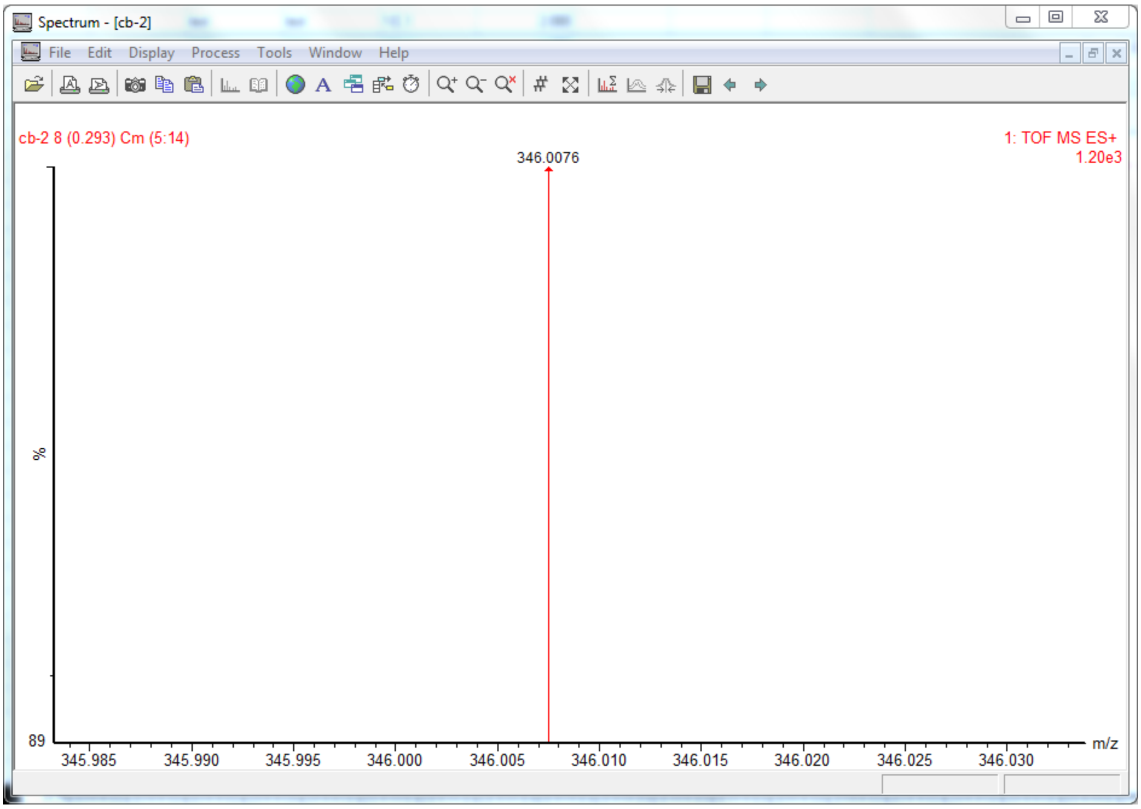

Supplementary Fig53. ESI mass spectrum of ***R*-PgBrBI**.

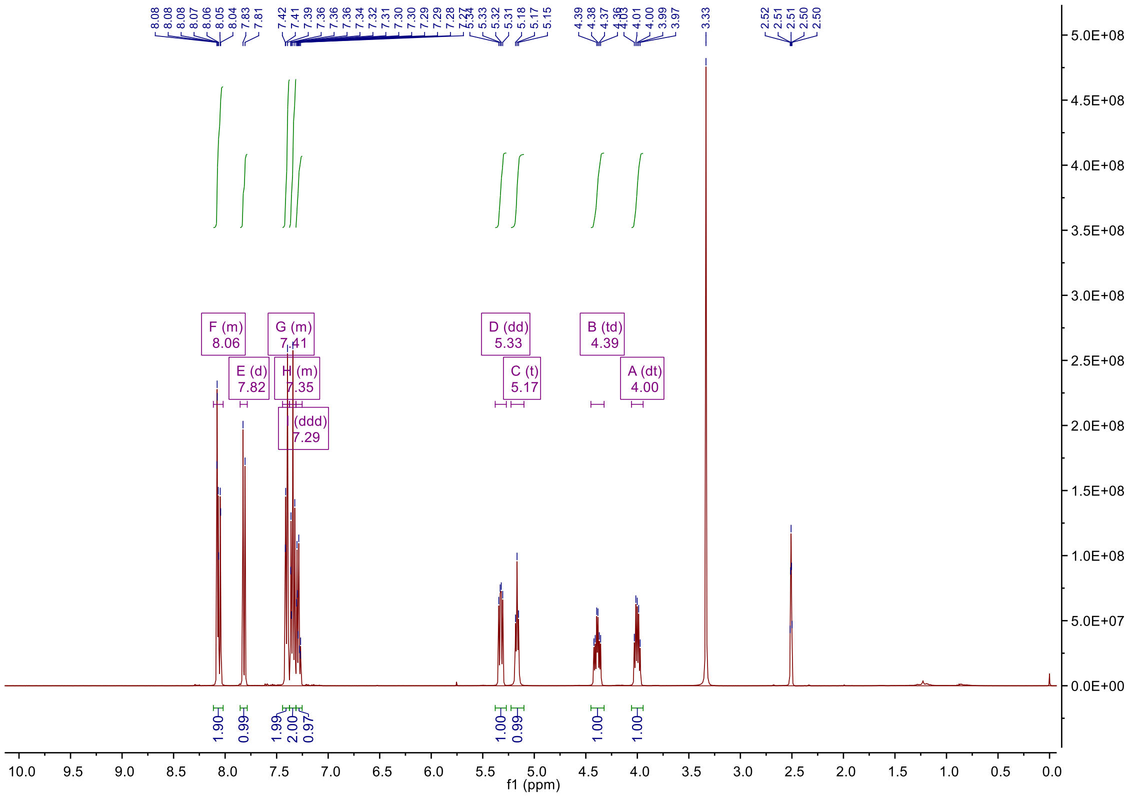

Supplementary Fig54. ^{1}H NMR spectrum of ***S*-PgBrBI** in d-DMSO.

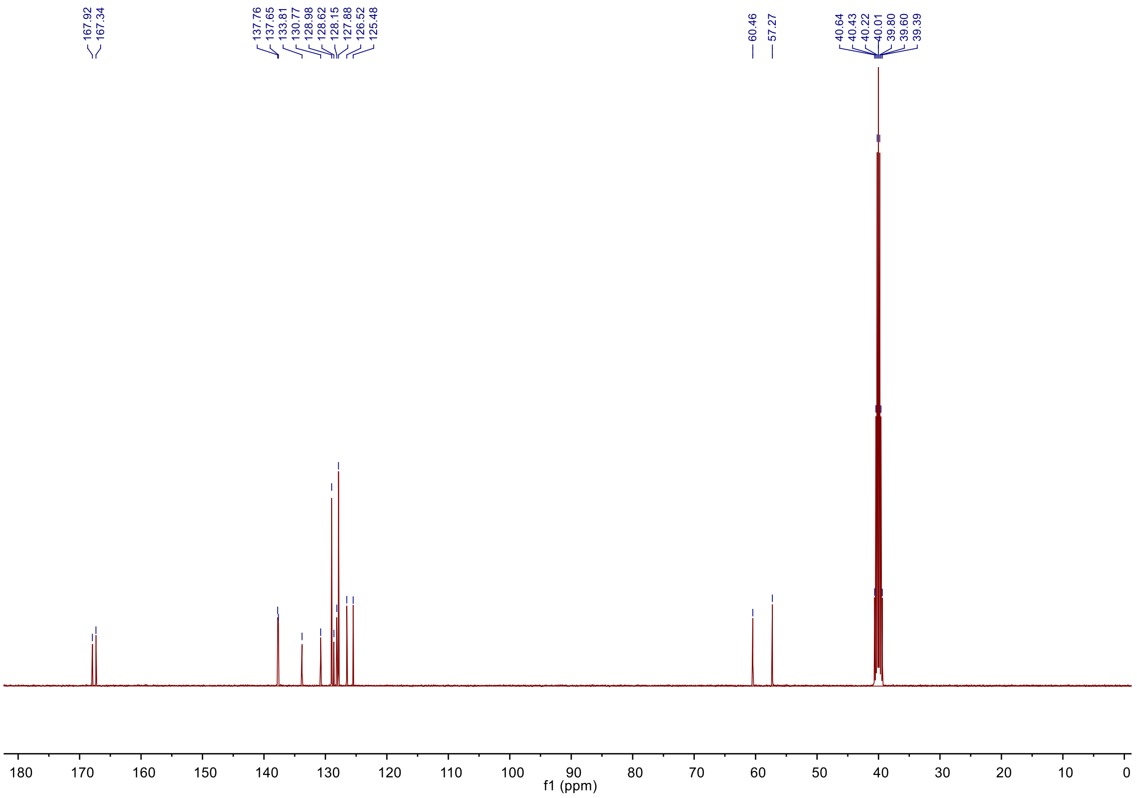

Supplementary Fig55. ^{13}C NMR spectrum of ***S*-PgBrBI** in d-DMSO.

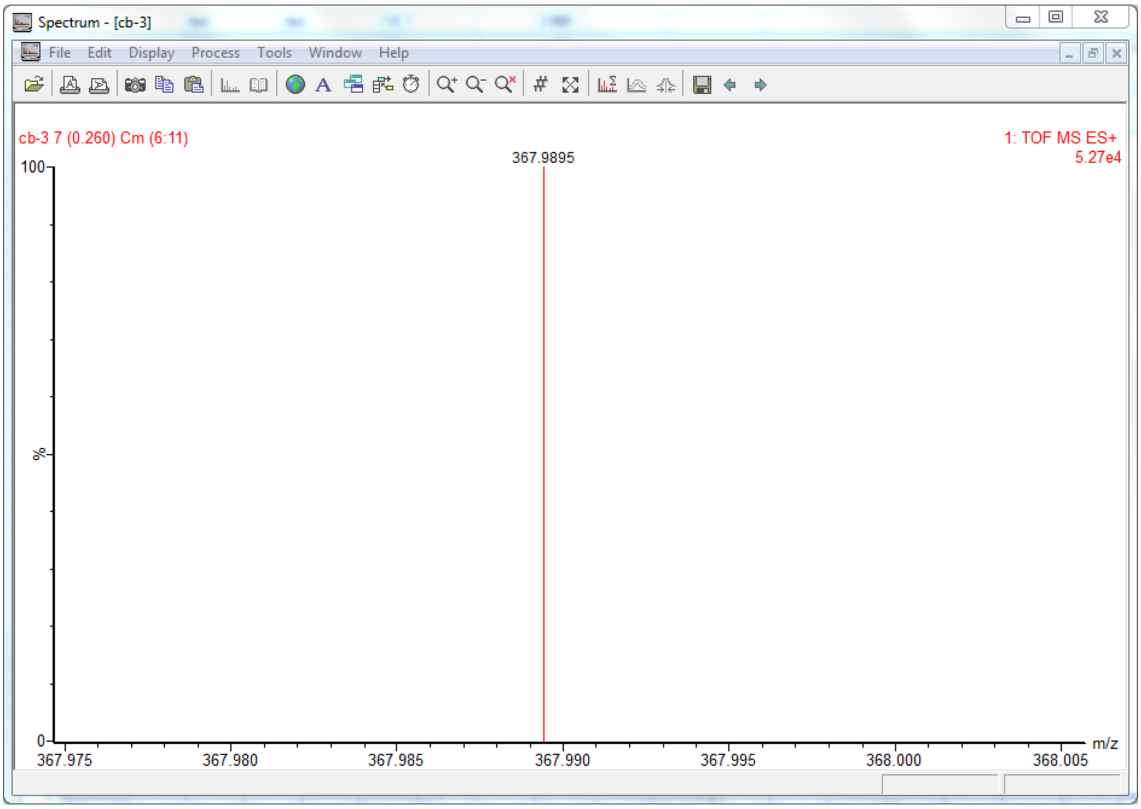

Supplementary Fig56. ESI mass spectrum of ***S*-PgBrBI**.

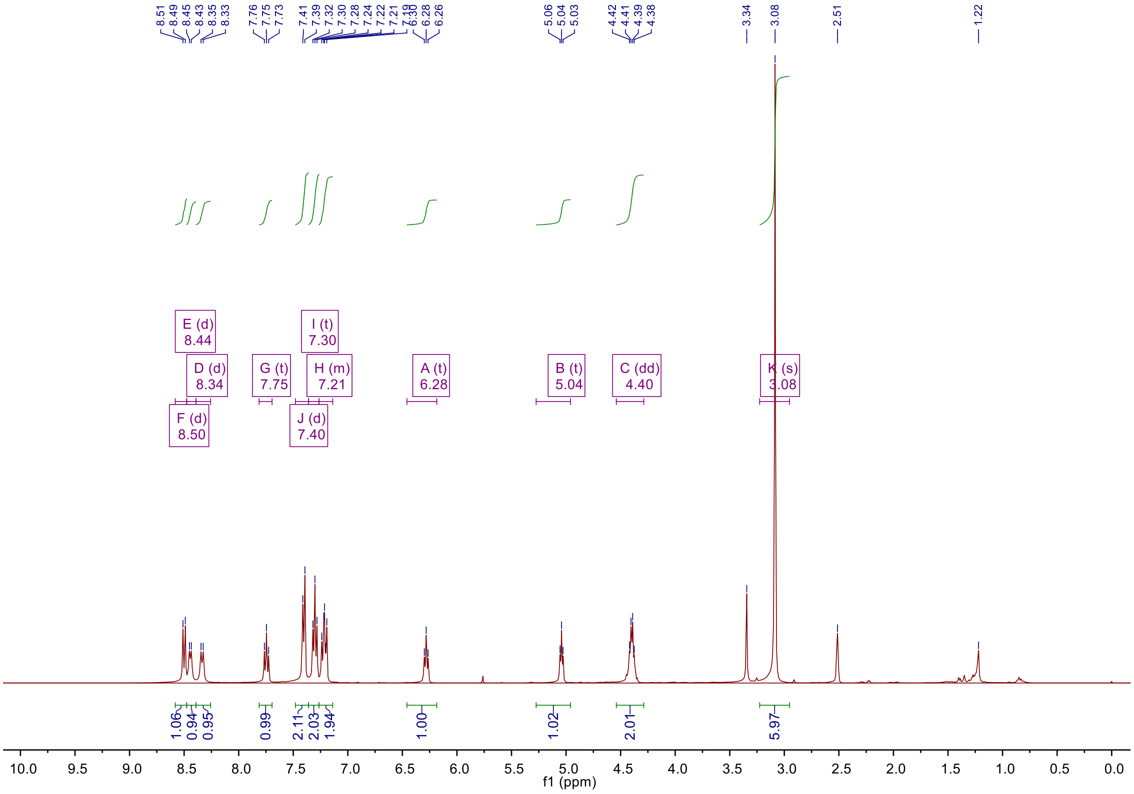

Supplementary Fig57. ^{1}H NMR spectrum of ***RS*-PgMNNI** in d-DMSO.

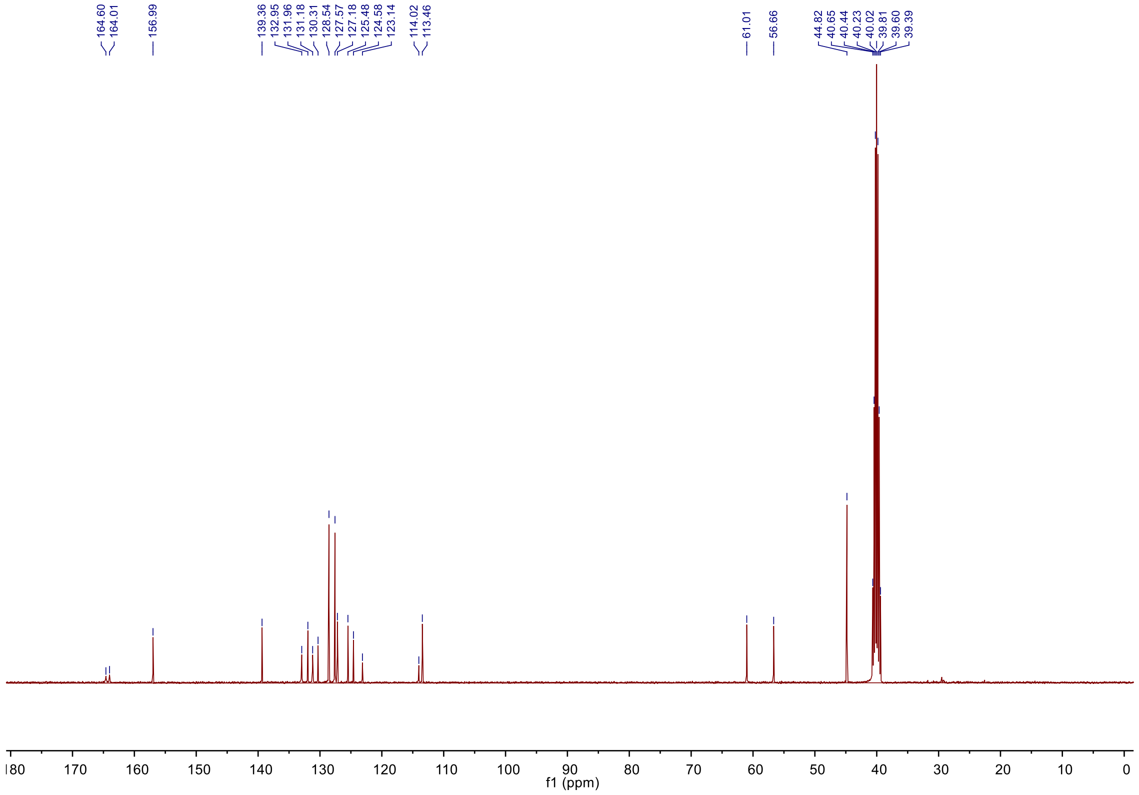

Supplementary Fig58. ^{13}C NMR spectrum of ***RS*-PgMNNI** in d-DMSO.

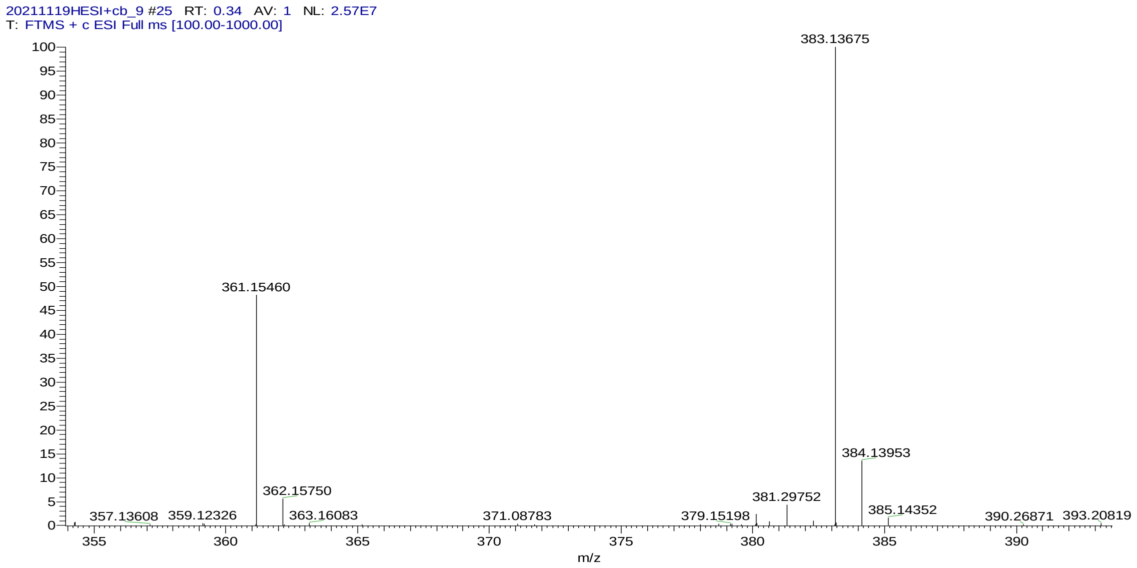

Supplementary Fig59. ESI mass spectrum of ***RS*-PgMNNI**.

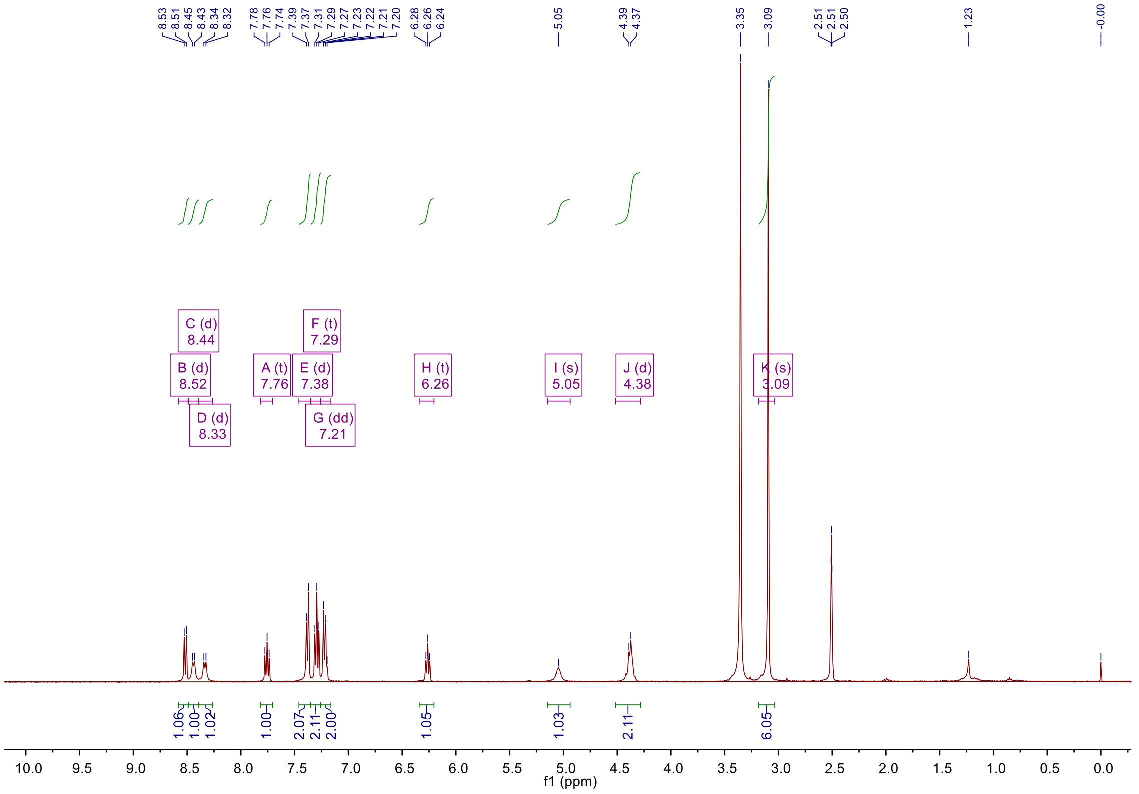

Supplementary Fig60. ^{1}H NMR spectrum of ***R*-PgMNNI** in d-DMSO.

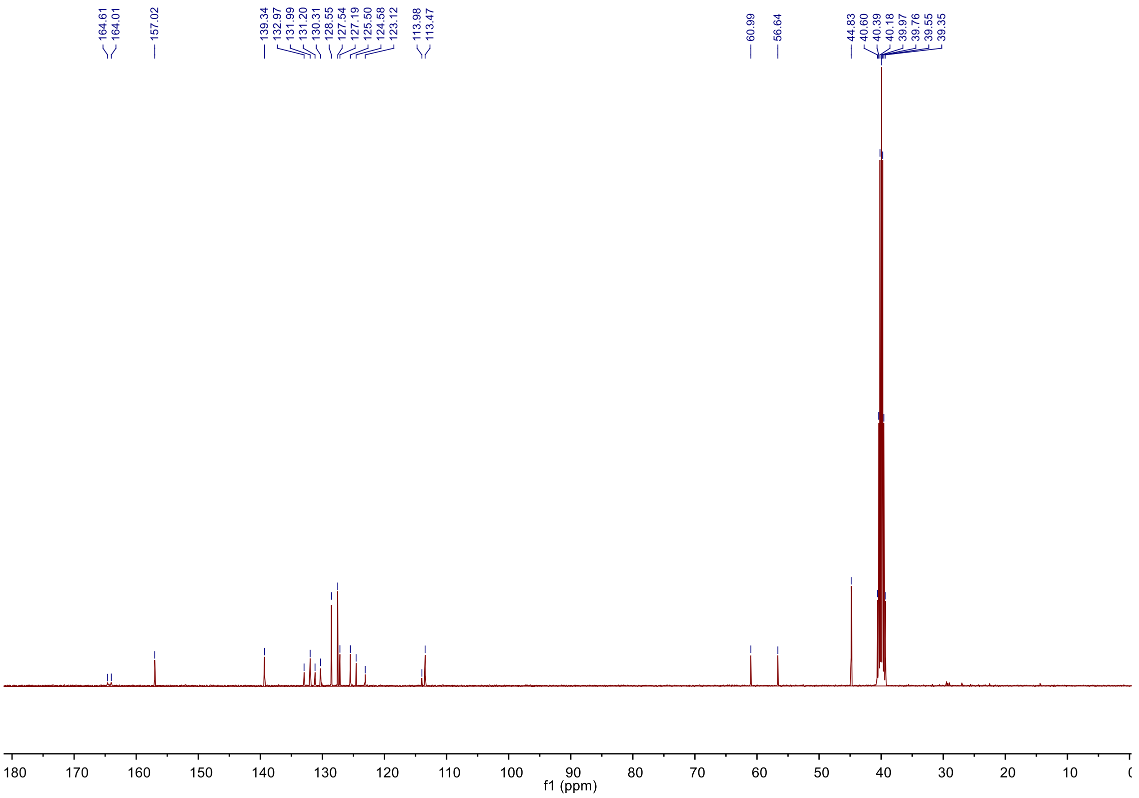

Supplementary Fig61. ^{13}C NMR spectrum of ***R*-PgMNNI** in d-DMSO.

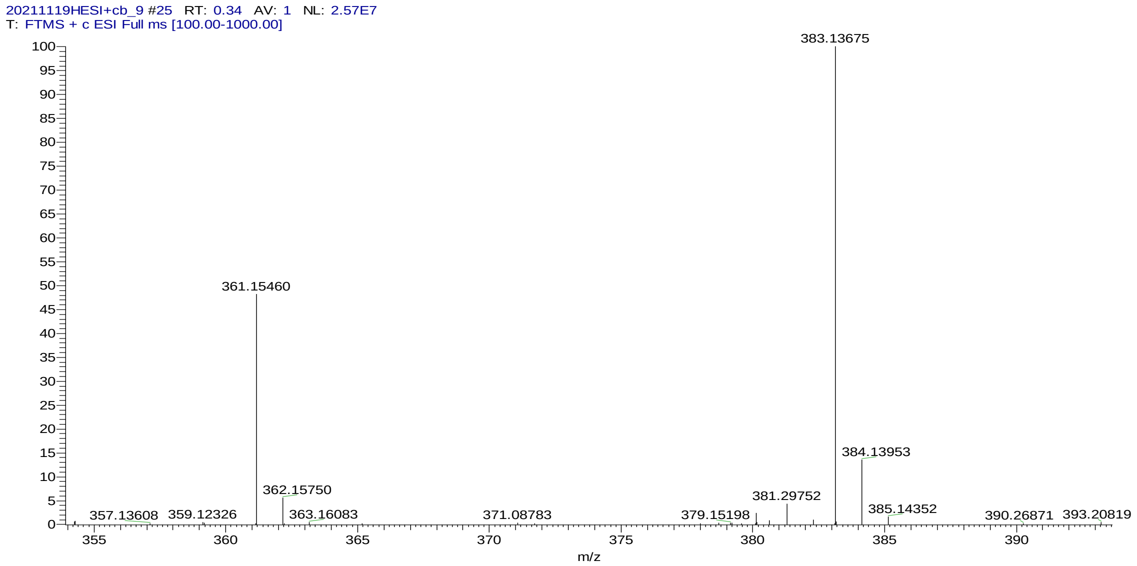

Supplementary Fig62. ESI mass spectrum of ***R*-PgMNNI**.

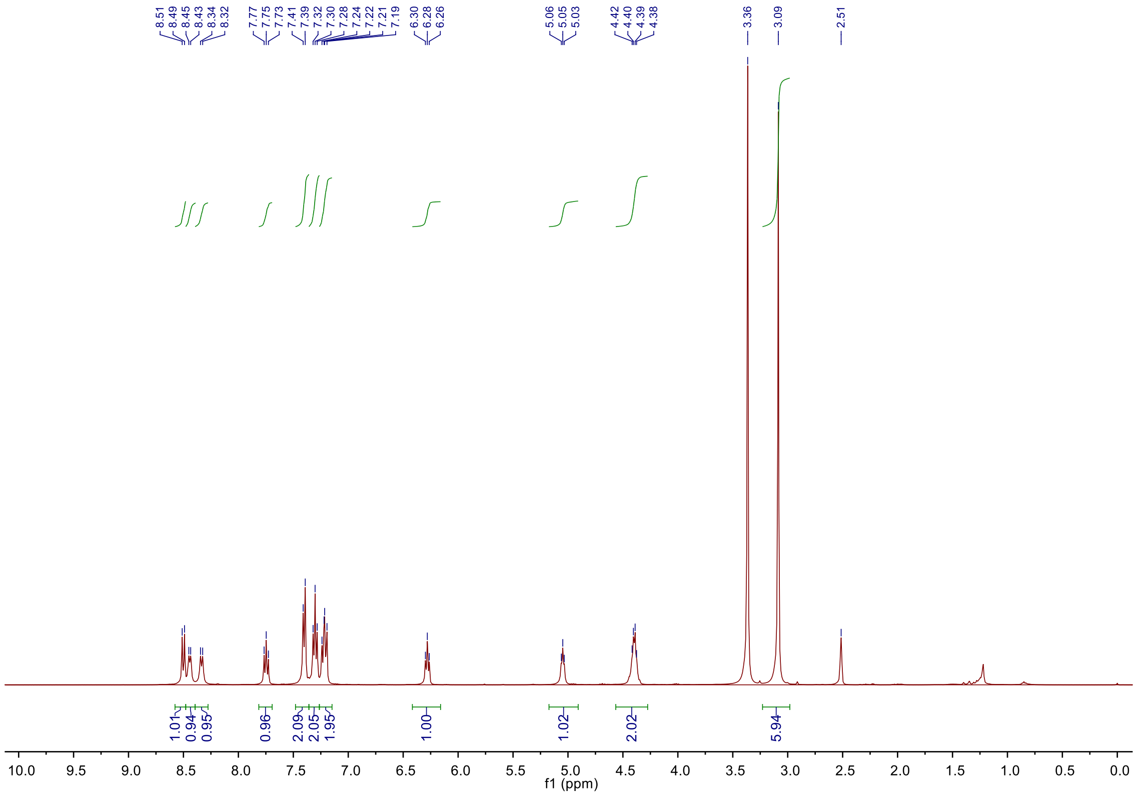

Supplementary Fig63. ^{1}H NMR spectrum of ***S*-PgMNNI** in d-DMSO.

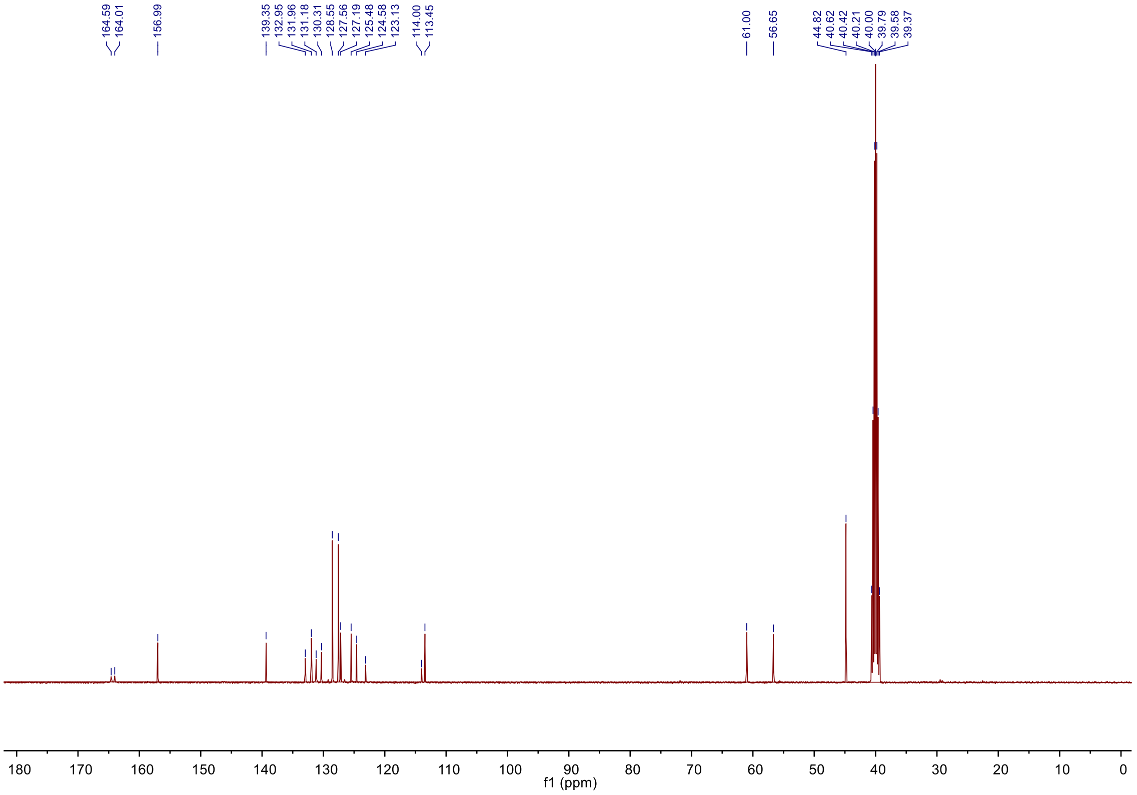

Supplementary Fig64. ^{13}C NMR spectrum of ***S*-PgMNNI** in d-DMSO.

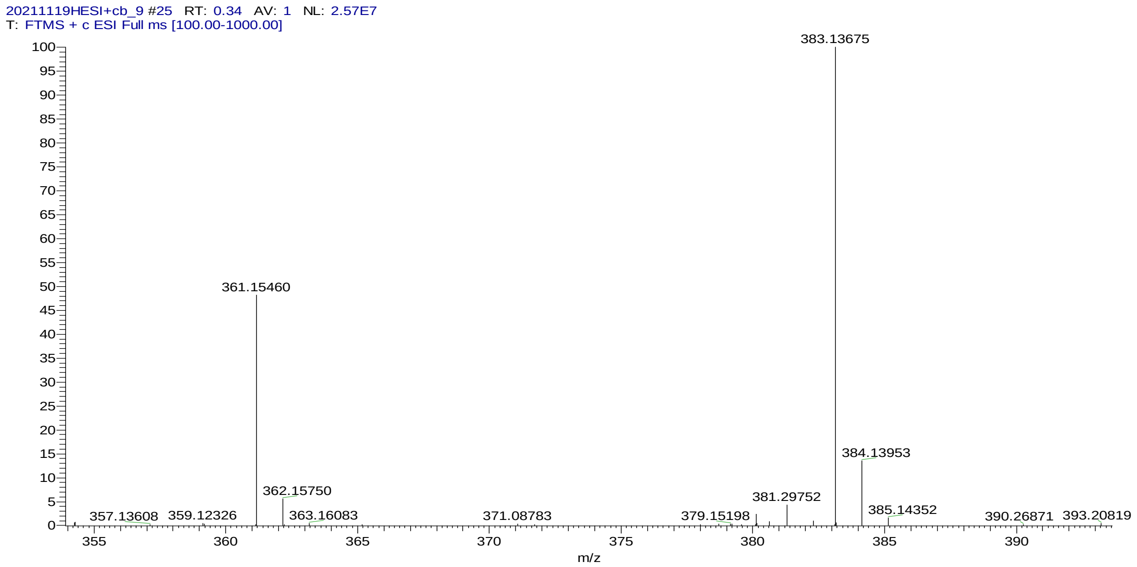

Supplementary Fig65. ESI mass spectrum of ***S*-PgMNNI**.
